# Preservation of organic carbon in marine sediments sustained by sorption and transformation processes

**DOI:** 10.1038/s41561-024-01606-y

**Published:** 2025-01-03

**Authors:** Peyman Babakhani, Andrew W. Dale, Clare Woulds, Oliver W. Moore, Ke-Qing Xiao, Lisa Curti, Caroline L. Peacock

**Affiliations:** 1 School of Earth and Environment, University of Leeds, Leeds, UK; 2 Department of Civil Engineering and Management, University of Manchester, Manchester, UK; 3 GEOMAR Helmholtz Centre for Ocean Research Kiel, Kiel, Germany; 4 School of Geography, University of Leeds, Leeds, UK; 5 Department of Environment and Geography, University of York, York, UK; 6 Research Center for Eco-Environmental Sciences, Chinese Academy of Sciences, Beijing, China

**Keywords:** Carbon cycle, Marine biology

## Abstract

Controls on organic carbon preservation in marine sediments remain controversial but crucial for understanding past and future climate dynamics. Here we develop a conceptual-mathematical model to determine the key processes for the preservation of organic carbon. The model considers the major processes involved in the breakdown of organic carbon, including dissolved organic carbon hydrolysis, mixing, remineralization, mineral sorption and molecular transformation. This allows redefining of burial efficiency as preservation efficiency, which considers both particulate organic carbon and mineral-phase organic carbon. We show that preservation efficiency is almost three times higher than the conventionally defined burial efficiency and reconciles predictions with global field data. Kinetic sorption and transformation are the dominant controls on organic carbon preservation. We conclude that a synergistic effect between kinetic sorption and molecular transformation (geopolymerization) creates a mineral shuttle in which mineral-phase organic carbon is protected from remineralization in the surface sediment and released at depth. The results explain why transformed organic carbon persists over long timescales and increases with depth.

## Abstract (web summary)

Kinetic sorption and transformation are primary controls on organic carbon preservation in marine sediments, according to reactive transport model simulations of the cycling and breakdown of particulate and mineral-phase organic carbon.

## Main

The preservation of organic carbon (OC) in marine sediments is critical to the global carbon and oxygen cycles and, thus, to Earth’s climate and atmospheric composition^1–4^, the distribution of energy resources^5,6^ and finding potential ocean-based mitigation strategies for the removal of excess atmospheric carbon dioxide that drives climate change^7,8^. The controls on carbon preservation, however, are currently unclear^9–11^. In the surface ocean, primary producers or phytoplankton take up atmospheric carbon dioxide to generate biomass, a fraction of which reaches the sediment as particulate OC (POC) and undergoes a series of complex degradation pathways, which may ultimately lead to either carbon remineralization or burial^9,12^. However, this paradigm neglects the role of dissolved OC (DOC) in carbon preservation^13,14^. DOC, which is produced from the hydrolysis of POC, is a key intermediary in carbon cycling before OC is remineralized to inorganic carbon, for example, carbon dioxide. Present concentrations of DOC in marine sediment pore waters, however, may seem low^15^, leading to a lack of knowledge on its role in OC preservation and cycling. Nevertheless, experimental investigations and field measurements^6,12,16–18^ suggest that DOC may accumulate over time as a result of its sorption to minerals forming mineral-phase OC (MOC) and/or its molecular transformation within sediments known as geopolymerization^13,14,16,18–21^, or its dilution in the water column after diffusing out from sediments^22,23^, and may thus substantially impact the carbon cycle on Earth^24,25^.

So far, the contributions of these processes to OC preservation and, thus, carbon cycling have received relatively little attention and are poorly known. Moreover, the conventional concept of OC burial efficiency (BE) in sediments—an indicator of the potential to preserve carbon and quantify global budgets of carbon in modern and ancient sediments^1,4,26–28^—is conceptually incorrect if preservation of OC via DOC sorption and transformation is considerable and ignored. We redefine BE as preservation efficiency (PE), which includes both conventionally considered POC burial and MOC preservation.

Here, we develop a mechanistic reaction-transport model (RTM) that considers the key processes of OC preservation in marine sediments via DOC cycling. After extensive validation, the model is used alongside Monte Carlo and artificial neural network (ANN) analyses to provide global insights into the role of the processes controlling carbon preservation in marine sediments and show where and how preservation occurs.

## Conceptualizing carbon cycling and preservation in sediments

**Fig. 1:**
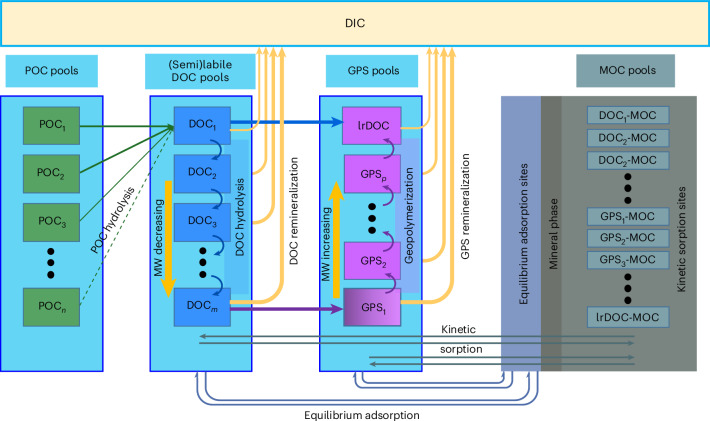
Conceptual model for DOC cycling in sediments. The proposed conceptual model incorporates mechanisms of geopolymerization, equilibrium adsorption and kinetic sorption and a modified concept of hydrolysis that follows DOC cycling in the water column^20^. Model schematic nomenclature includes POC, GPS, lrDOC, various mineral-sorbed DOC ((semi)labile DOC-MOC, GPS-MOC and lrDOC-MOC), DIC and MW. All DOC, GPS and lrDOC pools can interact with minerals through equilibrium adsorption and kinetic sorption. In general, POC pools that originate from the water column can be hydrolysed at any depth in the sediment or remain unhydrolysed. Their transport in the sediment is similar to the sediment solid minerals. MOC pools are transported similarly to sediment solid minerals and POC, but they originate from the net sorption of DOC, GPS and lrDOC to minerals and are further assumed to be unreactive unless the carbon is desorbed from minerals. Part of the POC, which is not hydrolysed at a given depth, and part of the MOC, which is not desorbed at that depth, are considered to be permanently buried.

Our conceptual model for carbon cycling and preservation in sediments begins with the hydrolysis of several discrete POC fractions (POC_1_, POC_2_ and so on) to a single DOC pool (DOC_1_) of high molecular weight (MW) (Fig. 1). Continuing sequential hydrolysis of DOC_1_ produces DOC with increasingly lower MW (DOC_2_, DOC_3_ and so on) and higher reactivity (remineralization rate), analogous to the recent paradigm for DOC cycling in the water column^20^. The sequential hydrolysis approach is markedly different from the previous hypothesis (Fig. 1), where each discrete POC fraction produces a corresponding DOC pool with similar reactivity to its parent POC pool^14^. To represent the production of least-reactive DOC (lrDOC), we include the direct accumulation of freshly hydrolysed but intrinsically undegradable DOC via a direct transfer of DOC_1_ to lrDOC^14^ (Fig. 1). We further consider the geopolymerization of low-MW DOC (DOC_*m*_) to higher-MW molecules herein referred to as geopolymerized substances (GPS-DOC or simply GPS)^21,29^ with increasingly lower reactivity (GPS_1_, GPS_2_ and so on), which eventually contribute to the lrDOC pool (Fig. 1). Our lrDOC pool, thus, comprises both intrinsically undegradable DOC and aged molecularly transformed GPS. Reactivities of DOC pools are selected on the basis of their lifetimes in the water column and are categorized as labile and semi-labile (DOC_1_, DOC_2_ and so on, grouped as (semi)labile-DOC, with a lifetime of ~9 h to ~1.5 years), mid-reactive (GPS_1_, GPS_2_ and so on, with a lifetime of ~20 years) and least-reactive (lrDOC, with a lifetime of ~16,000 years)^24^. To represent the sorption of DOC to minerals, we include a two-site description of ‘equilibrium adsorption’^16,18,19^ and ‘kinetic sorption/desorption’. Kinetic sorption/desorption, which is a general description of sorption/desorption processes that are not in equilibrium, mostly aims to represent occlusion within minerals, co-precipitation and/or aggregation with minerals, and reverse processes that result in the desorption of carbon from the mineral matrix^17,18,30^. The net result of the kinetic sorption–desorption leads to the formation of MOC^31–33^ pools, including (semi)labile DOC-MOC, GPS-MOC and lrDOC-MOC (Fig. 1). These MOC pools are then transported with minerals similar to POC pools that are mixed with minerals, although MOC is different from POC by origin, that is, POC originates from the water column whereas MOC is formed in the sediments via kinetic sorption of DOC to minerals.

We incorporate our conceptual model (Fig. 1) into a vertically resolved RTM for marine sediments that couples transport processes (for example, sediment burial velocity before compaction, and bioturbation mixing)^34^ and biogeochemical reactions (for example, DOC remineralization). We execute the validated RTM in a Monte Carlo approach (>1,000 simulations) using input parameters that are varied randomly within globally relevant ranges (Supplementary Table 1) based on statistical distributions taken from six field-modelling datasets (Supplementary Figs. 1 and 2) and ten previous studies (Supplementary Table 2) to examine the broader role of different OC preservation processes (Supplementary Fig. 3, stage 1).

We then use the RTM-generated dataset to train an ANN^35^ and determine the importance of processes that control OC preservation (Supplementary Fig. 3, stage 2). Our model allows both MOC formation in the solid-phase OC and POC burial at depth to be included in the definition of PE in contrast to the conventional approach where only POC burial is considered in the model^27,28^. The equations for the conventional and the newly defined PE (equations (6) and (7)) and other details of our model development, testing and execution are given in Supplementary Sections 1 and 2. We use PE to evaluate the overall model performance against global data. We further validate our mathematical model and other aspects of our model as summarized in Methods and fully described in Supplementary Section 2, Supplementary Figs. 4–12 and Supplementary Tables 3–7.

## Model evaluation

**Fig. 2:**
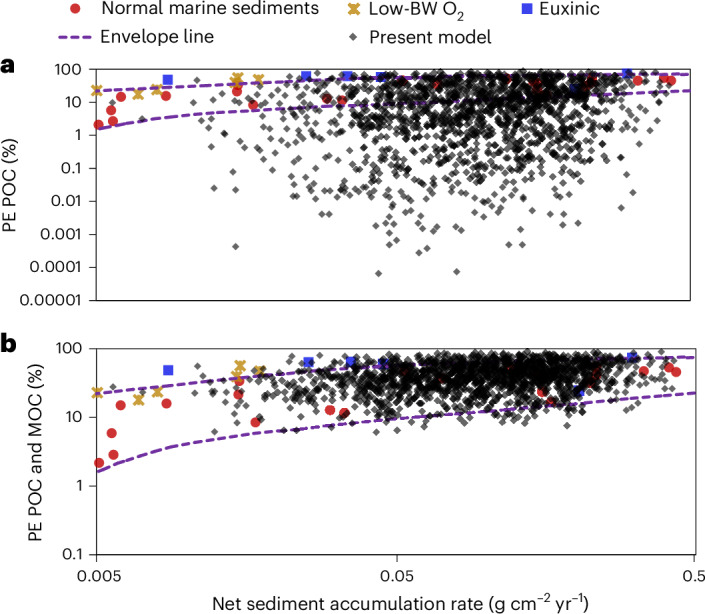
Comparing model-generated PE with literature data. **a**,**b**, Data generated for 1,450 model runs in a Monte Carlo approach (transparent black dots) for the conventional approach to calculate PE of POC that considers only POC (**a**) and for the newly defined PE that considers both POC and MOC (**b**). These are compared with field data from previous studies^12,36^. The spread of model data is derived from a normal distribution of the net sediment accumulation rate data observed in the global grid datasets (Supplementary Figs. 1 and 2). The envelope line (dashed line) represents the general boundaries of the spread of data identified in previous studies^13,41^. Low BW O_2_ stands for low bottom water oxygen concentration.

The incorporation of MOC into models of PE is compared with the classical approach^28,36^ that considers only POC in Fig. 2 (refs. ^12,36^). The PE of POC alone cannot explain the observed trend and envelope of PE values versus sediment accumulation rate (Fig. 2a, dashed lines)^12,36^. Only when the preservation of MOC is considered along with POC does the vast majority of model output data fall inside the envelope (Fig. 2b).

Many different factors have been proposed to explain OC preservation in marine sediments^9,12^. The ability of our model to predict available global PE data indicates that consideration of DOC cycling and related processes, especially sorption and geopolymerization, is critical to predicting PE. This is remarkable because no model parameters were tuned in these simulations (Fig. 2). Our approach of combining RTM, ANN and Monte Carlo allows the process-based understanding of carbon dynamics to be translated into global understanding without the necessity for model fitting to specific sites that may introduce uncertainties related to site-specific conditions.

Our model definition of POC hydrolysis is similar to that considered as POC remineralization elsewhere^9,27,28^, and thus, our estimation for PE of POC is similar to the conventional approach^28^, which also underestimates field PE with estimated global values that are less than 10% (ref. ^28^). Our model, however, additionally tracks DOC hydrolysed from POC and its sorption to minerals, which eventually leads to a much better prediction of PE field data compared with the conventional approach. The mean values for all Monte Carlo model runs (*n* = 1,450) are 16.1 ± 1% and 43.8 ± 1% for PE of POC and POC + MOC, respectively.

## The role of different processes in carbon preservation

We quantify the importance of six model processes in controlling key indicators of carbon preservation, including PE and MOC formation rates (or DOC sorption rates). Three processes are traditionally understood to be important for carbon preservation^37^, namely, DOC hydrolysis to increasingly lower-MW DOC, DOC remineralization and sediment mixing by fauna. The importance of the other three processes is disputed or poorly understood, namely, kinetic sorption, equilibrium adsorption and geopolymerization (Supplementary Tables 8 and 9).

**Fig. 3:**
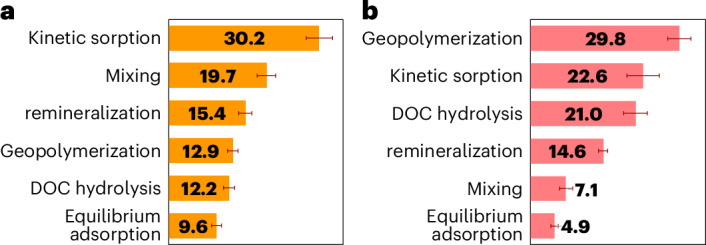
The relative importance of different processes. **a**,**b**, The relative importance (%) of six processes to PE when MOC is considered in addition to POC (**a**) and to preservation rates for MOC (**b**). The six processes are DOC hydrolysis, DOC remineralization, mixing, equilibrium adsorption, kinetic sorption and geopolymerization. The newly defined PE is given by equation (7). The preservation rates for MOC are shown as the rate of MOC formation, which is the sum of net kinetic sorption rates integrated at the depth of 1 m (µmol cm^−2^ yr^−1^) for DOC, GPS and lrDOC. The importance of each process is obtained on the basis of the maximum sensitivity of the parameters categorized for each process. The categorization is presented in Supplementary Table 9. Each bar is the mean of 1,000 executions of the process importance analysis, and the error bars represent the 95% confidence interval. Details of sampling in the Monte Carlo method for process importance analysis are provided in Supplementary Sections 1.3–1.5 and in previous studies^35^.

The results reveal that kinetic sorption is the most important process for PE with a relative importance of 30.2 ± 3% among the six processes, followed by mixing (19.7 ± 2%), remineralization (15.4 ± 1%), geopolymerization (12.9 ± 1%), DOC hydrolysis (12.2 ± 1%) and equilibrium adsorption (9.6 ± 1%) (Fig. 3a). In general, processes that control POC burial flux and carbon turnover at depth, such as mixing and remineralization, are more important for PE than geopolymerization and DOC hydrolysis. We also investigate the most important processes for the preservation rate of DOC-MOC species (Supplementary Fig. 13) that are averaged in Fig. 3b. These show that geopolymerization (29.8 ± 2% relative to all six processes) is the most important process for DOC-MOC preservation, followed by kinetic sorption (22.6 ± 3%), DOC hydrolysis (21.0 ± 2%), remineralization (14.6 ± 1%), mixing (7.1 ± 1%) and equilibrium adsorption (4.9 ± 1%) (Fig. 3b). The highest importance of the geopolymerization process in controlling DOC-MOC preservation adds weight to the existence of a synergic effect of geopolymerization with DOC sorption, in which geopolymerization renders DOC less reactive and sorption provides extra protection from microbial remineralization^18,21^.

Previous considerations of DOC sorption in marine sediments have been limited to a simple equilibrium surface adsorption process expressed using a partition coefficient (*K*_d_), whether in a modelling framework^38,39^ or in field and experimental investigations^16,19^. Others have argued that equilibrium adsorption cannot be responsible for the preservation of intrinsically labile organic compounds and some type of irreversible sorption and/or change in OC reactivity might be responsible^33,40,41^. Proposed patterns, such as monolayer adsorption of DOC onto mineral surfaces^40,42^, still rely on the assumption of mere surface-mediated adsorption and have been criticized for not being able to describe the observed preservation in various ocean settings^33,41,43^. Our consideration of several concurrent processes shows that, while carbon preservation indicators are still not insensitive to the equilibrium adsorption process, their control by this process is the least among the six processes considered (Fig. 3a,b). While equilibrium adsorption can reduce the DOC concentration that is bioavailable in pore fluids, thereby retarding DOC degradation^18,44^, DOC at equilibrium sites can be instantaneously desorbed if the DOC concentrations in the pore water decrease (for example, as a result of microbial degradation or kinetic sorption)^33^. Kinetically controlled mineral DOC protection mechanisms such as co-precipitation, occlusion and aggregation, however, can protect DOC over much longer timescales^31–33^. Our analysis reveals that the kinetic sorption process is the most important factor in controlling PE (Fig. 3a) and the second most important in controlling averaged MOC preservation rates (Fig. 3b).

Recently, it has been shown that geopolymerization in the form of a Maillard-type condensation reaction through catalysis by dissolved or particulate iron and manganese is a crucial process for benthic DOC cycling^21^. Yet, quantification of the geopolymerization process and its importance compared with other processes of carbon preservation has so far remained difficult^10^. Our analysis shows that geopolymerization is equally, or more, important for PE compared with DOC hydrolysis (Fig. 3a). Furthermore, on average, geopolymerization is the most important factor for the preservation rate of DOC species among all six processes considered (Fig. 3b).

## How sorption and geopolymerization control OC preservation

**Fig. 4:**
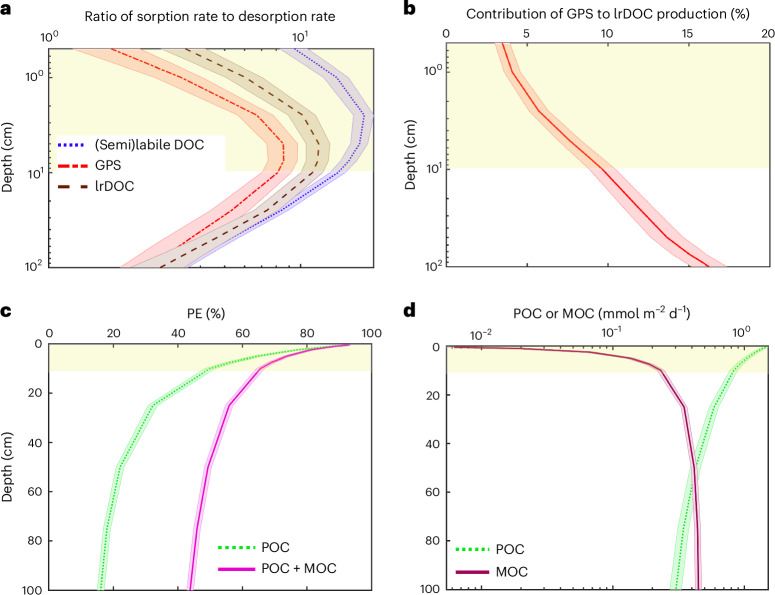
Depth profiles obtained from Monte Carlo modelling. **a**, The ratio of the kinetic sorption rates averaged for 1,450 runs of the Monte Carlo modelling to the desorption rates also averaged for 1,450 model runs at different depths. **b**, The percentage contribution of the final GPS pool to the total lrDOC production (Fig. 1) at different depths. These contributions are averaged for 1,450 runs of the Monte Carlo modelling. The mathematical equations used to produce these plots are presented in Supplementary Section 3. **c**, The PE (%) for only POC and for both POC and MOC together, demonstrating that MOC increases calculated PE by a factor of 2.7 at 1 m depth compared with the conventional approach considering only POC. **d**, POC fluxes and MOC preservation rate. MOC content exceeds POC at a depth of ~50 cm and still continues to rise below this depth. All values have been averaged for 1,450 Monte Carlo model runs. The shaded areas of the curves represent 95% confidence intervals obtained from Monte Carlo model runs. The light-yellow-shaded region represents the mixed layer depth (top 10 cm).

We use sediment depth profiles (Fig. 4 and Supplementary Section 3) obtained from the Monte Carlo simulations to provide a broader insight into how different processes control OC preservation. We observe that the mixed layer acts as a shuttle for different DOC pools by protecting them from exposure to oxygen, nutrients and microbial enzymes and, consequently, limiting their rapid remineralization in the mixed layer and delivering them to greater depths (Fig. 4a). We also investigate the pathways of lrDOC production, which show that geopolymerization contributes 16.3% to lrDOC formation (Fig. 4b). These are fully discussed in Supplementary Section 4.

We further observe that PE can vary with the sediment depth horizon or age horizon^28^. The modelled profiles of PE versus depth in sediment considering only POC as well as POC plus MOC (Fig. 4c) agree with ref. ^28^ that there is a change in PE with depth. However, it appears that this change becomes less discernible below 75 cm. More importantly, MOC formation grows substantially within the top 10 cm and continues to increase below this depth (Fig. 4d) to the point where MOC exceeds the POC content below 50 cm (further discussed in Supplementary Section 4).

In conclusion, our results reveal that, aside from conventionally known processes (hydrolysis, mixing and remineralization), kinetic sorption and geopolymerization probably play a major role in OC preservation. Kinetic sorption creates a mineral shuttle that effectively removes DOC from the active surface layer and releases it at depth, while geopolymerization as an age-dependent process renders OC less reactive. Kinetic sorption leads to the formation of a mineral-associated fraction that becomes larger than the POC pool below ~50 cm, even though the globally averaged POC profile (Fig. 4d, green curve) and PE of POC remain similar to those predicted by the conventional paradigm. Thus, the preservation of OC in marine sediments, hitherto conceived to be due to the burial of POC, is a result of several DOC-related processes, including hydrolysis, geopolymerization and net sorption of DOC to minerals. Consideration of these processes in a single model reconciles the otherwise mismatch of modelled PE with field data and sheds light on the concurrent and complex roles of different processes. We suggest that our conceptualization should be considered in models of carbon cycling and may lead to better quantification and understanding of global carbon budgets, present and past climate dynamics and long-term implications for potential ocean-based carbon dioxide removal technologies^7,8,11^. We acknowledge that, although we have followed the best modelling practice and our model presents advancement in several facets, other models may still be developed in the future that better describe the carbon cycle in marine sediments.

## Methods

### Methodology

#### Overview of the modelling procedure

A RTM is developed to consider the cycling and preservation of DOC in marine sediment. This RTM is then emulated with artificial intelligence, which is used as a tool for conducting a robust process importance analysis. The flowchart of the model deployment is shown in Supplementary Fig. 3. The artificial intelligence technique we use in this study is an ANN, and the RTM is emulated with ANN through a Monte Carlo approach (Supplementary Fig. 3, stage 1), whereby the RTM is executed multiple times (for example, 1,000–2,000) using randomly varied input parameters to obtain the model outputs (for example, the preservation rates for different MOC fractions). These input–output datasets are then used to train an ANN (Supplementary Fig. 3, stage 2)^35^, which allows assessment of the sensitivities of each output to different input parameters grouped into a number of processes.

Among the 68 unknown input parameters of the model, we obtain the ranges and statistical distributions for 6 of the input parameters, including water depth^45^, sediment accumulation rate^46^, sediment surface porosity^47^ and sediment–water interface concentrations of POC, which are assumed to be the same as total OC^47^, NO_3_ (refs. ^48,49^) and O_2_ (refs. ^48,49^) (Supplementary Tables 1 and 8 and Supplementary Figs. 1 and 2) from globally gridded data. For other typical unknown parameters, we compiled data from ten previous studies that had conducted reactive transport modelling on field data (summarized in Supplementary Tables 1 and 2). The ranges for new parameters were also taken either from the literature^24^ or from fitting our model to the field-modelling data from the literature (Burdige et al.^14,50^). Although we have tested the RTM against field datasets and have selected the ranges for some of the new model parameters by considering the calibrated parameter values (Supplementary Section 2.2), such model fittings to field datasets are useful but not necessary. This is because the RTM, which is based on established or hypothetical concepts, already embodies process-based knowledge in the approach, and the additional application of ANN and Monte Carlo allows the use of parameter ranges instead of set values. A more detailed description of the overall modelling procedure is provided in Supplementary Section 1.1.

#### Formulation of the RTM

The RTM considers all common early diagenesis reactions for different compounds including dissolved species (O_2_, SO_4_, NH_4_, NO_3_, dissolved inorganic carbon (DIC), H_2_S, CH_4_, Fe^2+^, Mn^2+^, DOC_1_ to DOC_*m*_, GPS_1_ to GPS_*p*_, and lrDOC, where subscript *m* represents the maximum number of DOC pools and subscript *p* represents the maximum number of GPS pools) and particulate species (highly reactive iron oxide, Fe(OH)_3_^HR^, moderately reactive iron oxide, Fe(OH)_3_^MR^, non-reactive iron oxide, Fe(OH)_3_^UR^, MnO_2_, FeS, FeS_2_, S^0^, and POC_1_ to POC_*n*_, where subscript *n* represents the maximum number of POC pools) as listed in Supplementary Table 10. Here, we considered seven species for POC, four species for DOC and two species for GPS in addition to lrDOC. The number of different carbon species are selected mainly on the basis of consistency with previous literature^24^ and with consideration of other aspects of modelling, including alignment with the conceptual model, smooth transition of rates across different species for numerical solution considerations and minimizing the number of unknown model parameters. It should be noted that, as demonstrated in the Supplementary Section 1.2.2, the selection of the number of these species, for example, DOC pools, per se, does not affect the model outputs noticeably.

The three governing equations of RTM for dissolved species, particulate species and sorbed species, respectively, are as follows, while the full details of the model development and validation are provided in Supplementary Sections 1 and 2:

1. The governing equation for dissolved species:
  1 $$\begin{array}{l}\left(\varphi +{\rho }_{{\mathrm{s}}}\varepsilon {{K}_{{\mathrm{d}}}}_{i}\right)\frac{\partial {{C}_{{\mathrm{d}}}}_{i}}{\partial t}=\frac{\partial }{\partial z}\left((\varphi D+{\rho }_{{\mathrm{s}}}\varepsilon {{K}_{{\mathrm{d}}}}_{i}{D}_{{\mathrm{b}}})\frac{{\partial {C}_{{\mathrm{d}}}}_{i}}{\partial z}\right)\\\qquad\qquad\qquad\qquad-\frac{\partial }{\partial z}\left(\left(\varphi {v}_{{\mathrm{d}}}+{\rho }_{{\mathrm{s}}}\varepsilon {{K}_{{\mathrm{d}}}}_{i}{v}_{{\mathrm{p}}}\right){{C}_{{\mathrm{d}}}}_{i}\right)+\varphi \alpha \left({{C}_{{\mathrm{d}}}}_{i}\left(0\right)-{{C}_{{\mathrm{d}}}}_{i}\left(Z\right)\right)\\\qquad\qquad\qquad\qquad+\varphi \sum _{j=1}{{R}_{{\mathrm{d}}}}_{i,\,j}-{{k}_{{{\mathrm{sorp}}}}}_{i}{{C}_{{\mathrm{d}}}}_{i}+\frac{{{k}_{{{\mathrm{sorp}}}}}_{i}}{{{{{\mathrm{Kd}}}}_{{{\mathrm{sorp}}}}}_{i}}{{S}_{{\mathrm{d}}}}_{i}\end{array}$$
2. The governing equation for particulate species:
  2 $${\rho }_{{\mathrm{s}}}\varepsilon \frac{\partial {{C}_{{\mathrm{p}}}}_{i}}{\partial {{t}}}={\rho }_{{\mathrm{s}}}\frac{\partial }{\partial z}\left(\varepsilon {D}_{{\mathrm{b}}}\frac{{\partial {C}_{{\mathrm{p}}}}_{i}}{\partial z}\right)-{\rho }_{{\mathrm{s}}}\frac{\partial }{\partial z}\left(\varepsilon {v}_{{\mathrm{p}}}{{C}_{{\mathrm{p}}}}_{i}\right)+{\rho }_{{\mathrm{s}}}\varepsilon {{R}_{{\mathrm{p}}}}_{i}$$
3. The governing equation for mineral phase, MOC, resulting from the kinetically sorbed fraction of dissolved species:

3 $$\begin{array}{l}{\rho }_{{\mathrm{s}}}\varepsilon \frac{\partial {{S}_{{\mathrm{d}}}}_{i}}{\partial t}={\rho }_{{\mathrm{s}}}\frac{\partial }{\partial z}\left({\varepsilon D}_{{\mathrm{b}}}\frac{{\partial {S}_{{\mathrm{d}}}}_{i}}{\partial z}\right)-{\rho }_{{\mathrm{s}}}\frac{\partial }{\partial z}\left(\varepsilon {v}_{{\mathrm{p}}}{{S}_{{\mathrm{d}}}}_{i}\right)+{\rho }_{{\mathrm{s}}}\varepsilon \sum _{j=1}{{R}_{{{\mathrm{Sd}}}}}_{i,\,j}\\\qquad\qquad+{{k}_{{{\mathrm{sorp}}}}}_{i}{{C}_{{\mathrm{d}}}}_{i}-\frac{{{k}_{{{\mathrm{sorp}}}}}_{i}}{{{{{\mathrm{Kd}}}}_{{{\mathrm{sorp}}}}}_{i}}{{S}_{{\mathrm{d}}}}_{i},\end{array}$$

where *C*_d*i*_ is the concentration of dissolved species *i* (mM or µmol cm^−3^ of pore water), *C*_p*i*_ is the concentration of particulate species *i* (g g^−1^), *S*_d*i*_ is the concentration of dissolved species *i* kinetically sorbed to sediment minerals (µmol g^−1^ of solid sediments), *φ* is porosity, *ε* is the solid fraction of sediments, which is equal to 1 − *φ*, *v*_d_ and *v*_p_ are the burial velocities of pore water and particulate species (cm yr^−1^), *ρ*_s_ is the dry density of sediments (g cm^−3^), *D*_*i*_ is the apparent diffusion coefficient of dissolved species *i* (cm^2^ yr^−1^), *α* is the bio-irrigation coefficient (cm^2^ yr^−1^), *D*_b_ is the bioturbation coefficient (cm^2^ yr^−1^), *z* is the sediment depth with respect to the coordinate system located at the sediment–water interface (cm), *R*_p_, *R*_d_ and *R*_Sd_ stand for reaction rates of particulate, dissolved and kinetically sorbed species (yr^−1^, µmol cm^−3^ yr^−1^ and µmol g^−1^ yr^−1^), respectively, which are temporally and spatially variable, *k*_sorp_ is the mass transfer rate between the dissolved and kinetically sorbed phases to minerals (MOC pools) (yr^−1^), and Kd_sorp_ is the so-called distribution coefficient in the kinetic mass transfer expression (cm^3^ g^−1^).

The first stage of the hydrolysis is considered similar to the conventional first-order multi-POC degradation model known as the multi-G model^51^ with a series of POC pools converting to a single DOC pool, DOC_1_, in parallel:

4 $${\left\{\frac{\partial {{C}_{{\mathrm{p}}}}_{i}}{\partial {{t}}}\right\}}_{{{\mathrm{Hydrolysis}}}}={{R}_{{\mathrm{p}}}}_{i}={k}_{i}{{C}_{{\mathrm{p}}}}_{i},$$

where *k*_*i*_ is the hydrolysis rate constant, which was considered in a similar way to the degradation rate constants of POC in the continuum model following previous studies^9,34,52^.

The sequential stage of the hydrolysis has been described using a consecutive first-order reaction expression^20,53^:

5 $${\left\{\varphi \frac{\partial {{C}_{{\mathrm{d}}}}_{i}}{\partial {{t}}}\right\}}_{{{\mathrm{Hydrolysis}}}}={\left\{\varphi \sum _{j=1}{{R}_{{\mathrm{d}}}}_{i,\,j}\right\}}_{{{\mathrm{Hydrolysis}}}}={\lambda }_{{{\mathrm{DOC}}_{i-1}}}{{C}_{{\mathrm{d}}}}_{i-1}-{\lambda }_{{{\mathrm{DOC}}_i}}{{C}_{{\mathrm{d}}}}_{i},$$

where *λ*_DOC*i*_ is the conversion rate of DOC_*i*_ to DOC_*i*+1_, and *λ*_DOC*i−*1_ is the conversion rate of DOC_*i*−1_ to DOC_*i*_ in yr^−1^.

The same mathematical formula is used to describe geopolymerization^54,55^ as provided in Supplementary Section 1 along with the other details.

#### Calculation of PE

PE, elsewhere known as BE, conventionally considered for POC^28,36^ is defined as follows:

6 $${{\mathrm{PE}}}=\frac{{\rm{POC}}\; {\rm{flux}}\; {\rm{at}}\; {\rm{depth}}\,{L}}{{\rm{Total}}\; {\rm{POC}}\; {\rm{flux}}\; {\rm{at}}\; {\rm{sediment}}\; {\rm{surface}}}\times 100,$$

where *L* is a given depth herein considered as 1 m. In the present study, owing to the full consideration of the fate of DOC in our model, we are able to present a more accurate consideration of PE that includes the fraction of solid phase OC that has undergone hydrolysis and sorption to minerals:

7 $${{\mathrm{PE}}}=\frac{{\rm{POC}}\; {\rm{flux}}\; {\rm{at}}\; {\rm{depth}}\,{L}+{\rm{Sorption}}\; {\rm{rate}}\;{\rm{integrated}}\; {\rm{over}}\; {\rm{depth}}\,{L}}{{\rm{Total}}\; {\rm{POC}}\; {\rm{flux}}\; {\rm{at}}\; {\rm{sediment}}\; {\rm{surface}}}\times 100.$$

The sorption rate is the net DOC kinetic sorption rate (sorption rate minus desorption rate or the net MOC formation rate). Further explanations about the rates are provided in Supplementary Sections 2.4 and 3. Although the consideration of depth-versus-age horizons in early digenesis modelling can be important for global predictions, as was recently highlighted^28^, in the present study, we considered only a constant depth horizon as the aim is to obtain insight into the processes that control OC preservation rather than making global predictions.

#### ANN for process importance analysis

The ANN is a versatile and universal tool for function approximation problems and is notable for its application to complex, nonlinear systems^35,56^. The commonly used ANN structure is a three-layer configuration comprising input, hidden and output layers^35,57–59^. Each of these layers is composed of a series of nodes (neurons) with their numbers in the input and output layers corresponding to the number of input and output variables, respectively. The number of neurons on the hidden or middle layers should be optimized when finding the best fit during the training process^57,60^. The main equation used for processing the information (or signal) in the structure of the ANN is a simple algebraic equation in the form of *y* = *w* × *x* + *b*, which applies to each neuron in the hidden layer. The information is then summed up for all nodes; additional functions called transfer (or activation) functions that are exerted on the input and output information that are detailed elsewhere^35,57^. Here, *x* stands for the input information (or signal), *y* stands for output information, *w* is weights and *b* is biases. Weights and biases are the hyperparameters of the ANN, which are determined after fitting ANN to data, and once they are determined, they form an empirical network that can be used for new predictions. In the scope of the present study, we use ANN only for process importance analysis, not for prediction. Here, we use the partial derivative method^35,57,58,61^ for process importance analysis. In brief, in this method, the derivatives of the equations used in the structure of ANN are used to represent the role of each ANN input in controlling the ANN output; for example, for the main equation *y* = *w* × *x* + *b*, the derivative is equal to *w*. In this way, the *w* value for each neuron represents the strength of the signals passing through that neuron^35,57–59^. This implies that in the process importance analysis, the input parameter values do not play a marked role; rather, it is their variations that are important and reflected in the structure of the ANN. Details of the ANN model used here have been selected following ref. ^35^ and are described in Supplementary Section 1.5.

#### Model validation

We validate our model in a number of ways. We validate our developed governing equations of the RTM on the basis of an analytical approach. In this approach, for the condition where equilibrium adsorption and kinetic sorption are expected to behave similarly, that is, at high exchange rates, we first run the model after turning off equilibrium adsorption and then run the model again turning off the kinetic sorption expression. Then, the model outputs for these two types of simulation are compared. We used existing field-modelling data (principally from Santa Barbara Basin, given the comprehensive dataset available)^14,62^ as shown in Supplementary Figs. 5–11 and Supplementary Table 6. We also validated the model on the basis of mass budgets. The use of the ANN is validated on the basis of its ability to describe the data (measured using goodness-of-fit criteria described in Supplementary Section 2.2) and using the uncertainties it yields in the process importance analysis. Finally, validation of the overall RTM modelling process was carried out using mass budgets of the averaged model outputs over the multiple runs of the Monte Carlo. This was done using the concept of mass flow in the model illustrated in Supplementary Fig. 14.

The results of the model output comparison between the cases when kinetic sorption is operative and when the equilibrium adsorption is operative at a high mass transfer rate show an excellent match (*R*^2^ = 1.000; Supplementary Fig. 4) verifying our approach towards development and implementation of sorption formulation in the governing equations.

The results of the model fit to several field or modelling datasets, including Meysman et al.^62^ (Supplementary Fig. 5), Kraal et al.^63^ (Supplementary Figs. 6 and 8 and Supplementary Tables 3–5) and Burdige et al.^14,50^ (Supplementary Figs. 9–11 and Supplementary Tables 6) show excellent matches between our model and existing field-model datasets for most of the concentration-versus-depth profiles. The exceptions are generally FeS_2_ and Mn^2+^ profiles, which show poorer model fit due to the lack of carbonate species in our model. The added complexity of our model (more unknown parameters) is validated in eight steps against the Santa Barbara Basin dataset^14,50^ (Supplementary Table 6), showing that each step is justifiable in terms of improvements in model fits to the data for the cost of complexity, according to model selection criteria^64^ increasing from 0.626 in step 2 to 0.843 in step 7. Burdige et al.^14,50^ further considered *δ*^13^C, Δ^14^C and carbon-to-nitrogen ratios in their model and matched them with field measurements that are not conducted here.

The ANN model could fit the data in all cases with the best predictive fit Nash–Sutcliffe model efficiency criterion^65^ ranging from 0.923 to 0.944 (Supplementary Table 7 and Supplementary Fig. 12). The uncertainties in the ANN process importance analysis determined as a 95% confidence interval were relatively minor (see error bars in Fig. 3). These uncertainties range from 4.6% to 29.9% (12.6% on average) of the mean values for the cases investigated and shown in Fig. 3 and Supplementary Fig. 13.

The mass budgets for different cross-sections of the simplified conceptual model shown in Supplementary Fig. 14 were calculated on the basis of averaged results of all Monte Carlo model runs (1,450) at stage 1. According to these results, the mass budget in cross-section A–A is MB_A–A_ = 57.136 µmol cm^−2^ yr^−1^, in B–B is MB_B–B_ = 57.197 µmol cm^−2^ yr^−1^ and in C–C is MB_C–C_ = 57.189 µmol cm^−2^ yr^−1^, demonstrating an overall mass balance error of ~0.1%, which is less than the acceptable mass balance error of 1% considered in our general modelling process. It should be noted that, despite our extensive model validation process, in the present study, we use the model only for process importance analysis and finding insight into the underlying processes responsible for OC preservation, not for making global predictions, which is the subject of future study. Finally, based on acceptable uncertainties (95% confidence interval) related to the sum of all >1,000 RTM runs, shown as the shaded area around the curves in Fig. 4 and Supplementary Fig. 16, and the uncertainties of process importance analysis obtained from the ANN stage shown in Fig. 3, our general approach of random variation of input parameters is appropriate.

It should be noted that limitation in the capacity of sorption sites, for example, monolayer sorption^16,40^, typically does not apply to kinetic sorption because the kinetic sorption model in the present study mainly represents the processes that internalize DOC into the mineral matrix, such as occlusion, co-precipitation and aggregation, and thus, limited-capacity sorption considered in the literature mostly through the monolayer surface adsorption hypothesis is not applicable to our MOC production. Furthermore, kinetic sorption is slower than equilibrium adsorption, which is known to be instantaneous. Thus, kinetic sorption, which is limited by pore water concentrations that are also controlled by hydrolysis, degradation and so on, is less likely to face a second limitation by the capacity of sorption sites compared with instantaneous equilibrium adsorption for which different types of isotherm, such as linear, Langmuir and Freundlich, have been defined^66,67^. Adding an additional parameter to force a proportion of the OC to be taken up by the kinetic and equilibrium sorption sites would add more unknown parameters and is deemed unnecessary in this case.

## Online content

Any methods, additional references, Nature Portfolio reporting summaries, source data, extended data, supplementary information, acknowledgements, peer review information; details of author contributions and competing interests; and statements of data and code availability are available at 10.1038/s41561-024-01606-y.

## Supplementary information

Supplementary Information Supplementary Sections 1–5, Figs. 1–18 and Tables 1–10.

## Data Availability

Data associated with Figs. 2–4 are available via figshare at 10.6084/m9.figshare.27273006 (ref. ^68^). Data related to Supplementary Figs. 13, 15 and 16 and the main datasets (such as those generated by RTM through the Monte Carlo process (stage 1) and used in the ANN process importance analysis (stage 2)) are available via figshare at 10.6084/m9.figshare.27273030 (ref. ^69^).

## Code availability

Computer codes are available from the authors upon request.
