# Supplementary material for: Preservation of organic carbon in marine sediments sustained by sorption and transformation processes
Source: Nat Geosci. 2025 Jan 3;18(1):78–83. doi: 10.1038/s41561-024-01606-y (PMC11732750; doi:10.1038/s41561-024-01606-y)
Supplement: Supplementary file 1 — Supplementary Sections 1–5, Figs. 1–18 and Tables 1–10. [file 41561_2024_1606_MOESM1_ESM.pdf]

nature geoscience

Supplementary information https://doi.org/10.1038/s41561-024-01606-y

Preservation of organic carbon in marine sediments sustained by sorption and transformation processes

In the format provided by the authors and unedited

Table of Contents

S1. Details of model development 2
S1.1. Overview of the modeling process *2*
S1.2. Details of mechanistically resolved reactive transport model *3*
S1.3. Process importance analysis *12*
S1.4. Monte Carlo *12*
S1.5. Artificial neural network *13*
S1.6. Numerical model implementation and boundary conditions *14*
S1.7. Model calibration and parameterization *16*
S1.8. Data pre-treatment *17*
S2. Model validation 17
S2.1. Analytical validation of the kinetic sorption and equilibrium adsorption models *17*
S2.2. Testing of the model against field data and previous models *18*
S2.3. Validation of the use of artificial neural network *22*
S2.4. Validation of the modeling processes using mass budgets *22*
S2.5. Model assumptions and sensitivity of results to these *24*
S3. Calculation of ratios used in the profiles of Figure S16 and in the process importance analysis 25
S4. Detailed discussion on where and how sorption and geopolymerization control OC preservation 26
S4.2. Pathways of least-reactive DOC production *27*
S4.3. Burial is the start of preservation, not the end *27*
S5. Comparison with previous studies and directions for future research 28

S1. Details of model development

S1.1. Overview of the modeling process

The reactive transport model (RTM), machine learning, and Monte Carlo were employed to assess the importance of each underlying process in global preservation rates of DOC (or MOC formation rates) in marine sediment. The flowchart of the model deployment is illustrated in Fig. S3. Once the RTM was developed and the proposed processes were incorporated and validated, the RTM was used in a Monte Carlo procedure (Fig. S3, Stage 1) in which the model was run iteratively for 1000 to 2000 times using randomly varied input parameters to obtain output indicators (i.e., the preservation rates for different mineral-phase DOC (MOC) fractions). These inputs-outputs datasets were then fed into a previously developed artificial neural network (ANN) code[1] to assess the importance of each input parameter (Fig. S3, Stage 2). The role of different processes in MOC formation was determined based on the importance of their relevant model input parameters after grouping the parameters into six categories standing for six underlying processes, including DOC hydrolysis (cascaded breakdown of various DOC pools, main text Fig. 1), mixing (a transport mechanism related to bio-irrigation and bioturbation), DOC remineralization (transformation of DOC or GPS species to DIC), equilibrium adsorption, kinetic sorption, and geopolymerization as shown in Tables S1, S8, and S9. In doing so, the maximum importance of one of the parameters in each group of parameters was selected as the importance of that group of parameters. Alternatively, we summed up the importance of all parameters in each group and divided it by the number of parameters in that group to cancel the role of the parameter numbers in determining the importance of each process. We compared the final results based on this weighted average method with those obtained based on the maximum of each group and found they are similar, with a correlation coefficient of 0.926. Herein, we only report the results based on the maximum importance in each category instead of the weighted average of each category.

We obtain the ranges and statistical distributions from global grid data for six of the input parameters (including sediment accumulation rate, water depth, sediment surface porosity, and sediment-water interface concentrations of POC, O_2, and NO_3, Tables S1, S8, Figs. S1, S2) and by compiling data from 10 previous studies containing field data and modeling results. These studies and their data are summarized in Table S2. The statistics obtained for these data, e.g., lower and upper fences, are listed in Table S1 and were used for selecting the ranges for most of the other

unknown parameters. The ranges for new parameters were also either taken from the literature or from fitting the model to literature field data, as will be discussed later in Section S1.4.

S1.2. Details of mechanistically resolved reactive transport model

S1.2.1. Proposed conceptual model

Similar to the multi-G model[2], here we consider a series of POC pools that are hydrolyzed at different rates ranging from those of labile to least-reactive species as illustrated in main text Fig. 1. Unlike previous models of DOC (main text Fig. 1A)[3,4], however, in which each POC pool is hydrolyzed to a counterpart DOC pool, we assume that the hydrolysis of all POC pools results in a single DOC pool (DOC_1) which has the highest molecular weight (MW) and lowest lability in terms of the potential for direct remineralization to DIC among DOC pools (main text Fig. 1B). This DOC pool then breaks down to more labile DOC pools with lower MW (DOC_2 to DOC_m) in a cascaded manner similar to the recently proposed model for DOC hydrolysis in the ocean water column[5], while each of these DOC components can also be directly remineralized to dissolved inorganic carbon (DIC) with rates increasing from DOC_1 towards the last pool (DOC_m). Geopolymerized organic substances (i.e., GPS-DOC, hereafter simply GPS) start to form from DOC_m in an opposite sequential process as a result of condensation, i.e., in this process, MW increases and lability decreases from GPS_1 towards GPSp. The last component, GPSp, eventually contributes to the least-reactive DOC pool (lrDOC). Similar to DOC pools, all GPS pools can be directly remineralized to DIC but with reaction rates decreasing from GPS_1 towards GPS_p.

The DOC_1 pool is heterogeneous and in addition to its contribution to DOC_2, it contains a fraction of intrinsically undegradable DOC, which is freshly liberated from POC because it has labile bonds within the bulk POC but low intrinsic reactivity when released into the pore-water as colloidal or dissolved phases[6-8]. We, therefore, consider a pathway through which DOC_1 transfers directly to the lrDOC pool while DOC_1 also acts as a source for lower MW DOC and subsequent GPS components. Our model decouples DOC hydrolysis from DOC remineralization and assumes that while hydrolysis leads to more reminerlizable DOC and decreases MW, condensation reaction (geopolymerization) leads to less reminerlizable DOC and increased MW. Smallest MW DOC fractions are at the center of this cascade, giving rise to the potential preservation of this small-MW fraction over a long time due to other processes such as dilution in the water column[9,10] or sorption in sediment pore-waters[11-13]. It should also be noted that size-reactivity continuum

observations[6-8], where with increasingly lower MW, DOC displays lower reactivity, are unlikely to be due to the hydrolysis process. Recent research indicates that these observations can be explained by other processes, such as the dilution hypothesis[9,10], where lower MW and thus smaller molecules have higher diffusivity, and can thus dilute more easily in seawater and hence escape microbial degradation (see Eq. S21[14]). In this way, these molecules persist for longer in seawater, and it is observed that lower MW is correlated with lower reactivity and longer age. All DOC and GPS constituents in our model can interact with mineral surfaces through a two-site model for equilibrium adsorption and kinetic sorption/desorption. Although sorption in river sediments has been considered before[15,16], our model is the first to consider kinetic and equilibrium sorption together in a marine sediment early diagenesis model, and we also consider geopolymerization and sequential hydrolysis in this model for the first time. There might still be other processes that can play a role in OC preservation, e.g., sulfurization, which can render OC unreactive, but we have not considered them in the present study as they are mostly environment-specific[17].

S1.2.2. Mathematical model description and parametrization

A recently developed RTM[18] was modified to include DOC and GPS components and the processes of hydrolysis, equilibrium adsorption, kinetic sorption, and geopolymerization. The final model considers all common early diagenesis reactions for different compounds including dissolved species (O_2, SO_4, NH_4, NO_3, DIC, H_2S, CH_4, Fe^{2+}, Mn^{2+}, DOC_1 to DOC_m, GPS_1 to GPS_p, and lrDOC) and particulate species (highly reactive iron oxide ($Fe(OH)_3^{HR}$), moderately reactive iron oxide ($Fe(OH)_3^{MR}$), non-reactive iron oxide ($Fe(OH)_3^{UR}$), MnO_2, FeS, FeS_2, S^0, and POC_1 to POC_n) as listed in Table S10. We also considered similar reactions for mineral organic carbon (MOC) pools which are formed by kinetic sorption of DOC or GPS to mineral phases through occlusion into, or co-precipitation with mineral phases (see also Section S1.7). For the sake of simplicity, however, and reducing the overall number of parameters, we assumed that MOC species are non-reactive. Although our RTM code could consider a variable number of POC, DOC and GPS components, in order to gain a reasonable model runtime and to reduce the number of parameters, herein, we considered seven species for POC, four species for DOC and two species for GPS in addition to lrDOC. The selected number of species is not expected to make a noticeable impact on the model results as we investigated the effect of increasing these numbers on the model outputs. In doing so, we used the model at the step where DOC species were just added similar to the model of Burdige et al.[4,19] (Step 1, Table S6). We increased the number of DOC species from

3 to 9 by replacing the third DOC species, DOC_3, as an example, with 7 new DOC species. In this way, we divided the degradation rate of DOC_3, i.e., k_{DOC3}, by 7 (k_{DOC3} /7) and assigned this value to this new DOC species and the 6 other new species. used this for the new DOC_3 species and the 6 other new DOC species. We also divided the hydrolysis rate of DOC_3 by 7 and used this for the new DOC_3 species and the 6 other new DOC species. The result shown in Fig. S17 and S18 demonstrate that the addition of multiple new DOC species does not make a discernible change to the concentration profile of any other species considered in the model. Especially, the concentration profiles of newly added sub-pools are the same as the original DOC_3 pool since different pools operate serially[5,20-22] and we specified the same degradation/hydrolysis rates for different subpools by dividing the rate of the original pool. Therefore, in framing the conceptual model, rather than the number of species considered, the important point mainly is consistency with previous literature[23] and consideration of other modeling aspects, e.g., consistency with the conceptual model to make the most biogeochemical sense, smooth transition of rates across different species for the convenience of the numerical solution and minimizing the number of unknown model parameters. It should be mentioned that here to compare the number of pools considered in our model with a larger potential number of pools, we used a similar parameterization for the two cases so that we did not need to fit the model to field data again for the new parameterization as that could make the comparison of the model performance for different numbers of pools complex.

It should be noted that in our model, MOC is different from POC by origin, i.e., POC originates from the water column whilst MOC is formed in the sediments via kinetic sorption of DOC to minerals. Current field measurements are mostly based on total OC, which is equal to POC+MOC in our model, although some techniques such as bond-strength characterization may be useful approaches to distinguish MOC from POC in field measurements and thus improve our understanding of different classes of OC in nature[11].

Considerations of equilibrium adsorption, kinetic sorption, hydrolysis and geopolymerization are described below, and the list of all other reactions in the model is given in Table S10.

S1.2.3. Incorporation of equilibrium adsorption and kinetic sorption

We considered a two-site equilibrium-kinetic model[24,25] for (i) equilibrium adsorption of DOC onto mineral surfaces and (ii) kinetic retention/dissociation of DOC within the mineral matrix.

Despite the frequent use of a linear equilibrium adsorption expression in early diagenesis models[26] and riverine sediments[39,40], developing a kinetic expression for interacting dissolved species with minerals in the context of marine early diagenesis has not been considered to date. One complexity lies in tracking the kinetically associated dissolved species with the mineral phase, which itself undergoes advection (burial), bioturbation and reaction. We borrowed the basic concept of colloid-mediated transport of sorbing contaminants from the context of groundwater modeling[27] to develop a kinetic forward-backwards mass transfer model in marine sediment where the minerals are also transported in addition to dissolved species.

In porous media systems where both dissolved and particulate phases are transported, the kinetic sorption may be accounted for by considering six types of concentrations. These include the concentration of each dissolved species in the aqueous phase (pore water), C_d, the concentration of each dissolved species in the solid phase of porous media, S_d, the concentration of each particulate species in the aqueous phase, C_p, the concentration of each particulate species in the solid phase of porous media, S_p, the concentration of each dissolved species sorbed to particles in the aqueous phase, C_{ap}, and the concentration of each dissolved species sorbed to particles in the solid phase, S_{ap}. For marine sediments, one can assume that the particulate phase is attached to the porous media, which itself is transporting, and thus C_p can be merged with C_{ap} and consequently S_p can be combined with C_p too. Thus, the six types of concentrations are reduced to three for marine sediment porous media: C_d, C_p and S_d. This means that three sets of governing equations are required to model kinetic sorption at the kinetic sites as follows:

1. The governing equation for dissolved species:

$$\varphi \frac{\partial C_{d_i}}{\partial \mathrm{t}} = \frac{\partial}{\partial z}\left(\varphi D \frac{\partial C_{d_i}}{\partial z}\right) - \frac{\partial\left(\varphi v_d C_{d_i}\right)}{\partial z} + \varphi\alpha\left(C_{d_i}(0) - C_{d_i}(Z)\right) + \varphi \sum_{j=1} R_{d_{i,j}} + \varphi R_{EqAds} - \varphi\, k_{sorpfr_i} C_{d_i} + \varepsilon\rho_s k_{sorpb_i} S_{d_i} \tag{S1}$$

2. The governing equation for particulate species:

$$\rho_s \varepsilon \frac{\partial C_{p_i}}{\partial \mathrm{t}} = \rho_s \frac{\partial}{\partial z}\left(\varepsilon D_b \frac{\partial C_{p_i}}{\partial z}\right) - \rho_s \frac{\partial}{\partial z}\left(\varepsilon v_p C_{p_i}\right) + \rho_s \varepsilon R_{p_i} \tag{S2}$$

3. The governing equation for mineral phase OC resulting from the kinetically sorbed fraction of dissolved species:

$$\rho_s \varepsilon \frac{\partial S_{d_i}}{\partial t} = \rho_s \frac{\partial}{\partial z}\left(\varepsilon D_b \frac{\partial S_{d_i}}{\partial z}\right) - \rho_s \frac{\partial}{\partial z}\left(\varepsilon v_p S_{d_i}\right) + \rho_s \varepsilon \sum_{j=1} R_{Sd_{i,j}} + \varphi\, k_{sorpf_i} C_{d_i} - \rho_s \varepsilon k_{sorpb_i} S_{d_i} \quad \text{(S3)}$$

Besides, to account for the equilibrium adsorption of dissolved species, another set of equations may be required, which is given below, although this can be combined with the above equations as will be described later:

$$\rho_s \varepsilon \frac{\partial Se_{d_i}}{\partial t} = \rho_s \frac{\partial}{\partial z}\left(\varepsilon D_b \frac{\partial Se_{d_i}}{\partial z}\right) - \rho_s \frac{\partial\left(\varepsilon v_p Se_{d_i}\right)}{\partial z} + \rho_s \varepsilon \sum_{j=1} R_{EqSd_{i,j}} + \rho_s \varepsilon R_{S\,EqAds} \quad \text{(S4)}$$

where C_{di} is the concentration of dissolved species i (mM or μmol.cm^{-3} of pore water), C_{pi} is the concentration of particulate species i (g.g^{-1}), S_{di} is the concentration of dissolved species i kinetically sorbed to sediment minerals (μmol.g^{-1} of solid sediments), φ is porosity, ε is the solid fraction of sediments which is equal to $1-\varphi$, v_d and v_p are the burial velocities of pore-water and particulate species (cm.yr^{-1}), ρ_s is the dry density of sediments (g.cm^{-3}), D_i is the apparent diffusion coefficient of dissolved species i (cm^2.yr^{-1}), α is the bio-irrigation coefficient (cm^2.yr^{-1}), D_b is the bioturbation coefficient (cm^2.yr^{-1}), z is the sediment depth with respect to the coordinate system located at the sediment-water interface (cm), R_p, R_d, Rs_d, and $R_{Eq}s_d$ stand for reaction rates of particulate, dissolved, kinetically sorbed, and adsorbed species (yr^{-1}, μmol.cm^{-3}.yr^{-1}, μmol.g^{-1}.yr^{-1}, and μmol.g^{-1}.yr^{-1}), respectively, which are temporally and spatially variable, k_{sorpf} is the forward kinetic sorption rate coefficient (yr^{-1}), and k_{sorpb} is the backward kinetic desorption rate coefficient (yr^{-1}). R_{Eqads} and R_{sEqads} are exchange rates for dissolved and adsorbed (equilibrium) phase equations (μmol.cm^{-3}.yr^{-1} and μmol.g^{-1}.yr^{-1}, respectively). The unit of every term in Eqs. (S1) and (S3) is μmol cm^{-3}.yr^{-1} and in Eq. (S2) is gr.cm^{-3}.yr^{-1}.

Considering the linear isotherm model, $Se_{d_i} = K_{d_i} C_{d_i}$, where K_{d_i} is the distribution or partitioning coefficient (cm^3.g^{-1}), Eq. (S4) is then rewritten as[26,28]:

$$\rho_s \varepsilon K_{d_i} \frac{\partial C_{d_i}}{\partial t} = \rho_s K_{d_i} \frac{\partial}{\partial z}\left(\varepsilon D_b \frac{\partial C_{d_i}}{\partial z}\right) - \rho_s K_{d_i} \frac{\partial\left(\varepsilon v_p C_{d_i}\right)}{\partial z} + \rho_s \varepsilon \sum_{j=1} R_{EqSd_{i,j}} + \rho_s \varepsilon R_{S\,EqAds} \quad \text{(S5)}$$

The following mass balance expression may be used to combine Eqs. (S1) and (S5), Ref.[28]:

$$R_{S\,EqAds} = \frac{-\varphi}{\varepsilon \rho_s} R_{EqAds} \quad \text{(S6)}$$

Since the equilibrium adsorbed phase is in fast exchange with the dissolved phase, we assume that no reaction is considered for the equilibrium adsorbed phase ($\sum_{j=1} R_{EqSd_{i,j}} = 0$). Substituting Eqs. (S1) and (S5) into Eq. (S6), the governing equation for dissolved species is:

$$\begin{aligned}\varphi \frac{\partial C_{d_i}}{\partial \mathrm{t}} + \rho_s \varepsilon K_{d_i} \frac{\partial C_{d_i}}{\partial t} &= \frac{\partial}{\partial z}\left(\varphi D \frac{\partial C_{d_i}}{\partial z}\right) + \rho_s K_{d_i} \frac{\partial}{\partial z}\left(\varepsilon D_b \frac{\partial C_{d_i}}{\partial z}\right) - \frac{\partial\left(\varphi v_d C_{d_i}\right)}{\partial z} \\ &- \rho_s K_{d_i} \frac{\partial\left(\varepsilon v_p C_{d_i}\right)}{\partial z} + \varphi\alpha\left(C_{d_i}(0) - C_{d_i}(Z)\right) + \varphi \sum_{j=1} R_{d_{i,j}} \\ &- \varphi\, k_{sorpf_i} C_{d_i} + \varepsilon \rho_s k_{sorpb_i} S_{d_i}\end{aligned} \tag{S7}$$

which can be rearranged as:

$$\begin{aligned}\left(\varphi + \rho_s \varepsilon K_{d_i}\right) \frac{\partial C_{d_i}}{\partial t} &= \frac{\partial}{\partial z}\left(\left(\varphi D + \rho_s \varepsilon K_{d_i} D_b\right) \frac{\partial C_{d_i}}{\partial z}\right) - \frac{\partial}{\partial z}\left(\left(\varphi v_d + \rho_s \varepsilon K_{d_i} v_p\right) C_{d_i}\right) \\ &+ \varphi\alpha\left(C_{d_i}(0) - C_{d_i}(Z)\right) + \varphi \sum_{j=1} R_{d_{i,j}} - \varphi\, k_{sorpf_i} C_{d_i} \\ &+ \varepsilon \rho_s k_{sorpb_i} S_{d_i}\end{aligned} \tag{S8}$$

or can be written in an expanded form as:

$$\begin{aligned}\left(\varphi + \rho_s \varepsilon K_{d_i}\right) \frac{\partial C_{d_i}}{\partial t} &= \varphi D \frac{\partial}{\partial z}\left(\frac{\partial C_{d_i}}{\partial z}\right) + \frac{\partial}{\partial z}(\varphi D) \frac{\partial C_{d_i}}{\partial z} \\ &+ \rho_s K_{d_i}\left[\varepsilon D_b \frac{\partial}{\partial z}\left(\frac{\partial C_{d_i}}{\partial z}\right) + \frac{\partial}{\partial z}(\varepsilon D_b) \frac{\partial C_{d_i}}{\partial z}\right] \\ &- \left[\frac{\partial}{\partial z}(\varphi v_d) C_{d_i}\right) + \varphi v_d \frac{\partial C_{d_i}}{\partial z} + \rho_s K_{d_i}\left(\frac{\partial}{\partial z}(\varepsilon v_p) C_{d_i} + \varepsilon v_p \frac{\partial C_{d_i}}{\partial z}\right)\right] \\ &+ \varphi\alpha\left(C_{d_i}(0) - C_{d_i}(Z)\right) + \varphi \sum_{j=1} R_{d_{i,j}} - \varphi\, k_{sorpf_i} C_{d_i} \\ &+ \varepsilon \rho_s k_{sorpb_i} S_{d_i}\end{aligned} \tag{S9}$$

The kinetic sorption rate (μmol.cm^{-3}.yr^{-1}) based on Eq. (S3) is:

$$R_{sorp_{d_i}} = \varphi k_{sorpf_i} C_{d_i} \tag{S10}$$

The desorption rate (μmol.cm^{-3}.yr^{-1}) in Eq. (S3) is:

$$R_{des_{d_i}} = \varepsilon \rho_s \, k_{sorpb_i} S_{d_i} \tag{S11}$$

In the above formulation for kinetic sorption, we used k_{sorpf} and k_{sorpb} (yr^{-1}), which are dependent on porosity and, therefore, the depth of sediments. These can be related to constant parameters as follows:

$$k_{sorp_i} = \varphi k_{sorpf_i} \tag{S12}$$

$$Kd_{sorp_i} = \frac{\varphi k_{sorpf_i}}{\varepsilon \rho_s \, K_{sorpb_i}} \tag{S13}$$

where k_{sorp} is the mass transfer rate between the dissolved and kinetically sorbed phases to minerals (MOC pools) (yr^{-1}) and Kd_{sorp} is the so-called distribution coefficient in the kinetic mass transfer expression (cm^3.g^{-1}). Accordingly, Eqs. (S3) and (S8) can be rewritten as:

$$\rho_s \varepsilon \frac{\partial S_{d_i}}{\partial t} = \rho_s \frac{\partial}{\partial z}\left(\varepsilon D_b \frac{\partial S_{d_i}}{\partial z}\right) - \rho_s \frac{\partial}{\partial z}\left(\varepsilon v_p S_{d_i}\right) + \rho_s \varepsilon \sum_{j=1} R_{Sd_{i,j}} + k_{sorp_i} C_{d_i} - \frac{k_{sorp_i}}{Kd_{sorp_i}} S_{d_i} \tag{S14}$$

$$\begin{aligned}\left(\varphi + \rho_s \varepsilon K_{d_i}\right) \frac{\partial C_{d_i}}{\partial t} &= \frac{\partial}{\partial z}\left(\left(\varphi D + \rho_s \varepsilon K_{d_i} D_b\right) \frac{\partial C_{d_i}}{\partial z}\right) - \frac{\partial}{\partial z}\left(\left(\varphi v_d + \rho_s \varepsilon K_{d_i} v_p\right) C_{d_i}\right) \\ &\quad + \varphi \alpha \left(C_{d_i}(0) - C_{d_i}(Z)\right) + \varphi \sum_{j=1} R_{d_{i,j}} - k_{sorp_i} C_{d_i} + \frac{k_{sorp_i}}{Kd_{sorp_i}} S_{d_i}\end{aligned} \tag{S15}$$

At very high k_{sorp} values, the kinetic mass-transfer model should converge to an equilibrium model similar to the one we have used to describe the adsorption phenomenon[24,29,30]. We use this notion to validate the mathematical and numerical model developments for kinetic sorption and equilibrium adsorption.

S1.2.4. Hydrolysis and geopolymerization model description

Hydrolysis is a stage of degradation where organic molecules are liberated from the bulk of POC and further undergo breakdown into smaller molecules in a sequential process[31]. We consider the first stage of the hydrolysis similar to the conventional first-order multi-G degradation model[2] with a series of POC pools converting to a single DOC pool, DOC_1, in parallel:

$$\left\{\frac{\partial C_{p_i}}{\partial \mathrm{t}}\right\}_{Hydrolysis} = R_{p_i} = k_i C_{p_i} \tag{S16}$$

where k_i is the hydrolysis rate constant, which was considered in a similar way to the degradation rate constants of POC in the continuum model following previous studies[18,32-34]. In the continuum model, a constant set of k_i values is assumed with ranges that may cover those happening in nature (here assumed to be 10^{-8} to 10^{2} yr^{-1}). The POC arriving at the sediment surface is fractioned into a number of components corresponding to the number of k_i values, and either the concentration or rain rate of each fraction is determined based on a gamma distribution[34]. Here we first considered 14 classes with k_i and fraction values following Ref.[18], and then we selected every other class to match the number of POC species, which is selected as seven species in the present study. We then scale the fractions by the sum of the fractions of 14 classes, which is one, divided by the sum of the fractions of the maintained seven classes so that the sum of the fractions of the seven classes after scaling also becomes one. It should be noted that in the continuum approach, the number of classes does not affect the results as the fractions are sampled from a gamma distribution[33,34]. The continuum model introduces only two constant parameters related to the gamma distribution, including "a_{Gam}" which represents the average lifetime of more reactive components of the POC spectrum (yr) and "v_{Gam}" which is related to the shape of the gamma distribution (dimensionless)
The sequential stage of the hydrolysis may be modeled using a consecutive (chain-reaction) first-order reaction expression[5,22]:

$$\left\{\varphi\frac{\partial C_{d_i}}{\partial \mathrm{t}}\right\}_{Hydrolysis} = \left\{\varphi\sum_{j=1} R_{d_{i,j}}\right\}_{Hydrolysis} = \lambda_{DOCi-1} C_{d_{i-1}} - \lambda_{DOCi} C_{d_i} \tag{S17}$$

where λ_{DOCi} is the conversion rate of DOC_i to DOC_{i+1}, and λ_{DOCi-1} is the conversion rate of DOC_{i-1} to DOC_i in yr^{-1}.
The same mathematical formula is assumed to describe geopolymerization[20,21]:

$$\left\{\varphi\frac{\partial C_{d_i}}{\partial \mathrm{t}}\right\}_{Geopolymerisation} = \left\{\varphi\sum_{j=1} R_{d_{i,j}}\right\}_{Geopolymerisation} = \lambda_{GPSi-1}C_{d_{i-1}} - \lambda_{GPSi}C_{d_i} \quad \text{(S18)}$$

where λ_{GPSi} is the conversion rate of GPS_i to GPS_{i+1}, and λ_{GPSi-1} is the conversion rate of GPS_{i-1} to GPS_i in yr^{-1}. The influence of molecular weight variations across DOC and GPS pools (main text Fig. 1) is considered through the diffusion coefficients of these species and the influence of variations in lability across DOC and GPS pools is reflected in the thresholds of remineralization rates and conversion rates (λ values) of these components (Table S1).

As described in the Proposed Conceptual Model Section in the main text (see also main text Fig. 1), we consider an additional pathway for the transfer between the first DOC pool (DOC_1) and lrDOC pool which is also comprised of the last GPS pool (GPS_P). This results in the following equations for the hydrolysis and/or geopolymerization reactions of the first DOC pool and lrDOC pools, respectively:

$$\left\{\varphi\frac{\partial C_{d_1}}{\partial \mathrm{t}}\right\}_{Hydrolysis} = \left\{\varphi\sum_{j=1} R_{d_{1,j}}\right\}_{Hydrolysis} = 10^6\rho_s\frac{\varepsilon}{\varphi.AW_{OC}}\sum_{j=1} R_{p_{1,j}} - \lambda_{DOC1}C_{d_1} - \lambda_{DOC1r}C_{d_1} \quad \text{(S19)}$$

$$\left\{\varphi\frac{\partial C_{d_r}}{\partial \mathrm{t}}\right\}_{Hydrolysis} = \left\{\varphi\sum_{j=1} R_{d_{r,j}}\right\}_{Hydroysis} = \lambda_{GPSp-1}C_{d_{r-1}} + \lambda_{DOC1r}C_{d_1} \quad \text{(S20)}$$

where AW_{OC} is the molar mass of carbon (assumed 12 $g.mol^{-1}$), λ_{GPSp-1} is the conversion rate of GPS_{p-1} to lrDOC, and λ_{DOC1r} is the conversion rate of DOC_1 to lrDOC, index r represents the most refractory pool among DOC and GPS pools ($r = m+p$, where m is the number of DOC pools and p is the number of GPS pools which is merged with lrDOC, main text Fig. 1).

We assume that the decrease and increase of molecular weight (MW) across DOC and GPS pools, respectively, follow a linear trend with threshold values assumed to be in the range of 10^4 Da down to 50 Da for DOC_1 to DOC_m, and from >50 Da (c.a. 3367 Da when two GPS pools are considered) to 10^4 for GPS_1 to lrDOC[4,20,35,36]. Accordingly, in our current modeling, we used MWs of 10000, 6683.3, 3366.7, 50, 3366.7, 6683.3, and 10000 Da for DOC_1, DOC_2, DOC_3, DOC_4, GPS_1, GPS_2 and lrDOC, respectively.

Diffusion coefficients of DOC species were calculated based on the Stokes-Einstein equation following[14]:

$$D_{DOCi} = \frac{R.\mathrm{T}}{6\pi.N.\eta}.\sqrt[3]{\frac{4\pi.\rho_{\mathrm{DOC}}.N}{3MW_{DOCi}}} \tag{S21}$$

where D_{DOCi} is the diffusion coefficient of DOC (or GPS) species, i (cm^2yr^{-1}), T is absolute temperature (K), R is the universal gas constant, N is Avagadro's number, MW_{DOCi} is the molecular weight of DOC_i (or GPS_i), η is the dynamic viscosity of the medium calculated following Refs.[37,38], ρ_{DOC} is the density of DOC (or GPS) which is assumed the same as the density of typical biomolecules, 1.5 $g.cm^{-3}$ Refs.[14,39].

S1.3. Process importance analysis

Although global process importance analysis methods such as Morris (derivative-based) and Sobol (variance-based) have been used for Earth and environmental models[40-42], for complex models with a large number of parameters such as the RTM developed in the present study, the use of such process importance analysis methods can be computationally expensive[43,44] and/or might suffer from the scale issue, which is the loss of small-scale variations in the response surface when the step size of the analysis is taken relatively large[45,46]. A more efficient approach may be to use RTM along with Monte Carlo and artificial intelligence techniques to obtain the global importance of influential factors[1,43,44,46,47]. In this way, we run the RTM iteratively, e.g., for 1000 times, using random input parameter values and employ ANN to fit the resultant input-output datasets of the iterative model runs to create a meta-model, thereby obtaining the global importance of different factors[43,44,46,47]. Relative importance of the categorized processes in controlling PE and MOC formation at 1 m depth is shown in main text Fig. 3. Details of Monte Carlo and ANN procedures are described in the following sections.

S1.4. Monte Carlo

The Monte Carlo approach was applied by random sampling of the input parameters of the RTM over their global ranges and statistical distributions taken from six global grid datasets (with a resolution of 1°×1°, Figs. S1, S2) and based on statistical analyses of 10 selected field-modeling datasets from previous studies[4,18,19,48-55]. The global grid datasets include water depth[56], sediment accumulation rate[57], porosity[58], and sediment-water interface concentrations of POC, which are

assumed to be the same as total OC[58], NO_3 Refs.[59,60], and O_2 Refs.[59,60]. The ranges, probability distribution assumptions and other statistics (upper and lower fences and median) selected for the data are given in Tables S1, S8. The ranges of parameter values for the new parameters introduced in the present study were assumed to be from near zero values to five times the value obtained in fitting the model to field data[4,19], or they were assumed based on other reasonable boundary values, e.g., the values of the same parameter from other components of DOC or GPS species (Tables S1, S8). We use a broad range of parameter values (e.g., by selecting the upper and lower fences of the data available, Supplementary Table S1) because this leads to a more robust understanding of their role in OC preservation, as long as the uncertainties are reasonable, whilst the Monte Carlo approach assures that the number and the ranges of model parameters do not affect the insights obtained in our study.

Different probability distributions were fitted to the six global grid datasets and the final distributions used in the Monte Carlo random sampling process were selected based on a trade-off between goodness-of-fit for histogram plots and simplicity. The selected distributions fitted to field data histograms, along with their means (μ) and standard deviations (σ), are shown in Fig. S8. A normal distribution appeared to be appropriate for these parameters except for POC_0, which fits better with a log-normal distribution (Fig. S2). For all other parameters, we used a uniform distribution except for 'a_{Gam}' parameter of the gamma distribution used in the continuum model for POC hydrolysis. We assumed that a_{Gam} was log-normally distributed. The log-normal distribution for a_{Gam} was assumed because considering a previous empirical equation[34] relating a_{Gam} to the sediment accumulation rate and using random data generated for the sediment accumulation rate, we found that the distribution of a_{Gam} is log-normal. The final selected types of distributions for all parameters are listed in Table S8. The uncertainties are estimated by determining the confidence intervals (CI) based on a 95% confidence level according to the 2.5th and 97.5th percentiles of the Student's t-distribution[61].

S1.5. Artificial neural network

The artificial neural network (ANN) is a versatile and universal tool for function approximation problems and is notable for its application to complex, non-linear systems[1,62]. The commonly-used ANN structure is a three-layer configuration comprising input, hidden and output layers[1,63-65]. Each of these layers is composed of a series of nodes (neurons) with their numbers in the input and output layers corresponding to the number of input and output variables, respectively. The number

of neurons on the hidden or middle layers should be optimized when finding the best fit during the training process[63,66]. A summation of signals (information) from all nodes in the input layer is propagated to each node in the hidden layer and likewise from all nodes of the hidden layer to each node in the output layer using methods such as the back-propagation algorithm[63]. While this summation is based on a simple algebraic linear relationship, there are different types of additional transfer (or activation) functions that are exerted on the input signals into nodes of each layer[1,63].

To find insight into the relationships between inputs and outputs of ANN, several process importance analysis approaches have been developed, among which the Partial Derivatives (PaD) method has been identified as the most useful and stable approach[67]. The PaD method is based on taking the partial derivative of the output variables with respect to the input variables. In this process, in addition to the derivative of the main connections between different layers, the derivatives of the transfer functions used for processing the signals are also taken into account[1,63-65].

In the present study, we considered a simple three-layer configuration with a hyperbolic tangent sigmoid function (tansig function of MATLAB) as the transfer function for the hidden layer and a linear transfer function (purelin function of MATLAB) for the output layer. The ANN training was performed using the Levenberg–Marquardt back-propagation learning algorithm for optimization of ANN indigenous parameters (hyper-parameters) known as weights and biases based on the cross-validation method (i.e., keeping randomly-selected 15% of the input-output dataset for validation and 15% for testing while the rest of the dataset (70%) is used for training ANN)[63,64,68]. The optimum number of hidden layer nodes was obtained by iterating the training process over a range of 4 to 36 nodes in each run of the Monte Carlo process. The MATLAB code performing ANN modeling and process importance analysis was adapted from Babakhani et al.[1] where it was used for modeling nanoparticle transport in porous media. Here, we further excluded 15% of the original dataset from the analysis for separate testing of the ANN prediction ability.

S1.6. Numerical model implementation and boundary conditions

The RTM was developed in Mathematica (version 11.3) with a similar basis to that developed by Dale et al.[18] but with a reconsideration of manganese and iron species, e.g., considering three reactive (oxyhydr)oxide iron pools instead of four pools in Ref.[18] and with a modification of the numerical approach. To speed up the computations, the code was modified to conduct the space discretization manually instead of the previously-used automatic approach built into Mathematica.

Here we used a third-order central-in-space finite difference scheme for the discretization of terms, including space (depth) variable derivatives. This scheme was selected because it showed the best trade-off between accuracy and computational speed compared to first, second or higher-order schemes. The integration of the terms with time derivatives was performed using the NDSolveValue function of Mathematica. The spatial discretization of the model was conducted using a high-resolution stepping at the top of the sediments that was decreasing based on a third-order polynomial function. The third-order function appeared to perform better (in terms of numerical stability and computational speed) than a second-order stepping function, especially if the depth of the simulated sediment was relatively large.

The model was run using a long simulation time (five times the expected simulation time calculated based on the depth divided by the burial rate) in order to ensure that the steady-state condition was reached when the model outputs were recorded. The mass balance for each species was checked by comparing depth-integrated reaction rates and fluxes across the model domain boundaries[18]. A tolerance of 1% was assumed for the mean mass balance errors across all species, i.e., those model runs which did not meet this criterion were automatically run with a finer grid step to yield a lower mass balance error or if they still could not meet the criterion, they were excluded from the results in Monte Carlo simulations.

Upper boundary conditions were considered as the first type (constant concentration or Dirichlet type) for all dissolved species[4,19], and a third type (Robin) boundary condition was assumed for other solid species as well as kinetically sorbed species[18]. This flux ($\mu mol.cm^{-2}.yr^{-1}$) for kinetically sorbed species, as an example, is as follows:

$$J_{S_{d_i}} = \rho_s \varepsilon \left(v_p S_{d_i} - D_b \frac{\partial S_{d_i}}{\partial z}\right) \tag{S22}$$

For POC species, here we utilized the constant-concentration boundary data instead of the more commonly used flux-type boundary data since more reliable datasets are available for sediment-water interface concentration of POC rather than POC flux[58]. This constant concentration POC values were converted to flux boundary condition by calculating mass accumulation rate (MAR) following[69]. The lower boundary conditions were considered as the second type (zero flux or Neumann type) boundary condition for all species.

The Monte Carlo process with RTM (using Mathematica) was executed on a 2.9 GHz Core™ i9 CPU with 32 GB RAM, while the Monte Carlo process for ANN process importance analysis

(using MATLAB) was conducted on High-Performance Computing clusters (ARC4) at the University of Leeds, possessing 149 nodes with 40 cores and 192 GB of memory each and an SSD within the node with 170 GB of storage.

S1.7. Model calibration and parameterization

In the validation process of the RTM, where the model was fitted against field data, the unknown model parameters were calibrated both automatically (inverse model) and manually. A simple optimization algorithm previously described and coded in MATLAB[70] was coded in Mathematica in the present study. In cases where this approach did not seem to be efficient enough or was unlikely to find the global optimum of the objective function, we further used basic manual calibration to improve the model fits to the data.

To optimize the model parameters of the RTM, it is necessary to use a global objective function that can synchronize the outputs for different species involved in the modeling because the output concentration of every species can be on a different order of magnitude than others and thus contribute differently to the overall objective function of all species. One way might be to get the sum of normalized output values as a consistent single objective function. On the other hand, it is challenging to synchronize different output concentrations by normalization of each output by its initial/boundary values because the initial and/or boundary conditions of some of the species can be zero, e.g., DOC components are all produced within the model domain during the simulation period. Hence, we herein directly used the Nash-Sutcliffe model efficiency (NSE) criterion[71] as the objective function, which was maximized during the calibration process to make it closer to one. As NSE has a consistent range ($-\infty$ to 1) for concentrations of all species fitted to their corresponding field datasets, and can thus be averaged for all species, this approach could properly deal with the challenge of the different concentration ranges of various components that can contribute to the objective function with different weights. The NSE is calculated as follows:

$$NSE = 1 - \frac{\sum_{j=1}^{n_{obs}} (X_j^o - X_j^m)^2}{\sum_{j=1}^{n_{obs}} (X_j^o - \overline{X^o})^2} \qquad \text{(S23)}$$

where X_j^o is the jth record of the quantity being measured or observed, n_{obs} is the total number of measured records or observations, X_j^m is the jth record of the quantity being modeled or computed, $\overline{X^o}$ is the average of the observation data. NSE values above 0.5 are generally acceptable, with values close to 1 indicating better matches[64].

The list of all model parameters and whether they are considered variable or fixed based on the data from literature or global grid maps are given in Tables S1 and S8.

S1.8. Data pre-treatment

Certain parameter combinations could result in unreasonable model results and, thus, the existence of outliers in the generated dataset at the Monte Carlo stage (Stage 1, Fig. S3). We removed the outliers for all cases, which led to the loss of data by 0%, 10.1%, 10.5% and 10.7% for *PE*, preservation rates of (semi)labile DOC, GPS and lrDOC, respectively, compared to the entire dataset. It should be mentioned that at this stage, the model was initially run 2000 times. Nevertheless, since not all model runs were successful due to facing numerically difficult conditions, the final data obtained at this stage were comprised of 1756 cases. Furthermore, there were cases in different model runs across the random input parameter values where the model-produced bottom-water concentration of DIC yielded values less than the constant concentration bottom-water boundary condition specified for DIC at the sediment-water interface (which was 2.3 mM). Since the diffusion for dissolved species occurs from areas with greater concentrations towards areas with lower concentrations according to Fick's law[72], in those cases where the produced DIC inside the sediments was smaller than the sediment-water interface DIC concentration, selected as a constant value (2.3 mM) in all model runs, then DIC fluxed inward into rather than outwards from the sediments. This resulted in an opposite direction for the flux of DIC (influx), which was not physically justifiable. Therefore, we removed all these cases from the entire dataset, reducing the final number of data from 1756 to 1450 before removing the outliers described above. Even with the highest portion of outlier removal (10.7%), still, the size of the dataset was larger than 1000 records.

For the outlier removal, we used "rmoutliers" function of MATLAB to remove the outliers based on the default method of "median", which considers three scaled median absolute deviations. All data were log-transformed prior to feeding to the ANN (Stage 2 of the modeling algorithm).

S2. Model validation

S2.1. Analytical validation of the kinetic sorption and equilibrium adsorption models

At a high mass transfer rate where the kinetic condition is expected to reach an equilibrium state, the model outputs from the kinetic sorption model developed in the present study should be

comparable with those of the equilibrium adsorption model[24,29,30]. In this approach, by forcing the model parameters, we first turn off the equilibrium adsorption expression and run the model when the kinetic sorption expression is operating at high exchange rates, and get the model output concentrations versus depth profiles for all species. Then we run the model by turning off the kinetic sorption expression and operating the equilibrium adsorption expression and get the model outputs. Since the equilibrium expression has previously been used frequently in the literature of early diagenesis modeling, matching the two model outputs validates our formulation for the kinetic model. In this section, the model parameters and characteristics were selected mostly from Ref.[18], and adsorption coefficients were assumed to be similar to Ref.[55].

Results shown in Fig. S4 demonstrate that output concentrations for all particulate, dissolved and adsorbed species of the kinetic model at a high mass transfer rate match very well (R^2 = 1.000) with the output concentrations of the equilibrium model, verifying our approach towards development and implementation of a kinetic expression for interaction between dissolved species and minerals in early diagenesis models of marine sediments. Such a model had previously been considered through a derivation approach known as "separation of the kinetically influenced term method (SKIT)"[73], where the two governing equations for dissolved and solid phases were combined. The resulting single partial differential equation is one order higher, however, which is complex and computationally difficult to solve. In the present study's model, although the equations are not combined and are solved together numerically, the order and complexity of developed partial differential equations remain similar to those previously used in early diagenesis modeling.

S2.2. Testing of the model against field data and previous models

Our model was further tested against various datasets from previous studies[4,18,50,54] as described below:

1. The basic model, i.e., without DOC species terms but with the reconsideration of manganese and iron species compared to the previous model[18] and with a different numerical scheme, was matched against the model outputs of Meysman et al.[54] using the same modeling features and parameter values as those of Meysman et al. who used the MEDIA (Modeling Early DIAgenesis) software package for the data from Santa Barbara Basin sediment. The results of the model fit to the model outputs of Meysman et al.[54] are shown in Fig. S5. These results show excellent matches between the two models for all relevant species shown.

2. The model was fitted to the field data of Kraal et al.[50] by tuning some of the parameters but without modifying the characteristics of the basic model of Dale et al.[18], to see if better goodness-of-fit criteria can be obtained compared to the model of Ref.[50]. Parameter values of Ref.[50] with calibrated values in the current modeling are listed in Table S3. At this stage, we also included DOC but with a simplifying approach rather than considering the full cascaded hydrolysis processes of DOC presented in our conceptual model shown in main text Fig. 1. Here, we also aimed to test the application of NSE as an objective function in the model calibration process that is described in Section S1.7.

Concentration profiles of the model fitted with and without a basic consideration of DOC compared to the data of Kraal et al.[50] are shown in Figs. S6 and S8, respectively. The goodness-of-fit criteria are given in Table S4. The results show that the model is able to fit all species profiles except the FeS_2 profile and the decaying part of the Mn^{2+} profile. Similar to the above, this is attributed to the lack of carbonate species in our model. The goodness-of-fit criteria (R^2 and NSE), presented in Tables S4 and S5, demonstrate that, on average, the performance of the present model without DOC (mean $R^2 = 0.762$) shows improvement over the model of Kraal et al. (mean $R^2 = 0.724$) and is improved even further when DOC is included (mean $R^2 = 0.764$).

Here we also used two alternative approaches to consider the objective function in the automatic calibration process, i.e., the use of either typical determination coefficient (R^2) or NSE. The result of this investigation performed for the case without DOC shows that the use of NSE as an objective function substantially improves the final NSE values (mean NSE increases from -5.08 to 0.332, Tables S4 and S5) and shows a better visual match between the modeled and observation curves (Fig. S6 versus Fig. S7).

3. The RTM with DOC and GPS species and with all processes of adsorption, kinetic sorption, geopolymerization and hydrolysis included step-by-step was tested against the field and modeling data of Burdige et al.[4,19]. In step 1, we used all model characteristics and parameter values of Burdige et al.[4,19]. They used two models separately: OMSN (Organic Matter/Sulfate/Nitrogen) model and the DOC model to fit the field data, whereas our model combines their two models already. Furthermore, in order to constrain the Total H_2S concentration profile, which had not been presented in Refs.[4,19] but was available from other sources (BCO-DMO ERDDAP database), we added three parameters to the model, including $k_{Fe2H2S} = 0.00007$, $k_{FeSH2S} = 0.14$ and $k_{FeOH3HRH2S} = 0.0002$. These parameters did not affect the concentration profiles

of other dissolved or particulate species based on a preliminary manual sensitivity analysis that we carried out. The proposed features in our conceptual model (main text Fig. 1) were then added in eight steps, as listed in Table S6, and the new model parameters (e.g., DOC and GPS degradation rates) were considered as free (tunable) parameters in the calibration process. For each step of adding complexity (adding new parameters), we calculated the Akaike information criterion (AIC)[74] and/or model selection criterion (MSC) to check whether or not the improvement in the goodness-of-fit has been efficient enough with regard to the added complexity.

AIC can be calculated as follows[74,75]:

$$\mathrm{AIC} = n_{obs}\,\mathrm{Ln}(\sigma^2) + 2n_{par} + \frac{2n_{par}(n_{par}+1)}{n_{obs}-n_{par}-1} \tag{S24}$$

where n_{obs} in the number of observation data, σ^2 is the sum of squared residuals divided by n_{obs}, n_{par} is the number of parameters estimated in the inverse modeling process and *Ln* is the natural logarithm. In comparing the performance of different models, the best model which has the best goodness-of-fit value using the fewest number of adjustable parameters yields the lowest AIC value.

The model selection criterion, which is based on AIC, can be calculated as follows[76,77]:

$$\mathrm{MSC} = \mathrm{Ln}\left(\frac{\sum_{j=1}^{n_{obs}} w_i (X_j^o - \overline{X^o})^2}{\sum_{j=1}^{n_{obs}} w_i (X_j^o - X_j^m)^2}\right) - \frac{2n_{par}}{n_{obs}} \tag{S25}$$

where w_i is a weight factor that is canceled in the equation by assuming equal weight for all observations. Considering Eq. (S23), MSC can then be expressed in terms of NSE as:

$$\mathrm{MSC} = \mathrm{Ln}\left(\frac{1}{1-NSE}\right) - \frac{2n_{par}}{n_{obs}} \tag{S26}$$

When comparing the performance of different models, the best model with the highest goodness-of-fit value and the fewest number of adjustable parameters yields the highest MSC value (opposite to AIC).

The incremental addition of complexity to the model is summarized in eight steps, with the results of NSE, MSC, and calibrated parameter values presented in Table S6. Concentration profiles resulting in steps 1, 2, and 7 are shown in Figs. S9, S10 and S11, respectively. According to these results, the mean NSE of all output profiles increases almost steadily from the first step of the model evolution (i.e., without adding any new parameters) towards step 7 (with all features included)—from 0.456 to 0.588. This improvement in the model fit to the addition of complexity

may be justified with the MSC[74,76] as similar to NSE, MSC calculated for all species displays a rising trend from Step 2 being 0.626 to Step 7 being 0.843. Although the model in Step 7 shows a better overall fit to all species profiles, further adjustment of parameters was made in Step 8 to improve the match between the modeled and the field profiles of total DOC. It should be noted that the model developed at Step 8 best describes the total DOC profile because of yielding the highest NSE for this profile, and this is also conceptually the most comprehensive among different steps. Thus, we chose the results of Step 8 as the basis for some of the parameter ranges in the Monte Carlo sampling process (Table S1).

We should note that whilst up to Step 7 we have considered the model fit to the concentration profiles of all species, (total DOC, total POC, SO_4, CH_4, DIC, H_2S, Fe^{2+}, Fig. S11), at Step 8 we have considered only total DOC concentration as the target data in the model fitting because total DOC concentration is also a proper representative of the majority of the processes that affect DOC cycling, including sorption/preservation, geopolymerization, hydrolysis, etc. DOC concentration has a mutual effect on MOC concentration and thus even though MOC concentration has not been included directly in the target data during the fitting procedure, still, the sorption/preservation process has been fully taken into account in the fitting procedure through the DOC concentration profile. Indeed, it is common practice in the literature of sorption to consider the concentration data of dissolved species instead of sorbed species when fitting different models to experimental data[78,79]. It is also common in the context of contaminant transport in porous media where the models are fitted to the concentration of transported species from experimental porous media (what is called the breakthrough curve) rather than the concentration of retained or sorbed mass[24].

By changing the target data from the concentrations of all species to only the total DOC concentration, there are only slight changes to a few of the optimized parameters from Step 7 to 8 and those are mainly related to sorption parameters (Table S6). After all, the impact of the tuned parameter values in the fitting procedure on the results of the present study is minor because ranges are sufficiently widened, e.g., for *ksorp* the lower range has been extended to a near zero value (1.0×10^{-15}) and even though it only has a non-zero value for GPS_1 and GPS_2 in the model fit process (18.5 and 2.00, respectively, Table S6), we extend the range of 1.0×10^{-15} to $18.5\times5=92.5$ to all *ksorp* parameters related to DOC_1, DOC_2, DOC_3, DOC_4, rDOC, GPS_1 and GPS_2.

It should be noted that our model does not explicitly consider the catalysis effect of iron and manganese on the geopolymerizaion process[80]. Nonetheless, model-generated GPS production rates shown in Supplementary Fig. S15 demonstrate a single peak at the depth of 5 cm, which is close to the iron availability zone in marginal sediments[80]. While the catalysis impact of iron and manganese on geopolymerizaion should be considered in future studies, the fact that the GPS production rate profile peaks near the surface suggests that iron and manganese availability depth does not significantly affect the validity of the present approach.

S2.3. Validation of the use of artificial neural network

The ANN process importance analysis based on the PaD method coded in MATLAB was previously validated against data generated from a basic mathematical equation and experimental-modeling literature data[1]. Here the ANN model could fit the data in all cases of process importance analysis with the best predictive fit NSE in the range of 0.923 to 0.944 and the optimum number of hidden layer neurons in the range of 6 to 20, as shown in Table S7. The scatter plots and error histograms for ANN fit the data corresponding to the highest and the lowest fit NSE are obtained for lrDOC-MOC preservation rate and *PE* of POC+MOC, respectively, as shown in Fig. S12 as representative examples among other cases, i.e., preservation rates of (semi)labile DOC, GPS, and lrDOC (Table S7). This demonstrates that excellent matches are obtained between RTM output data generated through the Monte Carlo technique and the ANN. Therefore, the meta-model obtained in this procedure has learned the behavior of the complex RTM properly, with 68 parameters being varied in around 1450 model runs of the RTM. The uncertainties incurred by the variation in parameter values are determined as a 95% confidence interval and are shown as error bars in the process importance analysis results. These uncertainties range from 4.6% to 29.9% (12.6% on average) of the mean values for the cases investigated and shown in main text Fig. 3 and S13.

S2.4. Validation of the modeling processes using mass budgets

In order to track the total organic carbon (OC) mass budget in the sediment column at a given depth, L, we consider the rates of mass transfer as either fluxes or reaction/conversion rates of different species. To show how these are calculated, we simplify our conceptual model presented in main text Fig. 1 to illustrate the mass flow in the model in Fig. S14. Here the control volume is considered from the sediment surface ($z = 0$) to the depth of L ($z = L$), herein considered at 1 m.

We then consider three cross sections to check the mass budget, including A-A, B-B, and C-C shown in Fig. S14.

The OC mass budget across the cross-section A-A (MB_{A-A}) is as follows:

$$MB_{A-A} = J_{POC0} \tag{S27}$$

J_{POC0} is the flux of POC (sum of all pools) into sediments at the surface (μmol.cm^{-2}.yr^{-1}). This is, in fact, the total flux of the OC entering the sediment and is thus used to check the mass budgets.

At the cross-section B-B, the OC mass budget is:

$$MB_{B-B} = J_{POCL} + \int_{z=0}^{z=L} R_{POC\to DOC} \tag{S28}$$

where J_{POCL} is the flux of the POC at a depth of L, $R_{POC\to DOC}$ is the rate of conversion of POC to DOC (μmol.cm^{-3}.yr^{-1}) which is then integrated over the depth of the sediments, L. This integration makes the unit of the second term in Eq. (S28) consistent with the unit of flux (μmol.cm^{-2}.yr^{-1}). This calculation involves all pools of POC and DOC (including (semi)labile DOC, GPS and lrDOC).

At the cross-section C-C, the OC mass budget is:

$$MB_{C-C} = J_{POCL} + J_{DOC0} - J_{DOCL} + \int_{z=0}^{z=L} R_{DOC\to DIC} + \int_{z=0}^{z=L} R_{DOCSorp} \tag{S29}$$

where J_{DOC0} is the efflux of DOC from the sediment surface, J_{DOCL} is DOC flux at a depth of L, $R_{DOC\to DIC}$ is the rate of DOC remineralization to DIC and $R_{DOCSorp}$ is the net DOC kinetic sorption rate (or MOC formation rate which is sorption rate minus desorption rate). It should be noted that J_{DOCL}has a negative sign in the equation above because the mass is added to the control volume via this term, whereas the mass is removed from the system via all other terms. This calculation also involves all pools of POC and DOC (including (semi)labile DOC, GPS and lrDOC).

Based on Monte Carlo model runs (1450 realizations, Stage 1), the results averaged for all runs give mean values of $MB_{A-A} = 57.136$ μmol.cm^{-2}.yr^{-1}, $MB_{B-B} = 57.197$ μmol.cm^{-2}.yr^{-1}, and $MB_{C-C} = 57.189$ μmol.cm^{-2}.yr^{-1}, which demonstrates an overall mass balance error of ~0.1% which is less than the acceptable mass balance error of 1% considered in our general modeling process.

S2.5. Model assumptions and sensitivity of results to these

We have followed standard practice on different aspects of the model development and modeling procedure, which inherently contain assumptions. We adopted these practices and assumptions based on previous literature or logic that we believe better represents nature than previous studies, e.g.:

i. **The concept:** We have based our conceptual model (Fig. 1) upon logics that are deemed to be better applicable to carbon cycling in marine sediments than the previous concepts. For instance, previously, DOC breakdown (hydrolysis) has been considered as a series of parallel reactions[4], whereas here we considered this as a series of consecutive chain-reaction expressions which work serially and not parallelly. This is in line with the literature on water column carbon cycling[5]. The overall model fits to the field data using the consecutive chain-reaction model show a similar to better match compared to using the parallel pool concept considered in previous modeling studies (see Table S6).

ii. **The number of pools:** In our conceptual model (Fig. 1), we have considered an unknown number of pools for all species to better represent nature. Whilst maintaining the overall structure of the conceptual model and the accurate representation of natural conditions, to follow the principle of parsimony, in our modelling approach, we have selected the minimum number of pools for different species based on a trade-off between the accuracy and feasibility/efficiency of model runs. Particularly, to make sure our model represents nature, we selected the reactivities of DOC pools based on their lifetimes in the water column as categorized in field measurements similar to labile and semi-labile (DOC_1, DOC_2…, grouped as (semi)labile-DOC for simplicity of discussion (lifetime of ~9 hours to ~1.5 years)), mid-reactive (GPS_1, GPS_2… with a lifetime of ~20 years) and least-reactive (lrDOC with a lifetime of ~16000 years)[23]. We have further provided evidence that our model outputs are not sensitive to an increased number of pools for DOC compared to the minimum number that we had considered already (Figs. S17, S18).

iii. **The parameterization:** The principle of parsimony (also known as Occam's Razor) suggests that when there are multiple alternative models, the one with the lowest number of parameters is preferred. By reducing the number of pools, we have also mostly reduced the number of unknown parameters. Importantly, we developed our model on a stage-by-stage basis and used the Akaike information criterion (AIC)[74] and/or model selection

criterion (MSC) to ensure that we follow the principle of parsimony in our model development as discussed in Section S2.2 and summarized in Table S6. We conducted a comprehensive sensitivity analysis of the model parameters that resulted in the process-importance analysis (Fig. 3, Section S1.3).

We acknowledge that there are still some assumptions in our modeling which cannot be explicitly verified, including the consecutive chain-reaction model equations (Eqs. S17-S20) used for hydrolysis and geopolymerization that have been taken from a broader context and may need further validation for the specific case of marine sediments, yet we provide a comparison of our results to recent literature with respect to GPS in Section S.5.

S3. Calculation of ratios used in the profiles of Figure S16 and in the process importance analysis

The ratios or percentages used for sketching the profiles within the panels of main text Fig. 4 are calculated as follows:

Panel A. The ratio of the kinetic sorption mass-transfer rate to desorption mass-transfer rates presented in main text Fig. 4a is described as follows:

$$Ratio_{\frac{Sorp}{Desrop}} = \left(\int_{z=0}^{z=L} R_{DOCSorp}\right) \cdot \left(\int_{z=0}^{z=L} R_{MOCDes}\right)^{-1} \tag{S30}$$

where R_{MOCDes} is the MOC kinetic desorption rate. Here DOC is either (semi)labile DOC, GPS, or lrDOC, and MOC is either (semi)labile DOC-MOC, GPS-MOC or lrDOC-MOC.

Panel B. The percentage of GPS contribution of the total lrDOC production rate presented in main text Fig. 4b is as follows:

$$P_{\frac{GPS\ Contribut}{rDOC\ Prod}} = \left(\int_{z=0}^{z=L} R_{GPSp \to rDOC}\right) \cdot \left(\int_{z=0}^{z=L} R_{GPSp \to rDOC} + \int_{z=0}^{z=L} R_{DOC1 \to rDOC}\right)^{-1} \times 100 \tag{S31}$$

where $R_{GPSp \to rDOC}$ is the rate of conversion of the terminal GPS species (GPS_p, see main text Fig. 1) to lrDOC and $R_{DOC1 \to lrDOC}$ the rate of conversion of DOC_1 to lrDOC representing the intrinsically refractory fraction of DOC_1 (leftovers). The unit of all the rates mentioned above is in $\mu mol.cm^{-3}.yr^{-1}$ which after integration becomes $\mu mol.cm^{-2}.yr^{-1}$.

It should be noted that to calculate the ratio in Panel A, we used the ratio of means of sorption rates and means of desorption rates for the outcomes of the Monte Carlo stage. In panel B, we used the mean of the percentages obtained in different Monte Carlo runs. In Panel A, we used the ratio of means because sorption and desorption occur in parallel, and the numerator could be much larger than the denominator for some of the individual cases of the Monte Carlo output data yielding extremely high ratios. Such large values could control the average of ratios, thereby yielding unreasonable results, whereas the mean value of sorption rates and the mean value of desorption rates were not affected by the extreme values in the Monte Carlo outcomes, nor were the ratios of these means.

S4. Detailed discussion on where and how sorption and geopolymerization control OC preservation

Several different factors have been proposed to explain OC preservation in marine sediments[11,17,81-85], e.g., oxygen availability[81], physical restriction[82], mineral sorption[11], polymerization[80,84,86], selective preservation[11], iron minerals[83], other minerals[87] and biological factors[88]. In the present study we reveal the importance of kinetic sorption and geopolymerization for OC preservation as outlined in Section titled "The role of different processes in carbon preservation" in the main text. Here we further use sediment depth profiles (Fig. 4 and Supplementary Section S3) from the Monte Carlo simulations to provide a broader insight into how these processes control OC preservation.

S4.1. Mineral shuttle

The ratio of kinetic sorption rate to desorption rate (Fig. 4A) may indicate where the sorption is more effective with depth in the sediment. The effectiveness of sorption for all DOC constituents exhibits maxima within the top 10 cm of sediments, corresponding to the surface mixed layer, and then decreases steadily with depth by a factor of 3 – 5 down to 1-m depth. These results clearly demonstrate that minerals in the mixed layer act as a shuttle for different DOC pools by protecting them from exposure to oxygen, nutrients and microbial enzymes and, consequently, limiting their rapid remineralization in the mixed layer and delivering them to greater depths (Fig. 4A). The peak in sorption effectiveness is explained by contrasting rates of sorption and desorption. The former increases rapidly within the mixed layer and less rapidly below this depth (Fig. S16A), whereas

desorption rates increase monotonically with depth Fig. S16B). The peak for (semi)labile DOC appears largest and earliest compared to lrDOC, followed by that of GPS, appearing at approximate depths of 2.5 cm, 5 cm, and 7.5 cm, respectively (Fig. 4A). This is because the gradient of sorption rate, becomes milder for (semi)labile DOC compared to lrDOC and GPS toward deeper depths relative to the surface mixed layer (Supplementary Fig. S16). The role of sorptive preservation of labile OC has been highlighted before[12], although its reversibility remains enigmatic[33,84,85,89]. Here our analysis demonstrates that the ratio of sorption to desorption kinetic rates, defined as sorption effectiveness, is larger than 1 throughout the investigated depth of sediments (Fig. 4A). This suggests that the net result of these processes leads to the formation and accumulation of MOC over time, and implies that the seemingly concurrent equilibrium and irreversible sorption observed in previous studies[12,84,85,90] can in fact be the net result of kinetic sorption-desorption processes being dominated by sorption. Our results also show that the mineral shuttle contribution to OC preservation is greater for (semi)labile DOC compared to lrDOC followed by GPS.

S4.2. Pathways of least-reactive DOC production

The contribution of geopolymerization to lrDOC formation shown in Fig. 4B displays a substantial increase with sediment depth and reaches 16.3% by 100 cm. The remaining 83.7% of lrDOC production corresponds to the intrinsically undegradable DOC fraction (see Fig. 1). Organic carbon preservation as lrDOC is thus governed by intrinsically undegradable DOC. lrDOC produced at depth may be more important in preservation compared to that produced near the surface. lrDOC produced close to the sediment surface can be more easily transported to the water column via diffusion compared to lrDOC produced at depth, increasingly by geopolymerization. The increase in the contribution of geopolymerization to lrDOC formation with depth may thus point to the importance of geopolymerization in preserving OC and help to explain the origin and formation of kerogen and fossil carbon in marine sediments[17].

S4.3. Burial is the start of preservation, not the end

The contribution of MOC to the solid OC (POC+MOC) increases with depth, e.g., at 10 cm, 25 cm, 50 cm and 100 cm, it is around 22%, 37%, 49%, and 60% (Fig. 4D). This results in *PE* estimated at 1-m depth for POC+MOC to be 2.7 times *PE* for POC alone (Fig. 4C). The pronounced increase of MOC within the top 10 cm of sediments is consistent with the ratio of

sorption to desorption rates being maximum in this layer (Fig. 4A), corroborating the concept of a mineral shuttle playing an important role in carbon preservation within marine sediments.

S5. Comparison with previous studies and directions for future research

Here we compare the estimation of our study about GPS contribution to OC burial with that of Moore et al.[80]. POC deposition flux at the sediment-water interface has been reported to range from 200 to 5000 Tg C yr^{-1},[17,91-93] whilst mean conventional burial efficiency has been known to be ~20%[94] giving burial flux of POC in the range of 40 to 1000 Tg C yr^{-1}. Considering GPS formation rate from Moore et al.[80] in the oxygenated zone of sediments being 4.05 Tg C yr^{-1}, GPS contribution to buried carbon may thus range from 0.4% to 10%. Our model runs provide a ratio of net GPS sorption to total solid OC (MOC+POC) buried at depths ranging from 0.05 to 1 m depths within the range of 2.7±0.2% and 18.0±0.8%. Our model estimation is thus close to that based on Moore et al. On the one hand, our estimates might be more representative of the GPS contribution to OC burial because we take into account the whole sediment to the depth of 1 m, compared to Moore et al. only considering the oxic zone. On the other hand, the chain-reaction model equations that we use for geopolymerization have not been explicitly verified for marine sediments. The take home message, however, is that both Moore et al. and our model results show that GPS production is a small but important part of OC preservation.

The ANN modeling stage, in addition to providing insights into the importance of different processes, generates empirical networks that can be used for global prediction of each of the output parameters discussed (e.g., DOC fluxes or net sorption rates) with minor computation expense in future studies or be integrated within other numerical models, such as deep-time paleo models (e.g., SCION[94]), ocean biogeochemical models (e.g., PISCES[95]) and global circulation models (GCM)[96]. Such models may allow for investigating complex factors such as sediment morphology dynamics on carbon cycling that require a combination of three-dimensional sediment-water column models. Future studies may also consider more surface interaction theories and concepts to quantify the strength of associations between OC and minerals as well as the role of colloids and microbes[97,98].

Figures and Tables

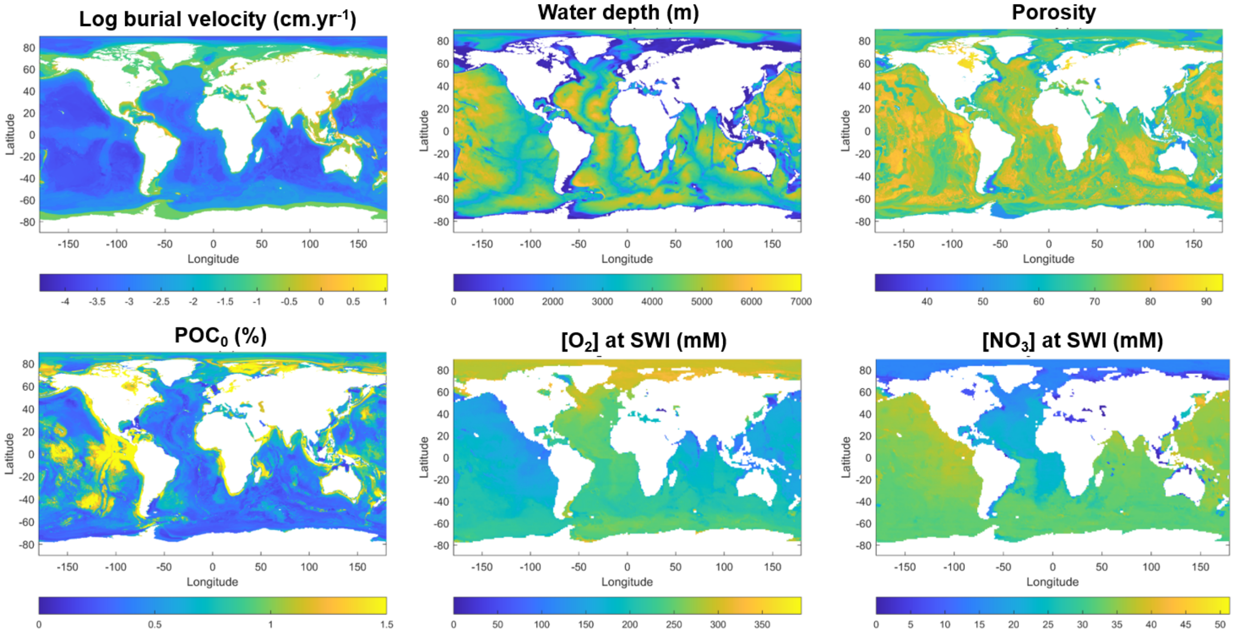

Fig. S1. Maps for six global grid datasets (with a resolution of 1°×1°) including sediment accumulation rate or burial velocity[57], water depth[56], porosity in the upper 5-cm of seafloor[58], sediment-water interface concentrations of POC[58], NO_3 concentration at the sediment-water interface (SWI) Ref.[59,60], and O_2 concentration at SWI Ref.[59,60].

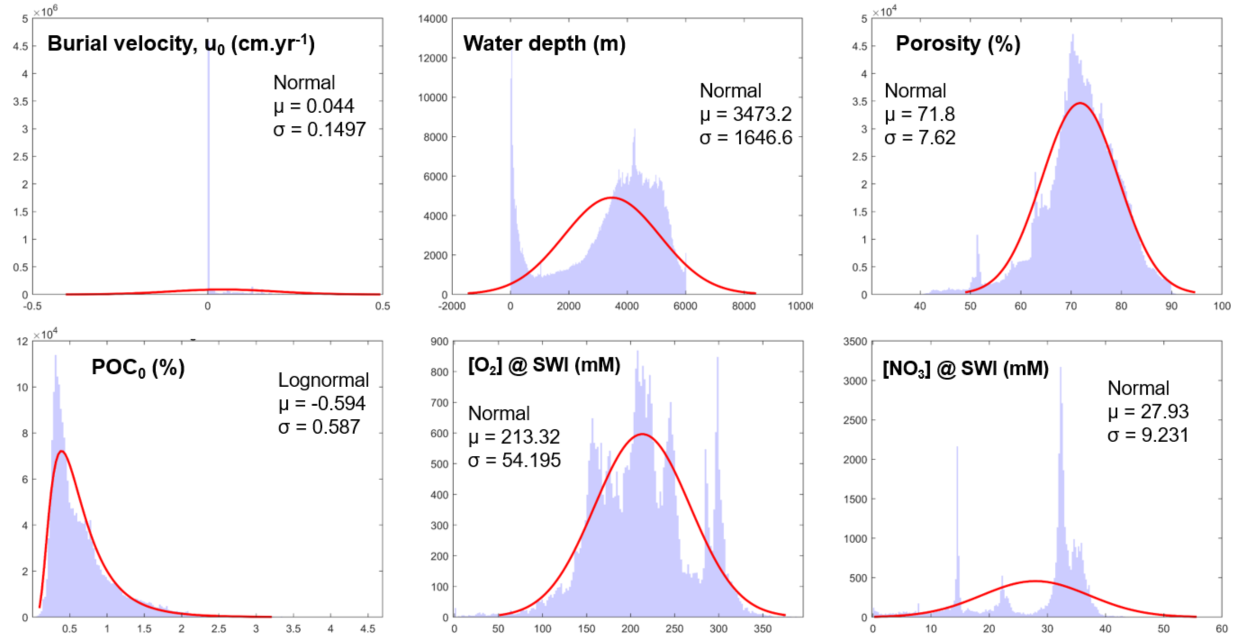

Fig. S2. Selected probability distributions fit to the six global grid datasets listed in the caption of Fig. S1 based on a trade-off between goodness-of-fit (distribution plots) and simplicity. The types of the distributions, their mean (μ) and standard deviation (σ) for each case are shown in each panel.

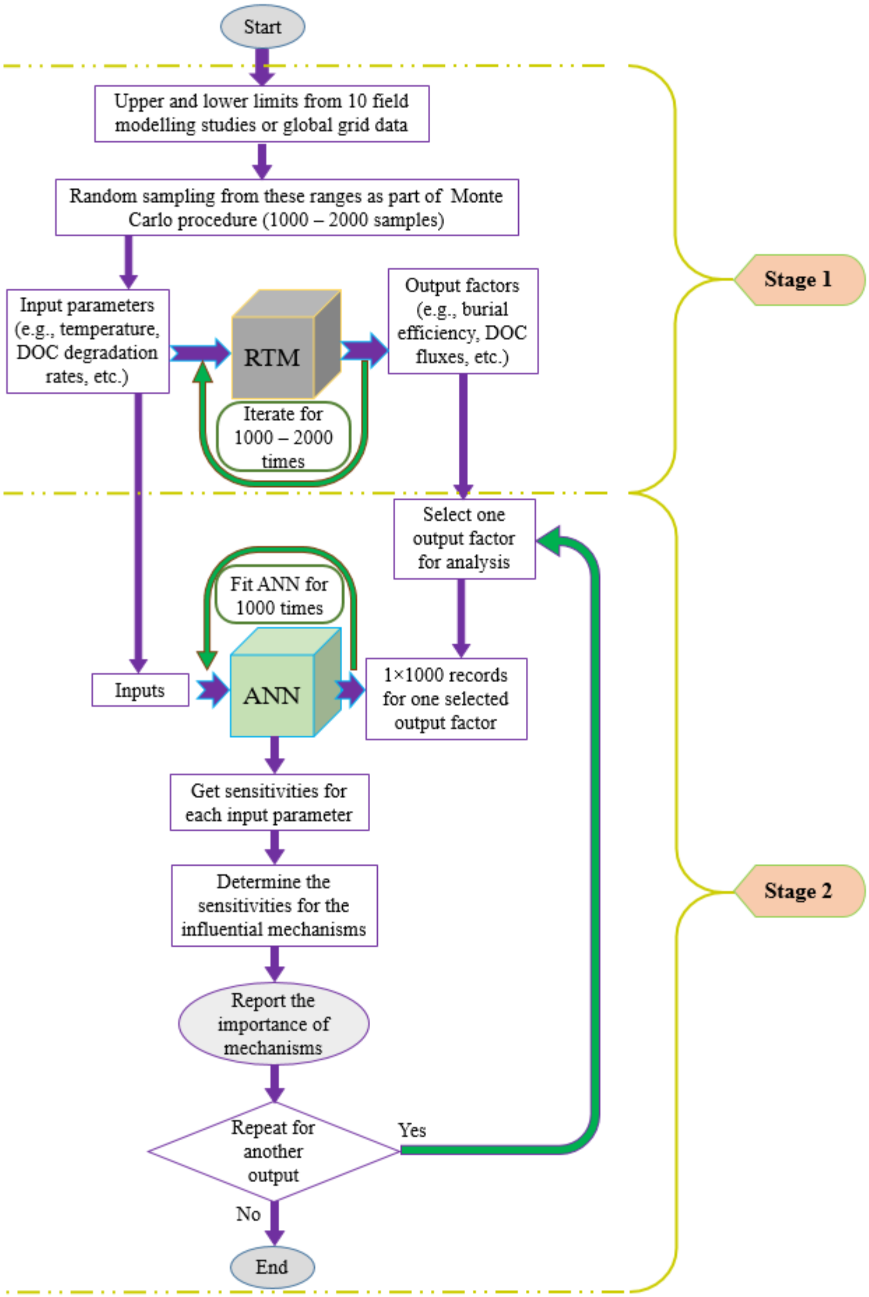

Fig. S3. The overall flowchart of the modeling algorithm showing the two stages of the modeling procedure. The validated reaction transport model (RTM) is executed multiple times to generate a model database of preservation efficiencies (*PE*) or preservation rates of MOC at 1 m depth (Stage 1), which is used to train an Artificial Neural Network (ANN) to determine the influential factors of *PE* or preservation rates considering six different groupings of parameters (Stage 2). See main text for full description.

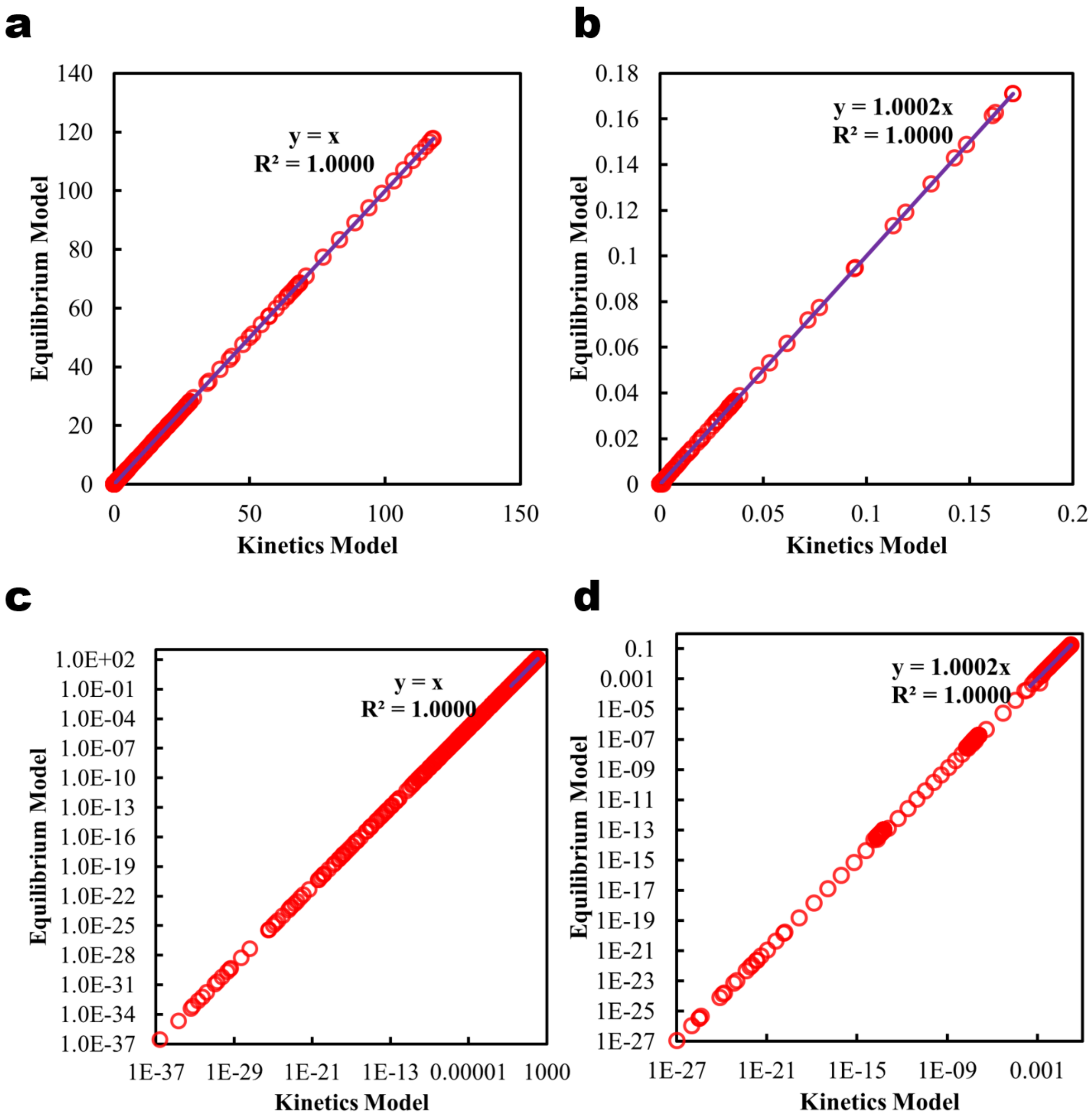

Fig. S4. Validation of the kinetic sorption formulation developed in the present study against the equilibrium, linear isotherm model, which has already been used for adsorption in early diagenesis models[26,28] at a high mass transfer rate where it is expected that the kinetic model asymptotes to the equilibrium model. (a) The model output concentrations for all species except adsorbed species. (b) The model outputs for all adsorbed species. (c) and (d) Log-log transformed plots of (a) and (b), respectively. In these simulations, the model parameters and characteristics were selected from Ref.[18], and adsorption coefficients were assumed to be similar to Ref.[55].

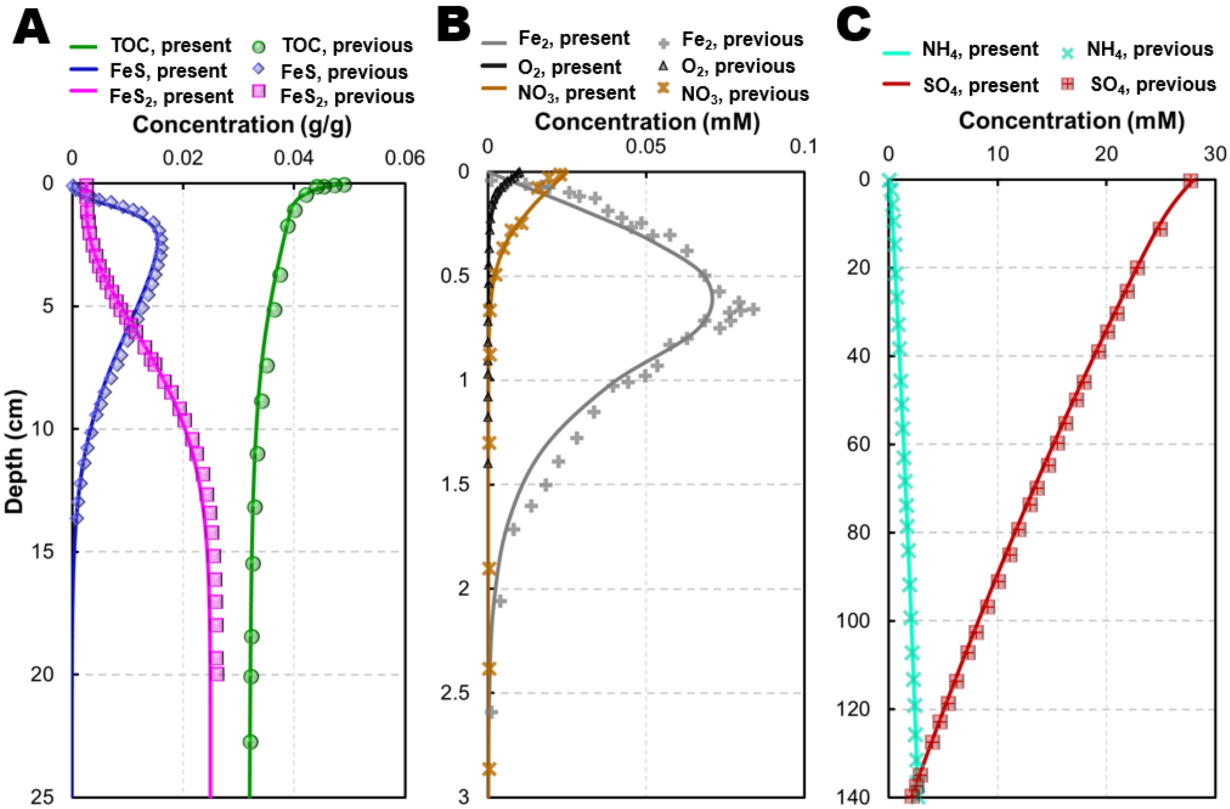

Fig. S5. Comparing the basic numerical model results with those of Meysman et al. [54]. The outputs of the present model are shown with continuous lines and those of the previous model (MEDIA (Modeling Early DIAgenesis) software package conducted by Meysman et al.[54]) are shown with markers for total organic carbon (TOC) and particulate FeS and FeS_2 (g/g, panel A) and some of the other dissolved constituents (mM, panels B, C).

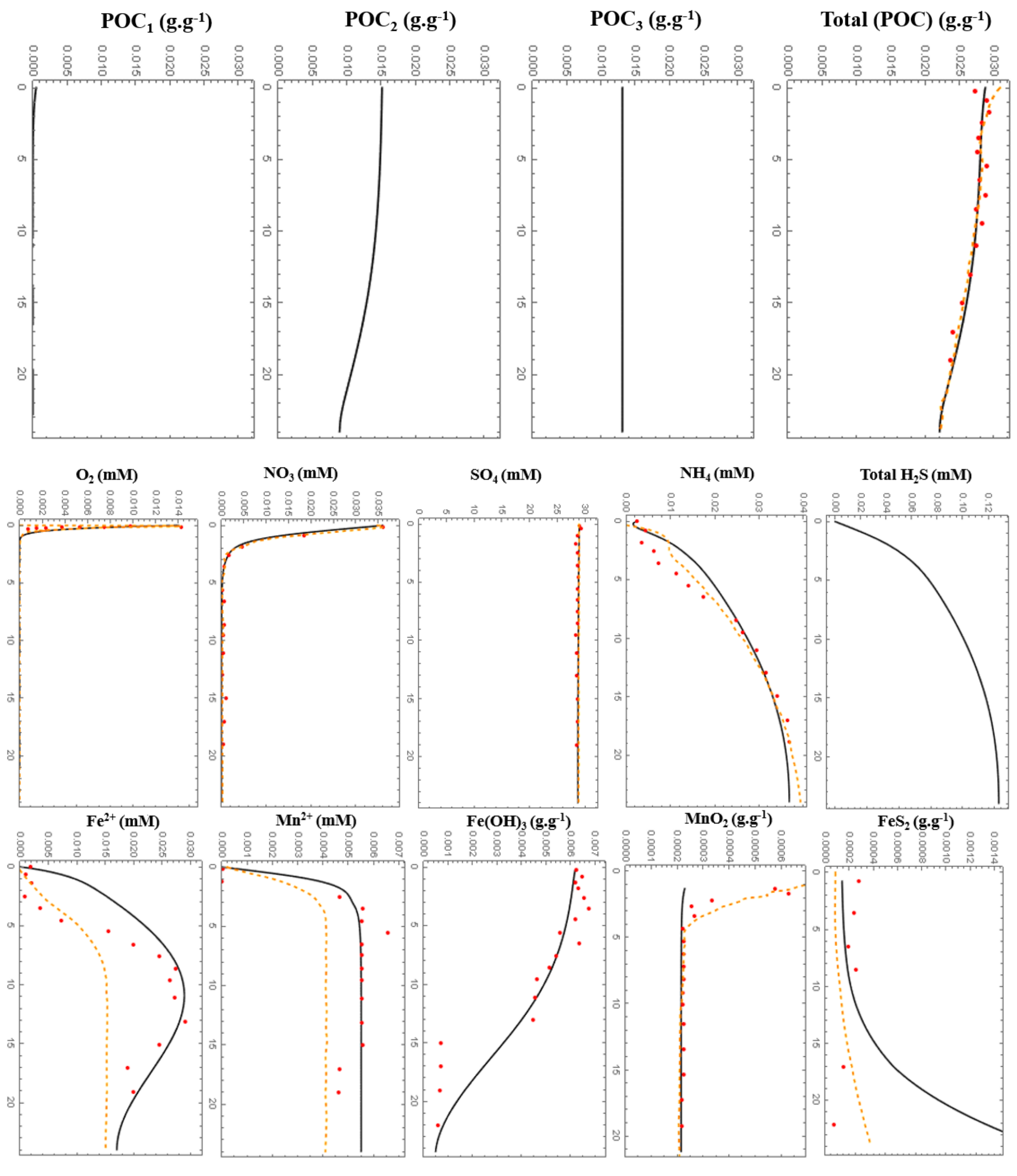

Fig. S6. Concentration profiles of the basic model (without DOC species) fitted to field data and compared with modeling data of Kraal et al.[50]. Here, we used R^2 as the objective function. The continuous black line represents our model curve, the red circles represent the field data of Kraal et al., and the dashed orange line represents the modeling outputs of Kraal et al. For the cases of POC_1 to POC_3, and Total H_2S, no field or previous measurement or modeling data is available.

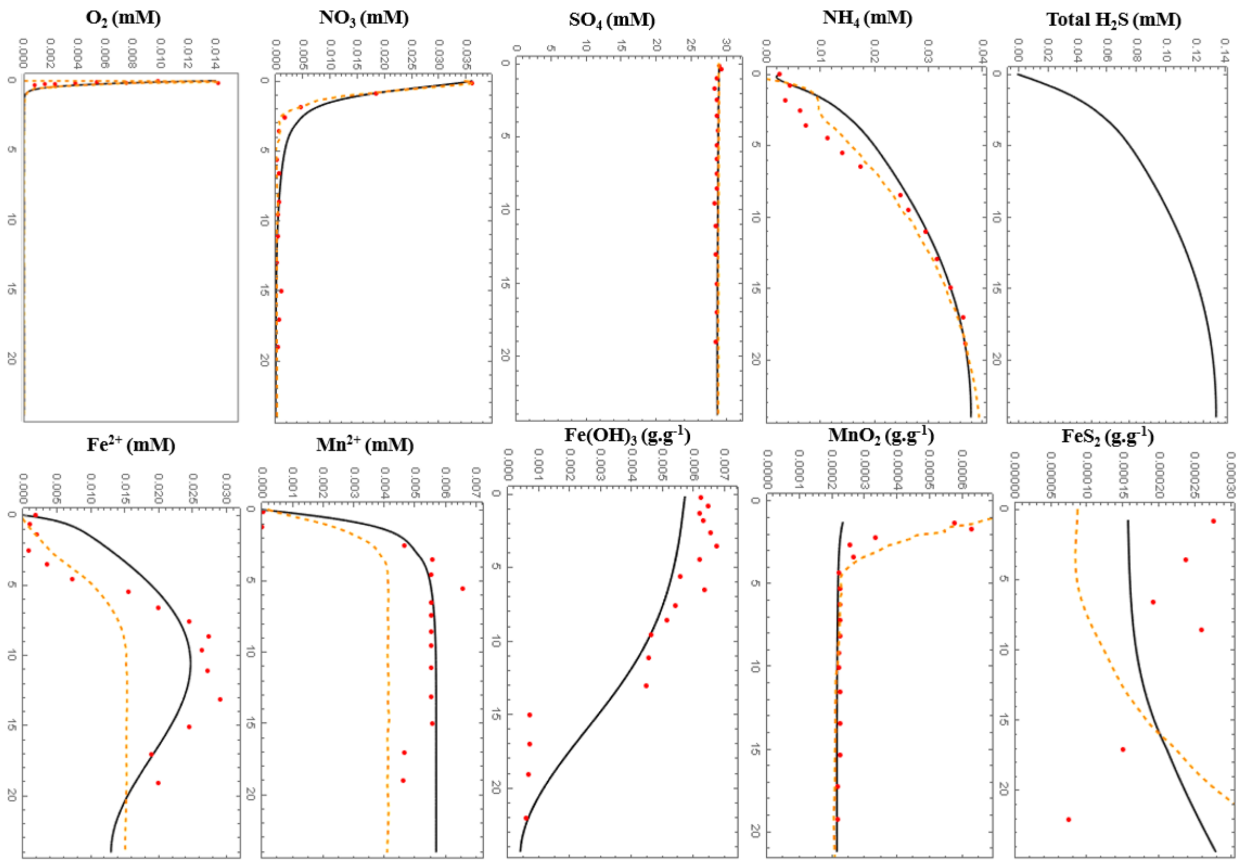

Fig. S7. Concentration profiles of the basic model (without DOC species) fitted to field data and compared with modeling data of Kraal et al.[50]. Here, we used NSE as the objective function. The black continuous line represents our model curve, the red circles represent the field data of Kraal et al. and the dashed orange line represents the modeling outputs of Kraal et al. For the cases of POC_1 to POC_3, and Total H_2S, no field or previous measurement or modeling data is available.

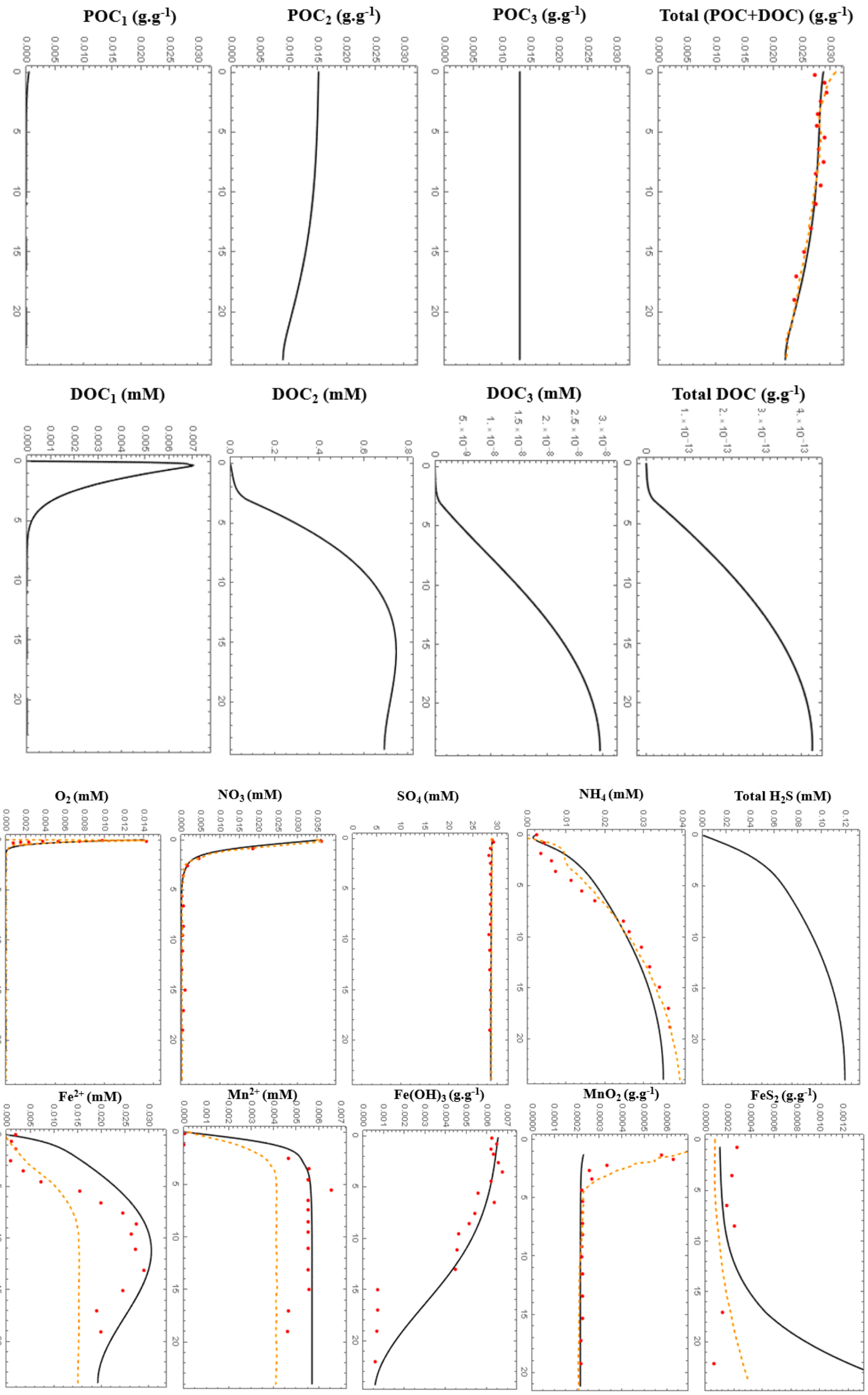

Fig. S8. Concentration profiles of the model with the basic consideration of DOC species fitted to field data and compared with modeling data of Kraal et al.[50]. The inclusion of DOC at this stage was a simplifying approach rather than considering full cascaded hydrolysis processes of DOC presented in our conceptual model (main text Fig. 1). The continuous black line represents our model curve, the red circles represent the field data of Kraal et al., and the dashed orange line represents the modeling outputs of Kraal et al. For the cases of POC_1 to POC_3, Total H_2S, and DOC species no field or previous measurement or modeling data is available.

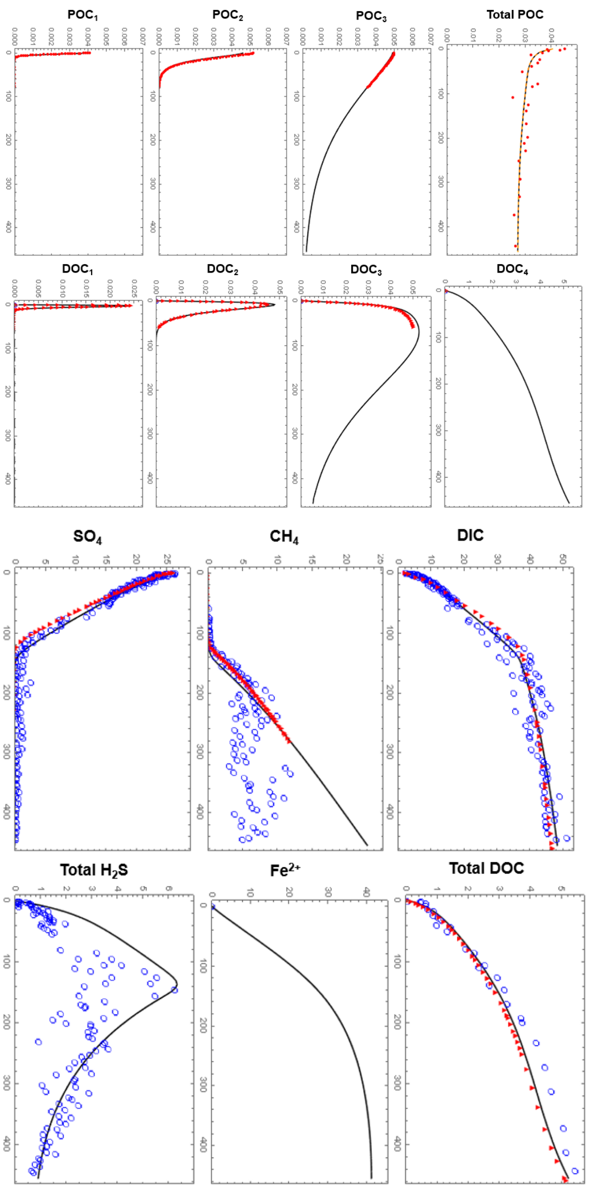

Fig. S9. Model results without involving adsorption, kinetic sorption and geo-polymerization mechanisms (Step 1, Table S6) fitted against field data and compared with modeling data of Refs.[4,19]. In this step, we used all features of the Burdige et al. model, including their conceptual model of DOC. Yet, they used two models separately: OMSN (Organic Matter/Sulfate/Nitrogen) model and DOC model. Here we combined their two models into one and further extended it to consider various processes. Furthermore, in order to constrain Total H_2S profile, which had not been presented in Refs.[4,19] but was available from other sources (BCO-DMO ERDDAP database), we added three parameters to the model. These parameters include kFe2H2S = 0.00007, kFeSH2S = 0.14, and kFeOH3HRH2S = 0.0002. These parameters did not affect the concentration profiles of other dissolved or particulate species in the model based on a manual sensitivity analysis that we carried out. In the figure, the continuous black line represents our model curve, the hollow blue circles represent the field data of Refs.[4,19] and the dashed orange line or solid red markers represent

their modeling outputs. Here, in the Total POC profile, MOC has not been added because in the early steps, sorption has not been included yet. For the cases of POC_1 to POC_3, DOC_1 to DOC_3 and Fe^{2+}, no field or previous measurement or modeling data is available.

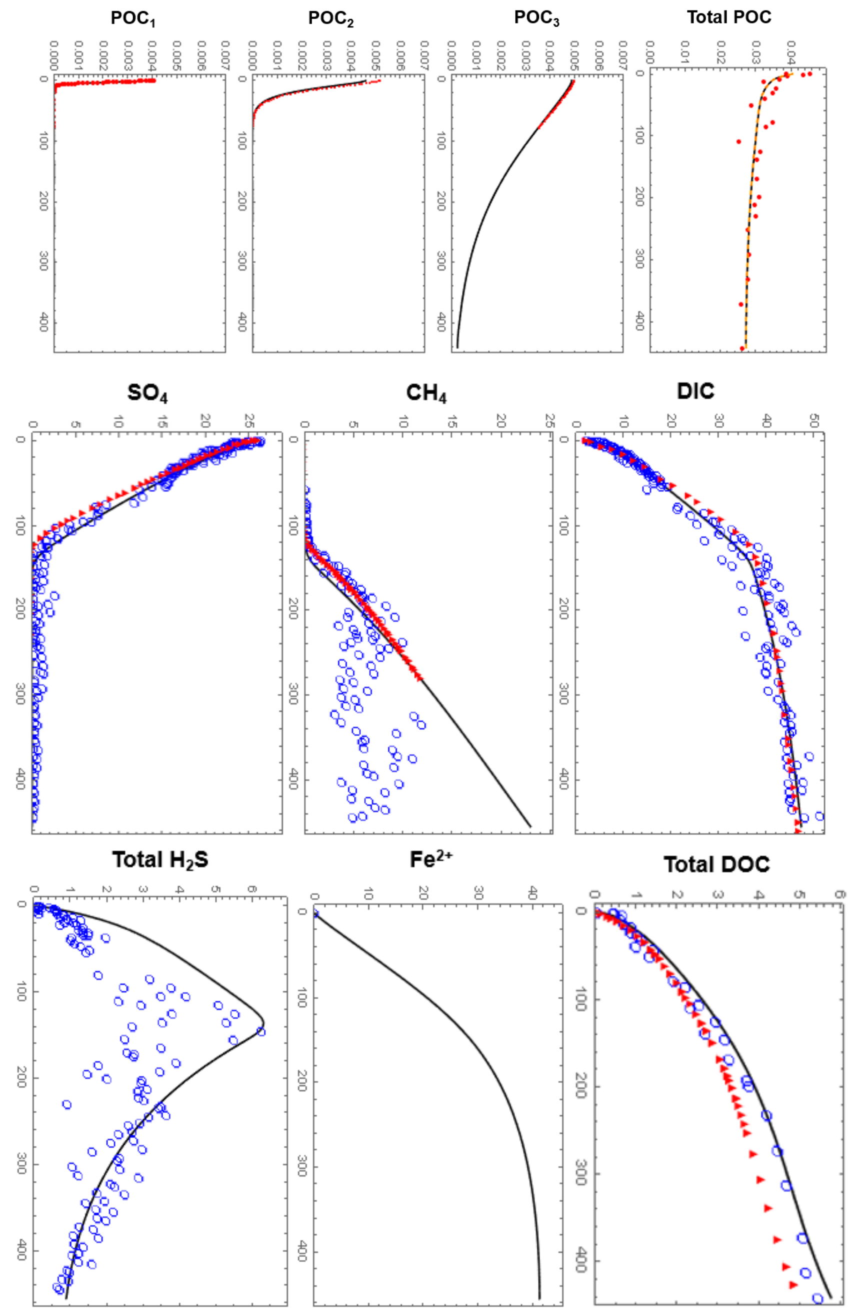

Fig. S10. Model results after considering DOC cascaded hydrolysis (Step 2, Table S6) fitted against field data and compared with modeling data of Refs.[4,19]. The continuous black line represents our model curve, the hollow blue circles represent the field data of Refs.[4,19] and the dashed orange line or solid red markers represent their modeling outputs. Here, in the Total POC profile, MOC has not been added because, in the early steps, sorption has not been included yet. For the cases of POC_1 to POC_3 and DOC_1 to DOC_3, no measurements and for Fe^{2+}, no measurement or modeling are available.

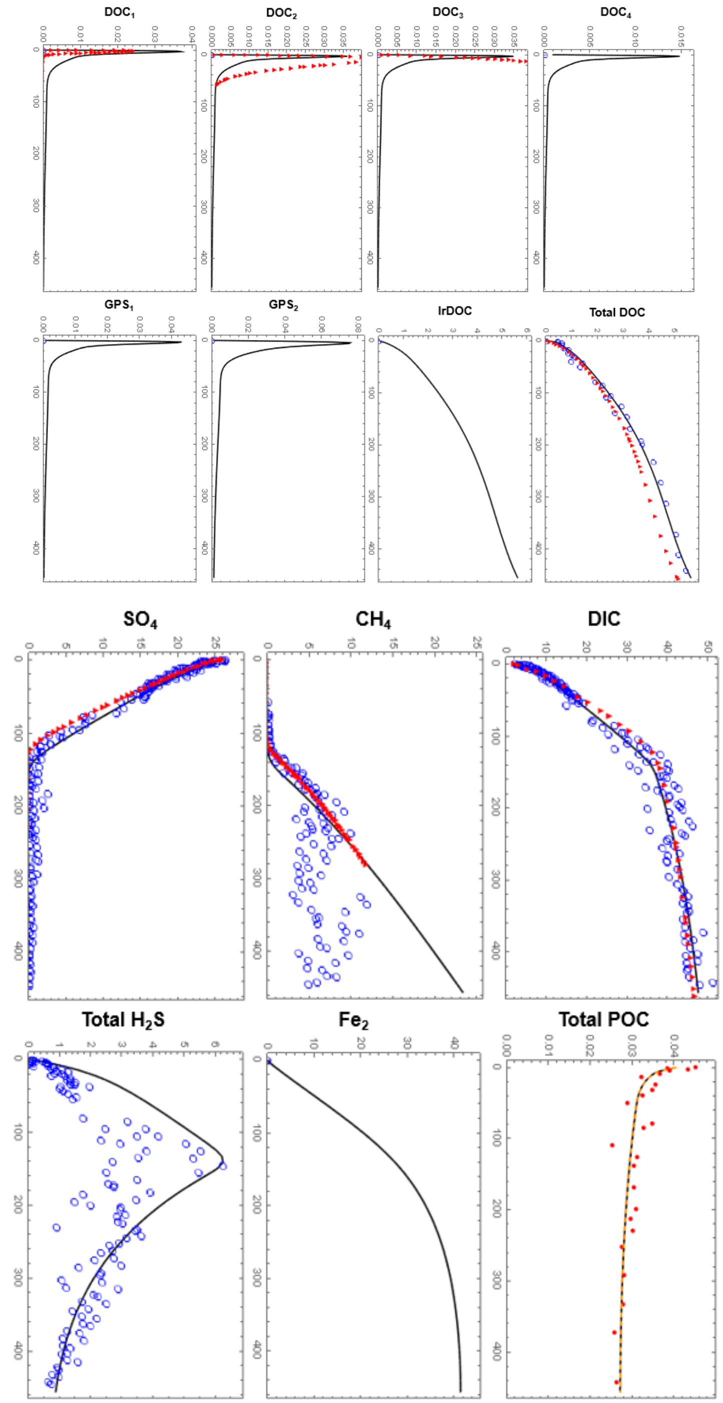

Fig. S11. Model results after considering DOC, GPS, lrDOC, and all mechanisms of adsorption, kinetic sorption, and geo-polymerization (Step 7, Table S6) fitted against field data and compared with modeling data of Refs.[4,19]. The continuous black line represents our model curve, the hollow blue circles represent the field data of Refs.[4,19] and the dashed orange lines or red solid markers represent their modeling outputs. Total DOC includes(semi)labile DOC, GPS, and lrDOC constituents. For the cases of GPS_1, GPS_2, lrDOC and Fe^{2+} no measurement or modeling are available.

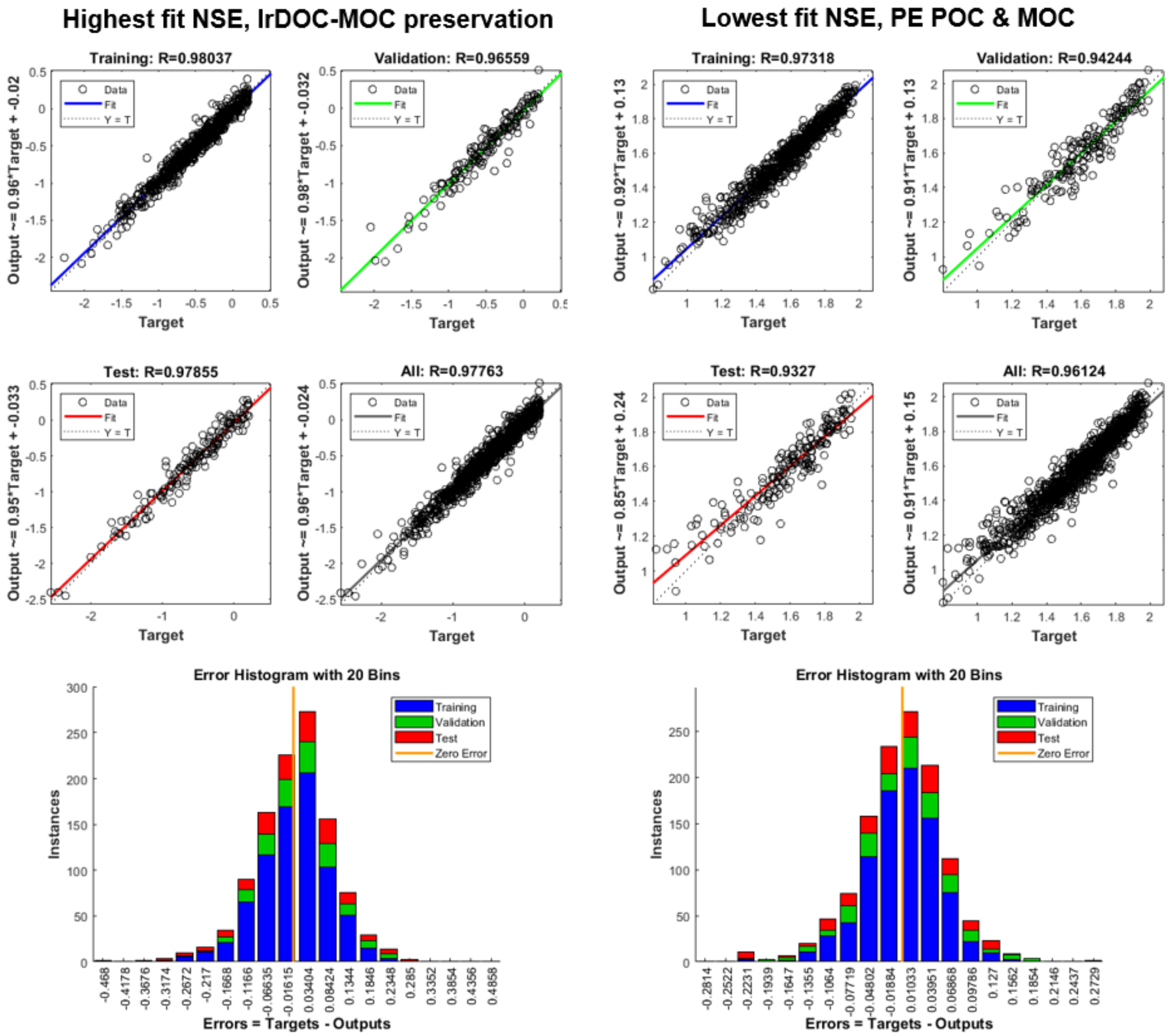

Fig. S12. The scatter plots (top) and error histograms (bottom) for ANN fits to different subsets of the data, including the training set (70% of the dataset selected randomly during the ANN modeling process), the validation set (15% of the dataset selected randomly during the ANN modeling process), and the test set (15% of the dataset selected randomly during the ANN modeling process)[1] for the cases with the highest and the lowest predictive fit NSE, corresponding to the cases of lrDOC-MOC preservation rate (left) and *PE* of POC plus MOC (right), respectively. These are shown as representative examples among all cases in Table S7.

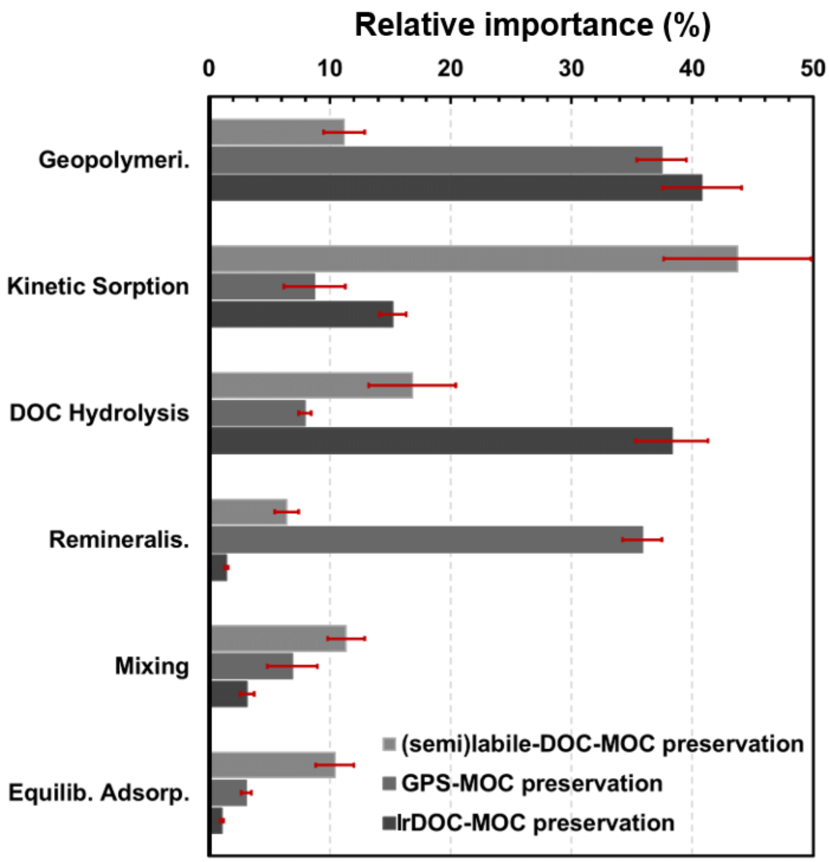

Figure S13. The relative importance of 6 different processes to preservation rates at 1 m depth for different fractions of MOC, including (semi)labile DOC-MOC, GPS-MOC and lrDOC-MOC. The six processes are DOC hydrolysis, DOC remineralization, mixing, equilibrium adsorption, kinetic sorption and geopolymerization. Error bars represent a 95% confidence interval based on 1000 runs of the process importance analysis at Stage 2 of the modeling procedure. The bars are sorted from the highest importance averaged for the three fractions of MOC (top) to the lowest importance (bottom). The averaged values of each three bars for the three fractions are given in main text Fig. 3B.

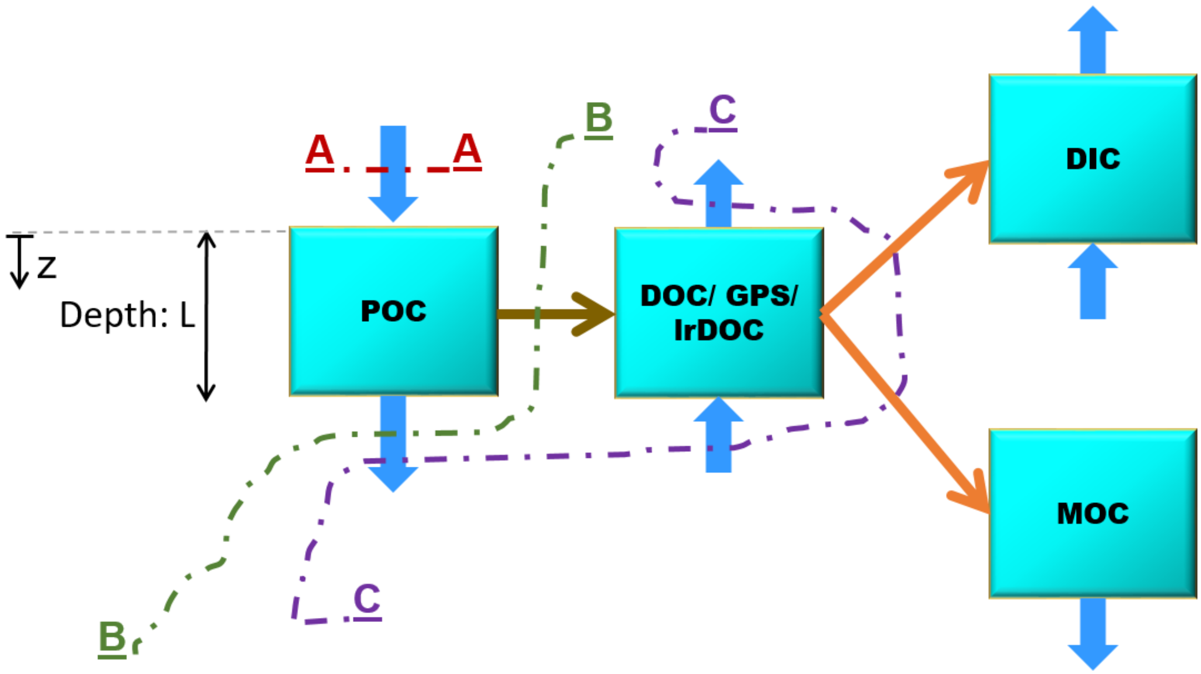

Figure S14. Simplified conceptual model to show the OC mass flow in the RTM. The rectangular boxes represent a control volume of the sediment with a depth of *L* for each species. Thick arrows represent fluxes in and out of the control volume and thin arrows represent reaction rates transferring mass to other species. Cross sections A-A, B-B and C-C are used to calculate the mass budgets for mass balance validation of the model in Section S2.4.

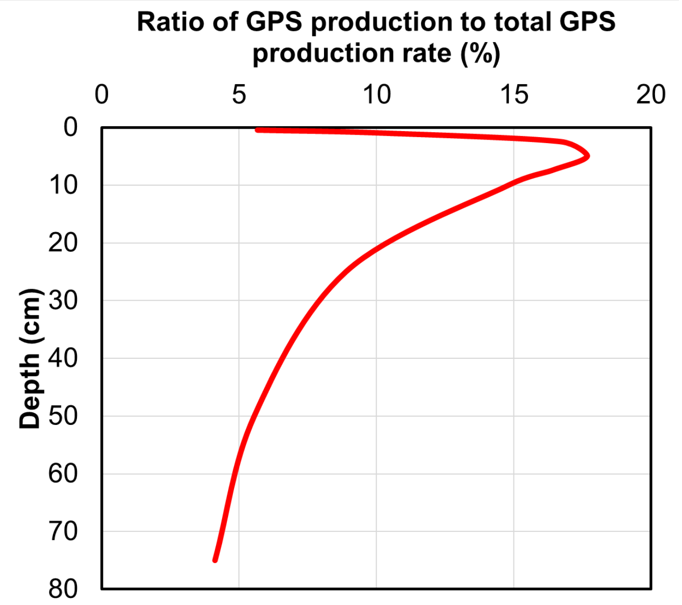

Figure S15. GPS net production rate normalized by the sum of GPS production rates over the depth. The rates are average results of 1450 model runs during the Monte Carlo stage of modeling

(Stage 1 of the modeling algorithm, Fig. S3). The results are averaged for GPS_1 and GPS_2. Net GPS production rate means GPS production rate minus consumption rate. These rates are divided by the sum of all production rates over the depth and are reported in percentage.

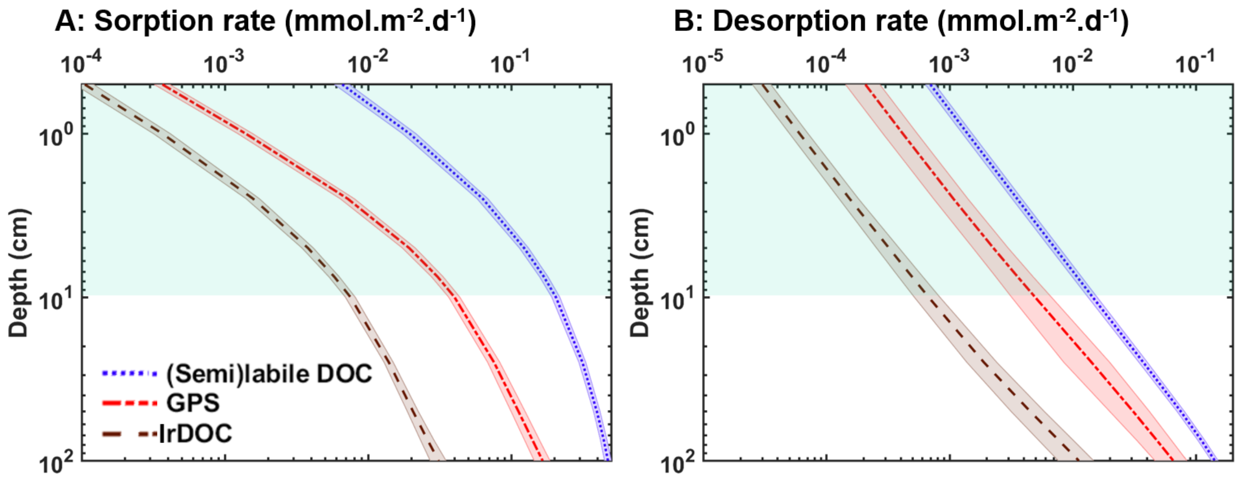

Figure S16. Kinetic sorption (**A**) and desorption (**B**) rates of (semi)labile DOC, GPS, and lrDOC. These are averaged results of 1450 model runs during the Monte Carlo stage of modeling (Stage 1 of the modeling algorithm, Fig. S3). The results for DOC are averaged across DOC_1 to DOC_4 and for GPS are averaged across GPS_1 and GPS_2. The light, blue-shaded region represents the mixed layer depth (10 cm). Shaded areas of the curves represent 95% confidence intervals obtained from Monte Carlo model runs.

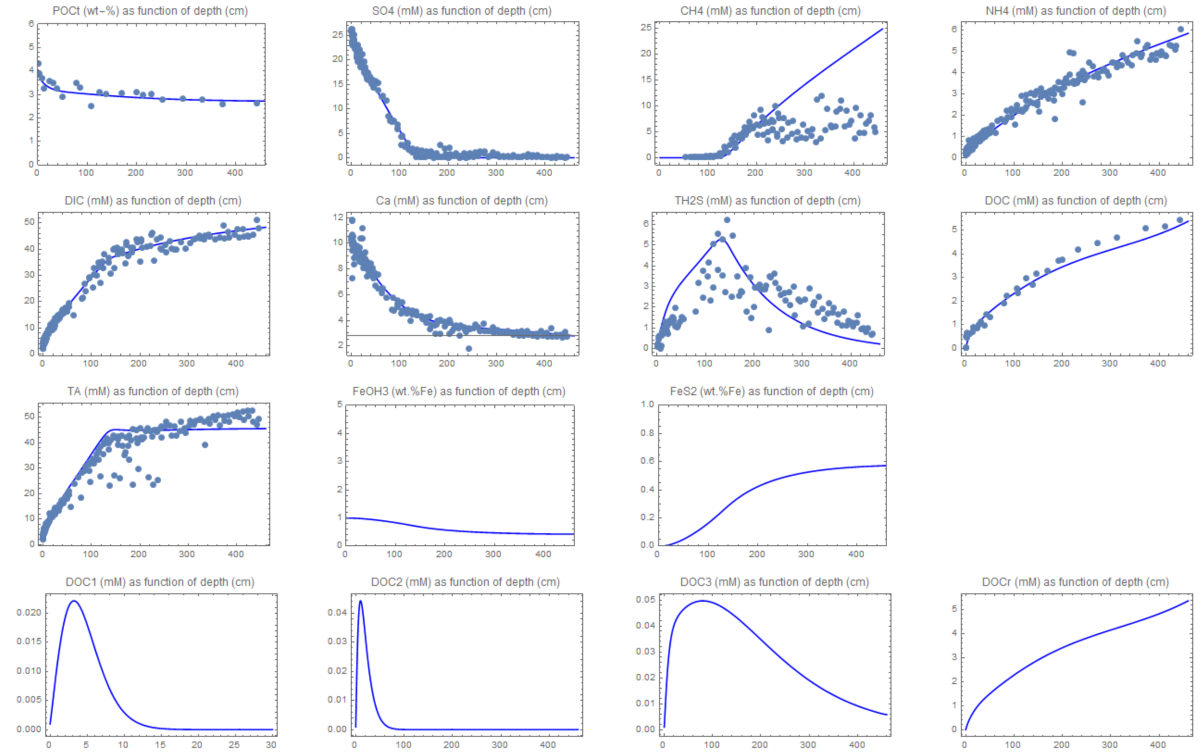

Figure S17. Profiles of modeled concentrations (vertical axis) versus depth (horizontal axis) with 3 DOC species based on the model of Refs.[4,19] and fitted against their field data[4,19] (solid lines are model curves and filled circles are measured field data). Here the DOC profile is the sum of DOC_1 to DOC_3 concentrations. This is for the investigation of increasing the number of DOC species and is to be compared with the following figure (Fig. S18).

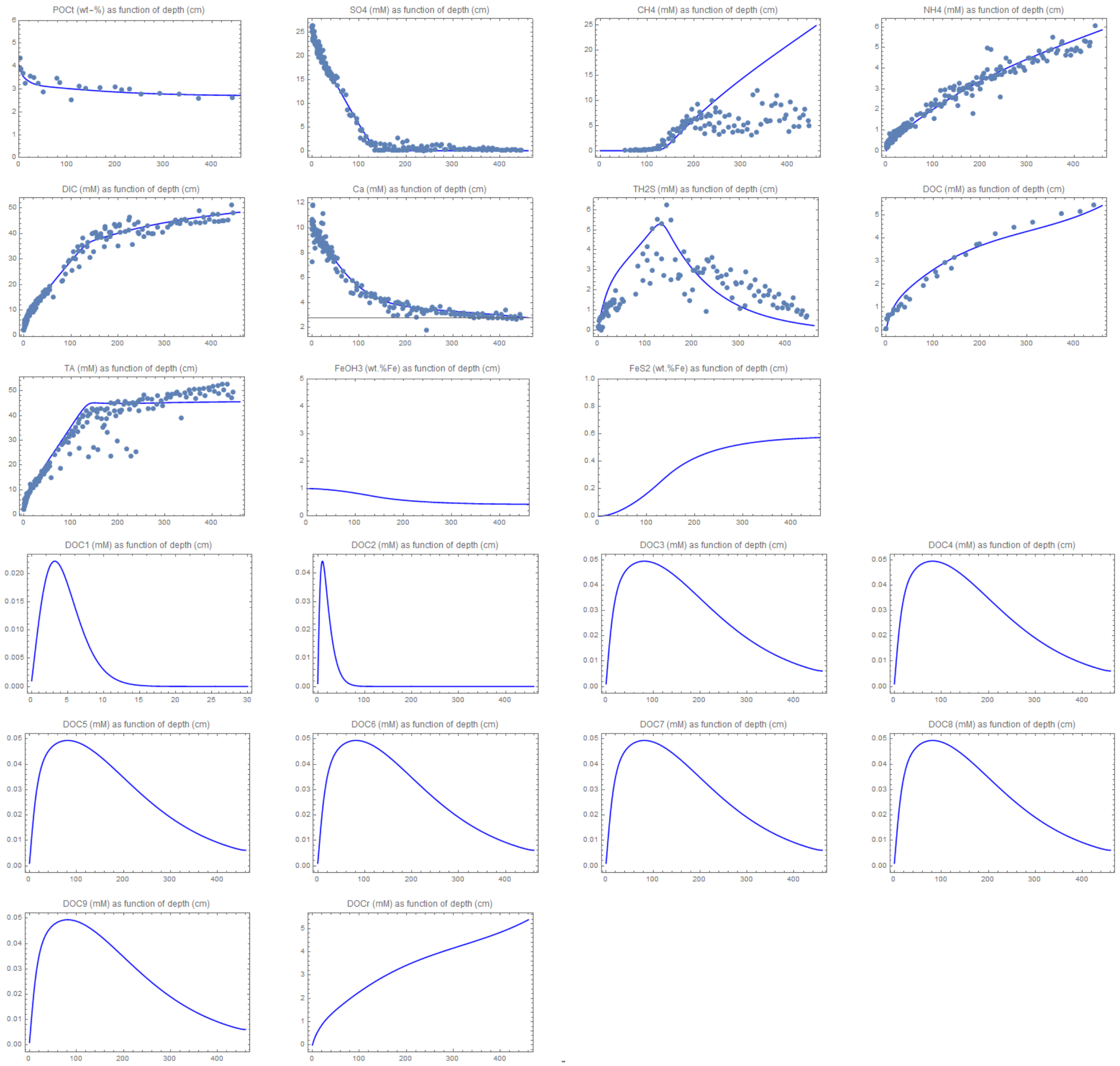

Figure S18. Profiles of modeled concentrations (vertical axis) versus depth (horizontal axis) with the addition of 6 new DOC species in the model used for generating the previous figure (Fig. S17) and fitted against the field data of Refs.[4,19] (solid lines are model curves and filled circles are measured field data). Here the DOC profile is the sum of DOC_1 to DOC_9 concentrations. This is for the investigation of increasing the number of DOC species and is to be compared with the previous figure (Fig. S17).

Table S1. Model parameters with their statistics, including upper and lower fences, the median and their selected ranges based on literature data presented in Table S2. The selected ranges are generally based on upper and lower fences of literature data. If the fences fall outside the physicochemically meaningful values, we select the meaningful values as the borders, e.g., for negative rate quantities, a value close to zero has been assumed (10^{-15}) instead.

Parameter	Literat. data lower fence	Literat. data upper fence	Literat. data median	Selected min	Selected max	Selected mean for probability distribution or fixed value	Note on selected values
L	-34.4	120.6	30.0			100	assumed sufficient to include most of the major biogeochemical processes
SFD**				0.02	7475.0		described in the footnote
P_0**	0.693	1.102	0.900	0.39	0.991		described in the footnote
P_f	0.557	0.931	0.745				assumed P0-0.18
p_x	-0.067	0.447	0.200			0.1814	average of the literature data (after excluding an outlier) instead of the median
u_0**				1.0E-05	3.3		described in the footnote
ds	2.2	2.9	2.6			2.5	assumed following [18]
Temp	268	294	281			278.15	
Sal*	-9.4	61.0	32.3	1.0E-12	61.0	32.3	
Press	-85.0	144.6	9.0			22.87	assumed
rNC	0.035	0.211	0.124			0.151	assumed as the Redfield ratio following [18]
D_{Bio0}*	-31.6	53.4	6.7	1.0E-15	53.4	6.7	
X_{Bio}*	-12.7	26.5	4.0	1.0E-15	11.8	4.0	
α_0*	-52.5	87.5	3.0	1.0E-15	87.5	3.0	
X_{BI}*	-21.4	41.6	5.0	1.0E-15	7.0	3.5	assumed following Ref.[99]

Parameter	Literat. data lower fence	Literat. data upper fence	Literat. data median	Selected min	Selected max	Selected mean for probability distribution or fixed value	Note on selected values
RR_{POC}	-0.034	0.060	0.005				Calculated from TOC (or POC_0) and MAR following[69] to convert constant concentration of POC at the surface to flux
POC_0**				1.0E-15	5.0		described in the footnote
MAR							not used in the main modeling; can be calculated from u_0 following[69]
k_{O2NH4}*	-37025	76967	10000	1.0E-15	76967	10000	
k_{O2TH2S}*	-1183385	1972735	160	1.0E-15	16000	160	
k_{Fe2O2} *	-12031570	2.0E7	140000	1.0E-15	10000000	100000	
k_{FeSO2}*	-14064	23826	300	1.0E-15	23826	300	
k_{FeS2O2}*	-125	210	1.0	1.0E-15	210	1.00	
k_{Fe2H2S}*	-290314	485323	14800	1.0E-15	485323	14800	
k_{FeSH2S}*	-77.1	128.9	0.2	1.0E-15	128.9	0.237	
k_{FeSS0}*	-6.3	18.9	7.0	1.0E-15	18.9	7.0	
$k_{Fe(OH)3HRH2S}$*	-486	814	8.0	1.0E-15	814.2	8.0	
$k_{Fe(OH)3MRH2S}$*	-12.6	31.6	8.0	1.0E-15	31.6	8.0	
$k_{Fe(OH)3HR>Fe(OH)3MR}$*	-0.87	1.52	0.58	1.0E-15	1.0	0.01	
k_{Mn2O2}*	-363339	612695	20000	1.0E-15	612695	20000	

Parameter	Literat. data lower fence	Literat. data upper fence	Literat. data median	Selected min	Selected max	Selected mean for probability distribution or fixed value	Note on selected values
$k_{MnO2HRFe2}$*	-2650	4421	2.0	1.0E-15	1000	10	
$k_{MnO2HRH2S}$*	-92.0	206.7	20.0	1.0E-15	206.7	100	
k_{S0}*	-25.6	50.7	3.0	1.0E-15	50.7	3.0	
K_{O2DOC}	-2.6E-02	4.8E-02	2.0E-02			0.001	following Ref.[18]
K_{NO3DOC}	-2.3E-03	1.2E-02	4.0E-03			0.01	following Ref.[18]
K_{SO4DOC}	-1.2	3.3	1.3			0.5	following Ref.[18]
$K_{MnO2HRDOC}$	-7.7E-02	1.3E-01	8.8E-04			0.1	following Ref.[18]
$K_{Fe(OH)3HRDOC}$	-0.45	0.75	3.6E-03			0.6	following Ref.[18]
k_{SO4CH4} (k_{AOM})*				0.498	49.8	4.98	following Ref.[4]; the ranges are assumed from 10 times less to 10 times more than the selected value
K_{SO4CH4} (K_a)			1.0			1.0	following [4]
$RR_{Fe(OH)3HR}$/$RR_{Fe(OH)3}$	-1.4E-03	2.5E-03	3.2E-04			0.5	assumed; and $RR_{Fe(OH)3} = 0.05u_0.(1-P_0).d_s$ following [18]
$RR_{Fe(OH)3MR}$/ $RR_{Fe(OH)3}$	-6.9E-04	1.1E-03	1.7E-04			0.5	assumed
RR_{MnO2HR}/ $RR_{Fe(OH)3}$	-2.9E-04	4.9E-04	2.7E-05			1	assumed
Kd_{fe}*	-548.9	1129.3	384.0	1.0E-15	40000	400	
Kd_{Mn}*	-5.8	44.4	15.6	1.0E-15	44.4	15.6	
Kd_{NH4}*			2.6	1.0E-15	26.4	2.6	

Parameter	Literat. data lower fence	Literat. data upper fence	Literat. data median	Selected min	Selected max	Selected mean for probability distribution or fixed value	Note on selected values
BW_{O2}**	-0.4	6.6E-01	1.2E-01	1.0E-15	0.40 $\rho_s.\varphi.\varepsilon^{-1}$		described in the footnote; multiplication is for converting the unit from $\mu mol.g^{-1}$ to $\mu mol.cm^{-3}$
BW_{NO3}**	0.0	7.2E-02	1.9E-02	1.0E-15	0.06 $\rho_s.\varphi.\varepsilon^{-1}$		described in the footnote; multiplication is for converting the unit from $\mu mol.g^{-1}$ to $\mu mol.cm^{-3}$
BW_{NH4}	-0.1	9.4E-02	2.3E-03			2.3E-03	
BW_{Mn2}	0.0	0.0	0.0			0.0	
BW_{Fe2}	0.0	0.0	0.0			0.0	
BW_{SO4}	16.8	35.1	27.7			27.7	
BW_{H2S}	0.0	0.0	0.0			0.0	
BW_{DOC}			0.0			0.0	assumed zero
BW_{lrDOC}			0.07			0.0	assumed zero
k_{DOC1}*			119.6	0.20	2.27	0.67	the mean was assumed to be equal to 1/lifetime of semi-labile DOC after [23], the min was assumed 1/3.4 times the mean and the max was assumed 3.4 times the mean initially assumed (0.67), so that $\sqrt{min \times max} \approx mean$
k_{DOC2}*	-47336	78894	33.7	2.27	25.95	7.67	the mean was assumed to be equal to 11.45 times the mean of k_{DOC1} (based on interpolation between DOC_1 and DOC_4), the min was

Parameter	Literat. data lower fence	Literat. data upper fence	Literat. data median	Selected min	Selected max	Selected mean for probability distribution or fixed value	Note on selected values
							assumed to be equal to the max of k_{DOC1} and the max was assumed 3.4 times the mean initially assumed (7.63), so that $\sqrt{min \times max} \approx mean$
k_{DOC3}*	-5.1	8.5	0.2	25.95	297.17	87.82	the mean was assumed to be equal to 11.45 times the mean of k_{DOC2} (based on interpolation between DOC_1 and DOC_4), the min was assumed to be equal to the max of k_{DOC2} and the max was assumed 3.4 times the mean initially assumed (87.40), so that $\sqrt{min \times max} \approx mean$
k_{DOC4}*			123	297.2	3400	1005	the mean was assumed to be equal to 1/lifetime of labile DOC after Ref. [23], the min was assumed to be equal to the max of k_{DOC3} and the max was assumed 3.4 times the mean initially assumed (1000), so that $\sqrt{min \times max} \approx mean$
k_{GPS1}*			33.7	1.17E-02	0.21	4.98E-02	the mean was assumed to be equal to 1/lifetime of semi-refractory DOC after Ref.[23], the min was assumed to be equal to the max of k_{GPS2} and the max was assumed 4.25 times the mean initially assumed (5.00E-02), so that $\sqrt{min \times max} \approx mean$

Parameter	Literat. data lower fence	Literat. data upper fence	Literat. data median	Selected min	Selected max	Selected mean for probability distribution or fixed value	Note on selected values
k_{GPS2}*			3.4	2.66E-04	1.17E-02	1.76E-03	the mean was assumed based on interpolation between GPS_1 and lrDOC, the min was assumed to be equal to the max of k_{lrDOC} and the max was assumed 4.25 times the mean initially assumed (2.75E-03), so that $\sqrt{min \times max} \approx mean$
k_{GPS3} or k_{rDOC} *			0.0	1.47E-05	2.66E-04	6.25E-05	the mean was assumed to be equal to 1/lifetime of refractory DOC after[23], the min was assumed to be equal to 1/4.25 times the mean initially assumed (6.25E-05), and the max was assumed 4.25 times the mean initially assumed, so that $\sqrt{min \times max} \approx mean$
Kd_{DOC1}*			0.0	1.0E-15	14.5	1.45	
Kd_{DOC2}*			0.0	1.0E-15	14.5	1.45	
Kd_{DOC3}*			0.0	1.0E-15	14.5	1.45	
Kd_{DOC4}*			1.0	1.0E-15	14.5	1.45	
Kd_{GPS1}*			0.7	1.0E-15	14.5	1.45	
Kd_{GPS2}*			2.9	1.0E-15	14.5	1.45	
Kd_{lrDOC}*			1.1	1.0E-15	14.5	1.45	
$ksorp_{DOC1}$*			0.0	1.0E-15	92.5	10.3	
$ksorp_{DOC2}$*			0.0	1.0E-15	92.5	10.3	

Parameter	Literat. data lower fence	Literat. data upper fence	Literat. data median	Selected min	Selected max	Selected mean for probability distribution or fixed value	Note on selected values
$ksorp_{DOC3}$*			0.0	1.0E-15	92.5	10.3	
$ksorp_{DOC4}$*			0.0	1.0E-15	92.5	10.3	
$ksorp_{GPS1}$*			18.5	1.0E-15	92.5	10.3	
$ksorp_{GPS2}$*			2.0	1.0E-15	92.5	10.3	
$ksorp_{lrDOC}$*			0.0	1.0E-15	92.5	10.3	
$Kdsorp_{DOC1}$ *			0.0	1.0E-15	367198	36720	Random sampling was conducted for $Ksorp_{DOC1}.Kdsorp_{DOCi}^{-1}.\rho_s^{-1}$ in range of 10^{-15} to 9.7×10^{-4} with a mean of 1.1×10^{-4}
$Kdsorp_{DOC2}$ *			0.0	1.0E-15	37973	36720	as above
$Kdsorp_{DOC3}$ *			0.0	1.0E-15	37973	36720	as above
$Kdsorp_{DOC4}$ *			0.0	1.0E-15	37973	36720	as above
$Kdsorp_{GPS1}$ *			0.0	1.0E-15	37973	36720	as above
$Kdsorp_{GPS2}$ *			0.0	1.0E-15	37973	36720	as above
$Kdsorp_{lrDOC}$ *			0.0	1.0E-15	37973	36720	as above
λ_{DOC1r} *			3.5	1.0E-15	17.5	3.5	
λ_{DOC1}*			187	88.7	933	187	
λ_{DOC2}*			187	88.7	933	187	
λ_{DOC3}*			187	88.7	933	187	

Parameter	Literat. data lower fence	Literat. data upper fence	Literat. data median	Selected min	Selected max	Selected mean for probability distribution or fixed value	Note on selected values
λ_{DOC4}*			187	88.7	933	187	
λ_{GPS1}*			17.7	4.53	88.7	17.7	
λ_{GPS2}*			0.9	1.0E-15	4.53	0.91	
v_{Gam}*				0.052	1.08	0.423	after [34,100]
a_{Gam}*				3.0E-04	35000	1.5	after [34,100]

*These parameters were randomly varied at the Monte Carlo stage of process importance analysis based on ranges that were selected mostly from the literature.

**These parameters were also randomly varied at the Monte Carlo stage of process importance analysis based on ranges taken from six global grid datasets and their statistical probability distributions used in the random sampling were obtained by direct fitting of different distributions to the histogram of their global grid data and selecting the best distributions (Figs. S1, S2).

Table S2. Data for model parameters compiled from 10 previous studies given in different columns of the Table. The citations to the mentioned references on the top row have been listed in the footnote. The descriptions of parameters and their units are given in Table S1.

Parameter	Dale et al., 2015 (adapted)	Berg et al., 2003	Fossing et al., 2004	Wijsman et al., 2002	Kraal et al., 2012	Reed et al., 2011a	Reed et al., 2011b	Burdige et al., 2016a,b	Meysman et al., 2003	Kasih et al., 2009
L	40	20	20	30	25	50	30	460	100	30
P_0	0.900	0.838	0.849	0.950	0.860		0.943	0.99	0.948	0.9
P_f	0.700	0.631	0.763	0.730	0.760	0.7		0.78	0.824	0.69
p_x	0.200	1.020	0.216	0.230	0.151		0.189	0.100	0.278	0.088
u_0										
ds	2.65	2.41	2.04	2.55	2.65	2.65	2.65	2.65	2.6	2.5
Temp	281.0	272.2	282.2	279.0	286.2	286.2	281.7	276.7	279.0	
Sal	35	32.3	23.2	17			11	34.6	34.2	
Press	9							1.12	58.5	
rNC	0.151	0.097	0.1	0.128	0.105			0.122	0.151	0.125
D_{Bio0}	50	145.165	11.9	12.053	0.36	*5*	5.411	0	0	8
X_{Bio}	5	4	11.8	1	3.5	2				11.8
α_0	20	48			40	0	0	0	0	6
X_{BI}	5	16		1	3.5					20
RR_{POC}	0.002	0.022	0.005	0.025	0.000		0.122		0.001	0.004
k_{O2NH4}	30	1.99E+04	7.89E+04		1.00E+04	3.90E+04	1.00E+04		5000	7889
k_{O2TH2S}	500	160.944	1.58E+06	1.60E+02	160	160	160		160	1.58E+06
k_{Fe2O2}	5.00E+05	34713	1.58E+07	1.07E+05	1.40E+05	1.40E+05	1.40E+05		140	1.58E+07

Parameter	Dale et al., 2015 (adapted)	Berg et al., 2003	Fossing et al., 2004	Wijsman et al., 2002	Kraal et al., 2012	Reed et al., 2011a	Reed et al., 2011b	Burdige et al., 2016a,b	Meysman et al., 2003	Kasih et al., 2009
k_{FeSO2}	100	189.3	1.89E+04	3.00E+02	300	300	300		0	1.89E+04
k_{FeS2O2}	1	9.47	5.05E+02	1.60E+02	1	1	1		0	9.467
k_{Fe2H2S}	1000	14832	2.37E+04	3.65E+05	100	14800	100		12000	1.18E+06
k_{FeSH2S}	100		9.47E-02	3.25E+00					0.14	0.237
k_{FeSS0}	100	7.25825	7.89E-01		7	3.15	7			9.467
$k_{Fe(OH)3HRH2S}$	3.16	22.0903	6.31E+02	3.65E-02	8	8	8		0	6312
$k_{Fe(OH)3MRH2S}$	0.003	22.0903			8	8	8			
$k_{Fe(OH)3HR>Fe(OH)3MR}$	0.7	0.568037	2.84E-02		0.6		0.6			0.028
k_{Mn2O2}	5000	347.1	4.73E+05	5.00E+03	2.0E+04	2.0E+04	2.0E+04		0	4.73E+05
$k_{MnO2HRFe2}$	1.00E+04	1.18	5.36E+02	3.00E+03	2	2	2		0	53.6
$k_{MnO2HRH2S}$	100	31.56	9.47E+01	2.00E+01	20	20	20		0.172	94.7
k_{S0}	1	3.16	2.21E+01		3	3	3			22.1
K_{O2POC}	0.001	0.020	0.020	0.001	0.020	0.020	0.020	1.0E+06	0.002	0.020
K_{NO3POC}	0.010	0.002	0.005	0.008	0.004	0.004	0.004	1.0E+06	0.002	0.005
K_{SO4POC}	0.5	0.5		1	1.6	1.6	1.6	0.5	1.6	
$K_{MnO2HRPOC}$	0.1	0.001	0.003	5	0.000	0.000	0.000	1.0E+06	0.001	0.003
$K_{Fe(OH)3HRPOC}$	0.6	0.001	0.006	12.5	0.004	0.002	0.004	1.0E+06	0.001	0.006
k_{SO4CH4} (k_{AOM})	1.00E+05							4.98		
$RR_{Fe(OH)3HR}$/ $RR_{Fe(OH)3}$	1.74E-04	4.58E-04	1.80E-04	5.71E-04	2.06E-06		4.43E-05		2.01E-03	1.22E-03
$RR_{Fe(OH)3MR}$/ $RR_{Fe(OH)3}$	1.74E-04	4.58E-04	1.80E-04		0		4.43E-05		0	2.45E-03

**Parameter**	**Dale et al., 2015 (adapted)**	**Berg et al., 2003**	**Fossing et al., 2004**	**Wijsman et al., 2002**	**Kraal et al., 2012**	**Reed et al., 2011a**	**Reed et al., 2011b**	**Burdige et al., 2016a,b**	**Meysman et al., 2003**	**Kasih et al., 2009**
RR_{MnO2HR}/ $RR_{Fe(OH)3}$	1.99E-04	1.30E-06	3.03E-06	2.01E-04	2.75E-05				1.10E-04	1.34E-05
Kd_{fe}		268	500						17.9	500
Kd_{Mn}		28	13						18.2	13
Kd_{NH4}								3.78		1.5
BW_{O2}	0.25			0.295	0.014				0.010	0.115
BW_{NO3}	1.90E-02			0.005	0.036				0.025	0.001
BW_{NH4}	1.00E-03				0.003			0.002		0.050
BW_{Mn2}	0			0	0				0	0
BW_{Fe2}	0			0	0				0	0
BW_{SO4}	28			1.60E+01	29			26.2	28	27.3
BW_{H2S}	0			0						0
BW_{DOC}								0.001		
BW_{lrDOC}								0.068		
k_{DOC1}								239.2		
k_{DOC2}								33.7		31558
k_{DOC}								3.40		0.158
k_{DOC4}								1.60E-04		

References citations are as follows: Dale et al.[18], Berg et al.[48], Fossing et al.[49], Wijsman et al.[51], Kraal et al.[50], Reed et al.[52], Reed et al.[53], Burdige et al.[4,19], Meysman et al.[54], Kasih et al.[55].

Table S3. List of model parameters in Kraal et al.[50] and calibrated values in fitting the basic model of the present study to their field data.

Parameter	Description	Unit	Parameter values from Kraal et al.[50]	Parameter values assumed or calibrated in fitting data of Kraal et al.
L	Depth of modeled sediments	cm	25	25
P_0	Porosity at the sediment-water interface	[-]	0.86	0.86
P_f	Porosity after compaction	[-]	0.76	0.76
p_x	Parameter for the exponential decrease in porosity with depth	cm^{-1}	0.15083	0.15083
u_f	Burial velocity of solids after compaction	$cm.yr^{-1}$	0.004	0.004
ds	Density of dry solids	$g.cm^{-3}$	2.65	2.65
Temp	Sediment-water interface temperature	K	276.5 – 286.8	281.7
Sal	Salinity	PSU	8 – 14	11
Press	Pressure	bar	NG	9
rNC	Molar N:C ratio during POM degradation	[-]	0.105	0.105
D_{Bio0}	Bioturbation coefficient at the sediment-water interface	$cm^2.yr^{-1}$	0.36	3.51
X_{Bio}	Halving depth of the bioturbated zone	cm	3.5	3.5
α_0	Bio-irrigation coefficient	yr^{-1}	10 (except 70 for O_2 and 0 for Fe^{2+})	10 (except 0 for Fe^{2+})
X_{BI}	Depth of bio-irrigated zone	cm	3.5	3.5
RR_{POC}	POC rain rate for POC_1, POC_2 and POC_3	$g.cm^{-2}.yr^{-1}$	2.16×10^{-4}, 5.10×10^{-5}, 2.04×10^{-5}	3.54×10^{-4}, 8.36×10^{-5}, 3.35×10^{-5}
k_{O2NH4}	Kinetic constant for aerobic ammonium oxidation	$mM^{-1}.yr^{-1}$	10^4	945864
k_{O2TH2S}	Kinetic constant for aerobic H_2S oxidation in $mM^{-1}.yr^{-1}$	$mM^{-1}.yr^{-1}$	160	160

Parameter	Description	Unit	Parameter values from Kraal et al.[50]	Parameter values assumed or calibrated in fitting data of Kraal et al.
$k_{NO3TH2S}$	Kinetic constant for anaerobic TH2S oxidation in $mM^{-1}.yr^{-1}$	$mM^{-1}.yr^{-1}$	NG	4.99
k_{Fe2O2}	Rate constant for aerobic oxidation of Fe^{2+}	$mM^{-1}.yr^{-1}$	1.4×10^{5}	290.6
k_{Fe2NO3}	Rate constant for anaerobic oxidation of Fe^{2+}	$mM^{-1}.yr^{-1}$	NG	100
k_{FeSO2}	Rate constant for aerobic oxidation of FeS	$mM^{-1}.yr^{-1}$	300	300
k_{FeS2O2}	Rate constant for aerobic oxidation of FeS_2	$mM^{-1}.yr^{-1}$	1	3.05×10^{-4}
k_{Fe2H2S}	Rate constant for FeS precipitation	$mM^{-1}.yr^{-1}$	100	27
k_{FeSH2S}	Rate constant for FeS_2 precipitation	$mM^{-1}.yr^{-1}$	NG	1.34×10^{-7}
k_{FeSS0}	Rate constant for FeS_2 precipitation	$mM^{-1}.yr^{-1}$	7	5.65×10^{-4}
$k_{Fe(OH)3HRH2S}$	Rate constant for $Fe(OH)_3^{HR}$ reduction by H_2S	$mM^{-0.5}.yr^{-1}$	8	70.4
$k_{Fe(OH)3MRH2S}$	Rate constant for $Fe(OH)_3^{MR}$ reduction by H_2S	$mM^{-0.5}.yr^{-1}$	8	0.017
$k_{Fe(OH)3HR>Fe(OH)3MR}$	Rate constant for $Fe(OH)_3^{HR}$ ageing to $Fe(OH)_3^{MR}$	yr^{-1}	0.6	0.81
k_{Mn2O2}	Rate constant for aerobic oxidation of Mn^{2+}	$mM^{-1}.yr^{-1}$	20000	1875
$k_{MnO2HRFe2}$	Rate constant for MnO_2MR reduction by Fe^{2+}	$mM^{-1}.yr^{-1}$	2	0
$k_{MnO2HRH2S}$	Rate constant for MnO_2HR reduction by H_2S	$mM^{-1}.yr^{-1}$	20	0
$k_{MnO2MRH2S}$	Rate constant for MnO_2HR reduction by H_2S	$mM^{-1}.yr^{-1}$	20	0
$k_{MnO2HR>MnO2MR}$	Rate constant for MnO_2^{HR} aging to MnO_2^{MR}	yr^{-1}	1.8	0

Parameter	Description	Unit	Parameter values from Kraal et al.[50]	Parameter values assumed or calibrated in fitting data of Kraal et al.
k_{S0}	Rate constant for S^0 degradation	yr^{-1}	3	3
K_{O2POC}	Monod constant for aerobic POC degradation	mM	0.02	5.51×10^{-3}
K_{O2DOC}	Monod constant for aerobic DOC degradation	mM	NG	5.51×10^{-3}
K_{NO3POC}	Monod constant for denitrification for POC	mM	0.004	1.26×10^{-2}
K_{NO3DOC}	Monod constant for denitrification for DOC	mM	NG	1.26×10^{-2}
K_{SO4POC}	Monod constant for sulfate reduction for POC	mM	1.6	1.6
K_{SO4DOC}	Monod constant for sulfate reduction for DOC	mM	NG	1.6
$K_{MnO2HRPOC}$	Monod constant (Half-saturation constant) for MnO_2HR reaction with POC	$g.g^{-1}$	2.20×10^{-4}	8.35×10^{-4}
$K_{MnO2HRDOC}$	Monod constant (Half-saturation constant) for MnO_2HR reaction with POC	$g.g^{-1}$	NG	8.35×10^{-4}
$K_{Fe(OH)3HRPOC}$	Monod constant (Half-saturation constant) for $Fe(OH)_3^{HR}$ reaction with DOC	$g.g^{-1}$	3.63×10^{-3}	1.39×10^{-4}
$K_{Fe(OH)3HRDOC}$	Monod constant (Half-saturation constant) for $Fe(OH)_3^{HR}$ reaction with DOC	$g.g^{-1}$	NG	1.39×10^{-4}
$RR_{Fe(OH)3HR}$	Rain rate of $Fe(OH)_3^{HR}$ to the seabed	$g.cm^{-2}.yr^{-1}$	2.07×10^{-6}	4.43×10^{-5}
$RR_{Fe(OH)3MR}$	Rain rate of $Fe(OH)_3^{MR}$ to the seabed	$g.cm^{-2}.yr^{-1}$	0	6.81×10^{-5}
RR_{MnO2HR}	Rain rate of $MnO_2^{HR}$to the seabed	$g.cm^{-2}.yr^{-1}$	2.75×10^{-5}	2.75×10^{-5}

Parameter	Description	Unit	Parameter values from Kraal et al.[50]	Parameter values assumed or calibrated in fitting data of Kraal et al.
RR_{MnO2MR}	Rain rate of MnO_2^{MR} to the seabed	$g.cm^{-2}.yr^{-1}$	0	5.50×10^{-7}
BW_{O2}	Oxygen concentration at the sediment-water interface	mM	0.014	0.014
BW_{NO3}	Nitrate concentration at the sediment-water interface	mM	0.036	0.036
BW_{NH4}	Ammonia concentration at the sediment-water interface	mM	0.0026	0.0026
BW_{Mn2}	Manganese concentration at the sediment-water interface	mM	0	0
BW_{Fe2}	Ferrous iron concentration at the sediment-water interface	mM	0	0
BW_{SO4}	Sulfate concentration at the sediment-water interface	mM	29	29
BW_{H2S}	Hydrogen sulfide concentration at the sediment-water interface	mM	NG	0
BW_{DIC}	DIC concentration at the sediment-water interface	mM	NG	2.17
$K_{POCfast}$	First-order degradation constant for POC_{fast}	yr^{-1}	0.25	1.23
$K_{POCslow}$	First-order degradation constant for POC_{slow}	yr^{-1}	1.5×10^{-3}	3.5×10^{-4}

NG: Not given.

Table S4. Goodness-of-fit criteria, including determination coefficient (R^2) and Nash–Sutcliff model efficiency (NSE) for model fits to the field data of Kraal et al.[50] and comparison with their model fits. These are conducted using the basic model (without DOC) and the model with a simple consideration of DOC. Here the determination coefficient was used as the objective function in the automatic calibration process.

	**Without DOC**		**Without DOC**		**With DOC**	
	$\boldsymbol{R^2}$		**NSE**		$\boldsymbol{R^2}$	**NSE**
	Present study	Kraal et al.	Present study	Kraal et al.	Present study	Present study
**POC_1**	0.833	0.711	0.802	0.665	0.834	0.802
**O_2**	0.499	0.477	0.195	-0.068	0.500	0.175
**NO_3**	0.989	0.996	0.966	0.995	0.990	0.968
**SO_4**	0.254	0.000	-0.357	-1.188	0.253	-0.431
**NH_4**	0.940	0.924	0.890	0.922	0.941	0.901
**Fe_2**	0.764	0.875	0.517	0.479	0.781	0.422
**Mn_2**	0.717	0.825	0.648	0.405	0.721	0.621
**$Fe(OH)_3^{HR}$**	0.913		0.857		0.909	0.822
**MnO_2^{HR}**	0.878	0.860	-0.188	0.831	0.875	-0.183
**FeS_2**	0.832	0.850	-61.032	-4.614	0.831	-53.642
**Mean**	0.762	0.724	-5.077	-0.175	0.764	-4.954

Table S5. Goodness-of-fit criteria, including determination coefficient (R^2) and Nash–Sutcliff model efficiency (NSE) for model fits to the field data of Kraal et al.[50] and comparison with their model fits. These are conducted using the basic model (without DOC) and the model with a simple consideration of DOC. Here the Nash–Sutcliff model efficiency was used as the objective function in the automatic calibration process.

	**Without DOC**		**Without DOC**	
	R^2		**NSE**	
	Present study	Kraal et al.	Present study	Kraal et al.
**POC_1**	0.833	0.711	0.802	0.665
**O_2**	0.494	0.477	0.339	-0.068
**NO_3**	0.980	0.996	0.976	0.995
**SO_4**	0.260	0.000	-0.347	-1.188
**NH_4**	0.935	0.924	0.854	0.922
**Fe_2**	0.749	0.875	0.669	0.479
**Mn_2**	0.739	0.825	0.659	0.405
**$Fe(OH)_3HR$**	0.916		0.834	
**MnO_2HR**	0.794	0.860	-0.156	0.831
**FeS_2**	0.866	0.850	-1.308	-4.614
**Mean**	0.757	0.724	0.332	-0.175

Table S6. Summary of the results for step-by-step consideration of the proposed conceptual model towards including DOC and GPS species and considering all proposed processes (equilibrium adsorption, kinetic sorption, and geopolymerization) tested against field-modeling data of Refs.[4,19]. The units of parameters are given in Table S1.

**Parameters/ Indicators**	**Reproduction of Burdige et al. model**	**The proposed concept, including DOC cascaded hydrolysis**	**Including equilibrium adsorption**	**Including kinetic sorption**	**Including GPS and geo-polymerization**	**Conceptualizing the parameter trends**	**Adding link between DOC_1 and lrDOC***	**Further adjustment***
**Steps of the model development**	Step 1	Step 2	Step 3	Step 4	Step 5	Step 6	Step 7	Step 8
k_{DOC1}	NA	0	0	0	239.2	1.00E-05	1.00E-05	1.00E-05
k_{DOC2}	NA	0	0	0	33.7	5.00E-04	5.00E-04	5.00E-04
k_{DOC3}	NA	0	0	0	3.4	1.21E-02	1.21E-02	1.21E-02
k_{DOC4}	NA	0	0	1.035	1.60E-04	232.024	246.2	246.2
k_{GPS1}	NA	NA	NA	NA	1.60E-04	33.7	33.7	33.7
k_{GPS2}	NA	NA	NA	NA	1.60E-04	3.4102	3.41	3.41
K_{lrDOC}	NA	NA	NA	NA	1.60E-04	1.60E-04	9.95E-05	1.14E-04
Kd_{DOC1}	NA	0	0.9199	0	0	0	0	0
Kd_{DOC2}	NA	0	0.0156	0	0	0	0	0
Kd_{DOC3}	NA	0	0.0197	0	0	0	0	0
Kd_{DOC4}	NA	0	2.1250	0	1.035	1.035	1.04	1.04
Kd_{GPS1}	NA	NA	NA	NA	0.748	0.748	0.75	0.75
Kd_{GPS2}	NA	NA	NA	NA	2.892	2.892	2.89	2.89
Kd_{lrDOC}	NA	NA	NA	NA	1.116	1.116	1.14	1.14

**$ksorp_{DOC1}$**	NA	0	0	0	0	0	0	0
**$ksorp_{DOC2}$**	NA	0	0	5.872	5.87	0	0	0
**$ksorp_{DOC3}$**	NA	0	0	1.944	1.94	0	0	0
**$ksorp_{DOC4}$**	NA	0	0	0	0	0	0	0
**$ksorp_{GPS1}$**	NA	NA	NA	0	0	5.87	5.87	18.5
**$ksorp_{GPS2}$**	NA	NA	NA	0	0	1.94	1.94	2.00
**$ksorp_{lrDOC}$**	NA	NA	NA	0	0	0	0	0
**$Kdsorp_{DOC1}$**	NA	NA	NA	0	0	0	0	0
**$Kdsorp_{DOC2}$**	NA	NA	NA	1.22E+04	1.22E+04	0	0	0
**$Kdsorp_{DOC3}$**	NA	NA	NA	2.58E+04	2.58E+04	0	0	0
**$Kdsorp_{DOC4}$**	NA	NA	NA	0	0	0	0	0
**$Kdsorp_{GPS1}$**	NA	NA	NA	NA	0	1.22E+04	1.81E-04	3.58E+04
**$Kdsorp_{GPS2}$**	NA	NA	NA	NA	0	2.58E+04	2.85E-05	2.65E+04
**$Kdsorp_{lrDOC}$**	NA	NA	NA	NA	0	0	0	0
**λ_{DOC1}**	NA	150	177.45	186.7	186.69	186.69	186.69	186.69
**λ_{DOC2}**	NA	15.0	17.67	17.73	17.73	186.69	186.69	186.69
**λ_{DOC3}**	NA	1.50	1.900	1.81	1.81	186.69	186.69	186.69
**λ_{DOC4}**	NA	NA	NA	NA	5.00E-01	186.69	186.69	186.69
**λ_{GPS1}**	NA	NA	NA	NA	1.15E-01	17.73	17.73	17.73
**λ_{GPS2}**	NA	NA	NA	NA	3.00E-02	1.81	0.905	0.905
**λ_{DOC1r}**	NA	NA	NA	NA	0	0	3.62	3.494
**NSE averaged for all species****	0.4560	0.4683	0.4885	0.4491	0.5793	0.5818	0.5881	0.5222
**NSE for total DOC and GPS**	0.9716	0.9784	0.9672	0.9900	0.9904	0.9911	0.9896	0.9911
**MSC for all species****	NA	0.626	0.657	0.787	0.824	0.830	0.843	0.695
**MSC for total DOC and GPS**	3.560	3.628	2.934	4.055	3.684	3.276	3.051	3.208

**Number of parameters added**	0	3	7	8	21	21	22	22
**Mean mass balance error (%)**	0.02	0.01	0.02	0.06	0.08	0.09	0.12	0.12

NA: Not applicable

*Step 8 is a further adjustment of six of the parameters from Step 7 to get the best possible fit for the data of total DOC.

**In calculating the mean NSE, model fit results for total POC and total DOC+GPS+lrDOC were considered for each individual POC or DOC/GPS/lrDOC species, and thus the contributions of total POC and total DOC+GPS+lrDOC to the mean NSE have been multiplied by the number of their individual species. Consequently, the MSC of all species, which was calculated based on the mean NSE, was affected by total POC and total DOC+GPS in the same way. This results in a sudden increase in NSE and MSC from Step 4 on, where GPS species and lrDOC are included in the model, and therefore, the number of these species is increased.

Table S7. Nash–Sutcliff model efficiency (NSE) and the optimum number of nodes in the hidden layer of ANNs obtained during multiple runs of the process importance analysis through ANN fits to data generated from the Monte Carlo stage of the modeling procedure with 68 model parameters. The number of process importance analysis runs is 1000 in all cases.

NSE or the number of hidden layer nodes in the ANN	*PE* MOC & POC	(semi) labile DOC-MOC* preser vation	GPS-MOC preser vation	lrDOC-MOC preserv ation
**Mean NSE of the best fits**	0.9232	0.9261	0.9404	0.9444
**Min of NSE of the best fits**	0.9056	0.9031	0.9238	0.8975
**Max of NSE of the best fits**	0.9439	0.9487	0.9552	0.9613
**Mean number of nodes of the best fits**	20	13	13	6
**Min number of nodes of the best fits**	3	3	3	3
**Max number of nodes of the best fits**	36	36	36	36
**Corresponding node to the Max NSE**	17	6	11	4

PE: preservation efficiency (%), POC: particulate organic carbon; DOC: dissolved organic carbon; MOC: mineral-phase organic carbon; DOC-MOC: mineral phase DOC resulting from kinetic sorption of (semi)labile DOC to minerals; GPS-MOC: mineral phase GPS resulting from kinetic sorption of GPS to minerals; lrDOC-MOC: mineral phase lrDOC resulting from kinetic sorption of lrDOC to minerals.

*Note the (semi)labile DOC pool is comprised of both labile and semi-labile DOC fractions.

Table S8. List of model parameters, their descriptions, units, parameter grouping used in the process of process importance analysis and probability distributions used in Monte Carlo sampling.*

Parameter	Description	Unit	Parameter group	Probability distribution assumed in Monte Carlo sampling
L	Depth of modeled sediments	cm	-	
SFD	Water depth	m	-	Normal
P_0	Porosity at the sediment-water interface	[-]	-	Normal
P_f	Porosity after compaction	[-]	-	
p_x	Parameter for the exponential decrease in porosity with depth	cm^{-1}	-	
u_0	Burial velocity of solids before compaction	$cm.yr^{-1}$	-	Normal
ds	The density of dry solids	$g.cm^{-3}$	-	
Temp	Sediment-water interface temperature	K	-	
Sal	Salinity	PSU	-	Uniform
Press	Pressure	bar	-	
rNC	Molar N:C ratio during POM degradation	[-]	-	
D_{Bio0}	Bioturbation coefficient at the sediment-water interface	$cm^2.yr^{-1}$	Mixing	Uniform
X_{Bio}	Halving depth of the bioturbated zone	cm	Mixing	Uniform
α_0	Bio-irrigation coefficient	yr^{-1}	Mixing	Uniform
X_{BI}	Depth of bio-irrigated zone	cm	Mixing	Uniform
RR_{POC}	POC rain rate	$g.cm^{-2}.yr^{-1}$	-	
POC_0	Total organic carbon concentration	[%]	-	Lognormal
MAR	Sediment mass accumulation rate	$g.cm^{-2}.yr^{-1}$	-	

Parameter	Description	Unit	Parameter group	Probability distribution assumed in Monte Carlo sampling
k_{O2NH4}	Kinetic constant for aerobic ammonium oxidation	$mM^{-1}.yr^{-1}$	-	Uniform
k_{O2TH2S}	Kinetic constant for aerobic H_2S oxidation in $mM^{-1}.yr^{-1}$	$mM^{-1}.yr^{-1}$	-	Uniform
k_{Fe2O2}	Rate constant for aerobic oxidation of Fe^{2+}	$mM^{-1}.yr^{-1}$	-	Uniform
k_{FeSO2}	Rate constant for aerobic oxidation of FeS	$mM^{-1}.yr^{-1}$	-	Uniform
k_{FeS2O2}	Rate constant for aerobic oxidation of FeS_2	$mM^{-1}.yr^{-1}$	-	Uniform
k_{Fe2H2S}	Rate constant for FeS precipitation	$mM^{-1}.yr^{-1}$	-	Uniform
k_{FeSH2S}	Rate constant for FeS_2 precipitation	$mM^{-1}.yr^{-1}$	-	Uniform
k_{FeSS0}	Rate constant for FeS_2 precipitation	$mM^{-1}.yr^{-1}$	-	Uniform
$k_{Fe(OH)3HRH2S}$	Rate constant for $Fe(OH)_3^{HR}$ reduction by H_2S	$mM^{-0.5}.yr^{-1}$	-	Uniform
$k_{Fe(OH)3MRH2S}$	Rate constant for $Fe(OH)_3^{MR}$ reduction by H_2S	$mM^{-0.5}.yr^{-1}$	-	Uniform
$k_{Fe(OH)3HR>Fe(OH)3MR}$	Rate constant for $Fe(OH)_3^{HR}$ ageing to $Fe(OH)_3^{MR}$	yr^{-1}	-	Uniform
k_{Mn2O2}	Rate constant for aerobic oxidation of Mn^{2+}	$mM^{-1}.yr^{-1}$	-	Uniform
$k_{MnO2HRFe2}$	Rate constant for MnO_2MR reduction by Fe^{2+}	$mM^{-1}.yr^{-1}$	-	Uniform
$k_{MnO2HRH2S}$	Rate constant for MnO_2HR reduction by H_2S	$mM^{-1}.yr^{-1}$	-	Uniform
k_{S0}	Rate constant for S^0 degradation	yr^{-1}	-	Uniform
K_{O2DOC}	Monod constant for aerobic DOC degradation	mM	-	

Parameter	Description	Unit	Parameter group	Probability distribution assumed in Monte Carlo sampling
K_{NO3DOC}	Monod constant for denitrification for DOC	mM	-	
K_{SO4DOC}	Monod constant for Sulfate reduction for DOC	mM	-	
$K_{MnO2HRDOC}$	Monod constant (Half-saturation constant) for MnO_2HR reaction with DOC	$g.g^{-1}$	-	
$K_{Fe(OH)3HRDOC}$	Monod constant (Half-saturation constant) for $Fe(OH)_3^{HR}$ reaction with DOC	$g.g^{-1}$	-	
k_{SO4CH4} (k_{AOM})		yr^{-1}	-	Uniform
K_{SO4CH4} (K_a)		mM	-	
$RR_{Fe(OH)3HR}/RR_{Fe(OH)3}$	Ratio of rain rate of $Fe(OH)_3^{HR}$ to the seabed to the total $Fe(OH)_3$ rain rate	[-]	-	
$RR_{Fe(OH)3MR}/ RR_{Fe(OH)3}$	Ratio of rain rate of $Fe(OH)_3^{MR}$ to the seabed to the total $Fe(OH)_3$ rain rate	[-]	-	
$RR_{MnO2HR}/ RR_{Fe(OH)3}$	Ratio of rain rate of MnO_2^{HR} to the seabed to the $Fe(OH)_3$ rain rate	[-]	-	
Kd_{fe}	Distribution coefficient for dissolved iron	$cm^3.g^{-1}$	-	Uniform
Kd_{Mn}	Distribution coefficient for dissolved Mn	$cm^3.g^{-1}$	-	Uniform
Kd_{NH4}	Distribution coefficient for NH_4	$cm^3.g^{-1}$	-	Uniform
BW_{O2}	Oxygen concentration at the sediment-water interface	mM	-	Normal
BW_{NO3}	Nitrate concentration at the sediment-water interface	mM	-	Normal
BW_{NH4}	Ammonia concentration at the sediment-water interface	mM	-	

Parameter	Description	Unit	Parameter group	Probability distribution assumed in Monte Carlo sampling
BW_{Mn2}	Manganese concentration at the sediment-water interface	mM	-	
BW_{Fe2}	Ferrous iron concentration at the sediment-water interface	mM	-	
BW_{SO4}	Sulfate concentration at the sediment-water interface	mM	-	
BW_{H2S}	Hydrogen sulfide concentration at the sediment-water interface	mM	-	
BW_{DOC}	DOC concentration at the sediment-water interface	mM	-	
BW_{lrDOC}	lrDOC concentration at the sediment-water interface	mM	-	
k_{DOC1}	First-order degradation constant for DOC_1	yr^{-1}	Remineralization	Uniform
k_{DOC2}	First-order degradation constant for DOC_2	yr^{-1}	Remineralization	Uniform
k_{DOC3}	First-order degradation constant for DOC_3	yr^{-1}	Remineralization	Uniform
k_{DOC4}	First-order degradation constant for DOC_4	yr^{-1}	Remineralization	Uniform
k_{GPS1}	First-order degradation constant for GPS_1	yr^{-1}	Remineralization	Uniform
k_{GPS2}	First-order degradation constant for GPS_2	yr^{-1}	Remineralization	Uniform
k_{lrDOC}	First-order degradation constant for GPS_3 (lrDOC)	yr^{-1}	Remineralization	Uniform
Kd_{DOC1}	Adsorption coefficient for DOC_1	$cm^3.g^{-1}$	Equilibrium adsorption	Uniform
Kd_{DOC2}	Adsorption coefficient for DOC_2	$cm^3.g^{-1}$	Equilibrium adsorption	Uniform
Kd_{DOC3}	Adsorption coefficient for DOC_3	$cm^3.g^{-1}$	Equilibrium adsorption	Uniform

Parameter	Description	Unit	Parameter group	Probability distribution assumed in Monte Carlo sampling
Kd_{DOC4}	Adsorption coefficient for DOC_4	$cm^3.g^{-1}$	Equilibrium adsorption	Uniform
Kd_{GPS1}	Adsorption coefficient for GPS_1	$cm^3.g^{-1}$	Equilibrium adsorption	Uniform
Kd_{GPS2}	Adsorption coefficient for GPS_2	$cm^3.g^{-1}$	Equilibrium adsorption	Uniform
Kd_{lrDOC}	Adsorption coefficient for lrDOC	$cm^3.g^{-1}$	Equilibrium adsorption	Uniform
$ksorp_{DOC1}$	Mass transfer rate between the dissolved and sorbed phases for DOC_1	yr^{-1}	Kinetic sorption	Uniform
$ksorp_{DOC2}$	Mass transfer rate between the dissolved and sorbed phases for DOC_2	yr^{-1}	Kinetic sorption	Uniform
$ksorp_{DOC3}$	Mass transfer rate between the dissolved and sorbed phases for DOC_3	yr^{-1}	Kinetic sorption	Uniform
$ksorp_{DOC4}$	Mass transfer rate between the dissolved and sorbed phases for DOC_4	yr^{-1}	Kinetic sorption	Uniform
$ksorp_{GPS1}$	Mass transfer rate between the dissolved and sorbed phases for GPS_1	yr^{-1}	Kinetic sorption	Uniform
$ksorp_{GPS2}$	Mass transfer rate between the dissolved and sorbed phases for GPS_2	yr^{-1}	Kinetic sorption	Uniform
$ksorp_{lrDOC}$	Mass transfer rate between the dissolved and sorbed phases for lrDOC	yr^{-1}	Kinetic sorption	Uniform
$Kdsorp_{DOC1}$	Equivalent distribution coefficient for kinetic sorption of DOC_1	$cm^3.g^{-1}$	Kinetic sorption	Uniform
$Kdsorp_{DOC2}$	Equivalent distribution coefficient for kinetic sorption of DOC_2	$cm^3.g^{-1}$	Kinetic sorption	Uniform
$Kdsorp_{DOC3}$	Equivalent distribution coefficient for kinetic sorption of DOC_3	$cm^3.g^{-1}$	Kinetic sorption	Uniform
$Kdsorp_{DOC4}$	Equivalent distribution coefficient for kinetic sorption of DOC_4	$cm^3.g^{-1}$	Kinetic sorption	Uniform
$Kdsorp_{GPS1}$	Equivalent distribution coefficient for kinetic sorption of GPS_1	$cm^3.g^{-1}$	Kinetic sorption	Uniform

Parameter	Description	Unit	Parameter group	Probability distribution assumed in Monte Carlo sampling
$Kdsorp_{GPS2}$	Equivalent distribution coefficient for kinetic sorption of GPS_2	$cm^3.g^{-1}$	Kinetic sorption	Uniform
$Kdsorp_{lrDOC}$	Equivalent distribution coefficient for kinetic sorption of lrDOC	$cm^3.g^{-1}$	Kinetic sorption	Uniform
λ_{DOC1r}	Conversion rate of DOC_1 to lrDOC	yr^{-1}	DOC hydrolysis	Uniform
λ_{DOC1}	Conversion rate of DOC_1 to DOC_2	yr^{-1}	DOC hydrolysis	Uniform
λ_{DOC2}	Conversion rate of DOC_2 to DOC_3	yr^{-1}	DOC hydrolysis	Uniform
λ_{DOC3}	Conversion rate of DOC_3 to DOC_4	yr^{-1}	DOC hydrolysis	Uniform
λ_{DOC4}	Conversion rate of DOC_4 to GPS_1	yr^{-1}	Geopolymerization	Uniform
λ_{GPS1}	Conversion rate of GPS_1 to GPS_2	yr^{-1}	Geopolymerization	Uniform
λ_{GPS2}	Conversion rate of GPS_2 to lrDOC	yr^{-1}	Geopolymerization	Uniform
v_{Gam}	Continuum model parameter, the average lifetime of reactive POC	[-]	-	Uniform
a_{Gam}	Continuum model parameter, determining the shape of the distribution near $k_{POC} = 0$	yr	-	Lognormal

*Parameters that do not have a distribution are not varied in the Monte Carlo process or the rest of the modeling process.

Table S9. Summary of model parameter grouping into three previously known processes and three proposed processes in this study.

**Known or proposed**	**Parameter groups/processes**	**Abbreviation/ names**	**Parameters in each group**
Previously known process	DOC hydrolysis	DOC hydrolysis	λ_{DOC1r}, λ_{DOC1}, λ_{DOC2}, λ_{DOC3}
	DOC and GPS remineralization	Remineralis.	k_{DOC1}, k_{DOC2}, k_{DOC3}, k_{DOC4}, k_{GPS1}, k_{GPS2}, k_{lrDOC}
	Sediment mixing by fauna	Mixing	X_{BI}, α_0, X_{Bio}, D_{Bio0}
Proposed added processes	Equilibrium adsorption	Equilib. Adsorp.	Kd_{DOC1}, Kd_{DOC2}, Kd_{DOC3}, Kd_{DOC4}, Kd_{GPS1}, Kd_{GPS2}, Kd_{lrDOC}
	Kinetic sorption	Kinetic Sorption	$ksorp_{DOC1}$, $ksorp_{DOC2}$, $ksorp_{DOC3}$, $ksorp_{DOC4}$, $ksorp_{GPS1}$, $ksorp_{GPS2}$, $ksorp_{lrDOC}$, $Kdsorp_{DOC1}$, $Kdsorp_{DOC2}$, $Kdsorp_{DOC3}$, $Kdsorp_{DOC4}$, $Kdsorp_{GPS1}$, $Kdsorp_{GPS2}$, $Kdsorp_{lrDOC}$
	Geopolymerization	Geopolymeri.	λ_{DOC4}, λ_{GPS1}, λ_{GPS2}

Table S10. List of primary and secondary redox reactions considered in the early diagenesis model with rate constants (adapted from Ref.[18].

Reaction number	Reaction name	Stoichiometry	Rate expression	Parameter(s)	Parameter unit(s)
1	DOC* degradation	$DOC + O_2 \rightarrow CO_2 + \frac{1}{(C:N)} NH_4^+ + H_2O$	$k_{DOCi}.[DOC_i]$	k_{DOCi}	yr^{-1}
2	DIC production via DOC degradation (DIC+CH_4)	$DOC \rightarrow 0.5CO_2 + \frac{1}{(C:N)} NH_4^+ + 0.5CH_4$	$\sum k_{DOCi}.[DOC_i]$		yr^{-1}
3	NH_4 release (ammonification) via DOC degradation	$DOC + 0.8NO_3^- + 0.8H^+ \rightarrow CO_2 + \frac{1}{(C:N)} NH_4^+ + 0.4N_2 + 1.4H_2O$	$rNC.k_{POCi}.[POC_i]$	rNC, k_{POCi}	[–], yr^{-1}
4	O_2 consumption via aerobic DOC degradation	$DOC + O_2 \rightarrow CO_2 + \frac{1}{(C:N)} NH_4^+ + H_2O$	$\sum k_{DOCi}.[DOC_i].[O_2] / (K_{O2DOC}+[O_2])$	K_{O2DOC}	mM
5	Denitrification	$DOC + 0.8NO_3^- + 0.8H^+ \rightarrow CO_2 + \frac{1}{(C:N)} NH_4^+ + 0.4N_2 + 1.4H_2O$	$\sum k_{DOCi}.[DOC_i].\ K_{O2DOC} / (K_{O2DOC}+[O_2]).[NO_3] / (K_{NO3DOC}+[NO_3])$	K_{NO3DOC}	mM
7	DOC degradation via Sulfate reduction	$DOC + 0.5SO_4^{2-} + H^+ \rightarrow CO_2 + \frac{1}{(C:N)} NH_4^+ + H_2O + \frac{1}{2} H_2S$	$L_2.\sum k_{DOCi}.[DOC_i].[SO_4] / (K_{SO4DOC}+[SO_4])$	K_{SO4DOC}	mM
8	Methanogenesis	$DOC \rightarrow 0.5CO_2 + \frac{1}{(C:N)} NH_4^+ + 0.5CH_4$	$L_2.\sum k_{DOCi}.[DOC_i].(1- [SO_4] / (K_{SO4DOC}+[SO_4]))$		
9	Aerobic NH_4 oxidation	$NH_4^+ + 2O_2 \rightarrow NO_3^- + H_2O + 2H^+$	$k_{O2NH4}.[O_2].[NH_4]$	k_{O2NH4}	$mM^{-1}.yr^{-1}$

Reaction number	Reaction name	Stoichiometry	Rate expression	Parameter(s)	Parameter unit(s)
10	Aerobic TH_2S oxidation	$H_2S + 2O_2 \rightarrow SO_4^{2-} + 2H^+$	$k_{O2H2S} \cdot [O_2] \cdot [H_2S]$	k_{O2H2S}	$mM^{-1}.yr^{-1}$
11	Aerobic CH_4 oxidation	$CH_4 + 2O_2 \rightarrow CO_2 + 2H_2O$	$k_{O2CH4} \cdot [O_2] \cdot [CH_4]$	k_{O2CH4}	$mM^{-1}.yr^{-1}$
12	Anaerobic CH_4 oxidation	$CH_4 + SO_4^{2-} + 2H^+ \rightarrow H_2S + CO_2 + 2H_2O$	$k_{SO4CH4} \cdot [SO_4].[CH_4]$ / $([SO_4] + K_{SO4CH4})$	k_{SO4CH4}, K_{SO4CH4}	yr^{-1}, mM
13	Anaerobic TH_2S oxidation	$H_2S + NO_3^- + H_2O \rightarrow SO_4^{2-} + NH_4^+$	$k_{NO3H2S} \cdot [NO_3] \cdot [H_2S]$	k_{NO3H2S}	$mM^{-1}.yr^{-1}$
14	Secondary redox reactions	$DOC + 2MnO_2 \rightarrow 2Mn^{2+} + CO_2 + \frac{1}{(C:N)}NH_4^+ + \frac{1}{(P:C)}PO_4^{3-}$	$2\sum k_{DOCi}.[DOC_i].\ K_{O2DOC}$ / $(K_{O2DOC}+[O_2]).\ K_{NO3DOC}$ / $(K_{NO3DOC}+[NO_3]).[MnO_2]$ / $(K_{MnO2DOC}+[MnO_2])$	$K_{MnO2DOC}$	mM
15		$DOC + 4FeOOH \rightarrow 4Fe^{2+} + CO_2 + \frac{1}{(C:N)}NH_4^+$	$4\sum k_{DOCi}.[DOC_i].\ K_{O2DOC}$ / $(K_{O2DOC}+[O_2]).\ K_{NO3DOC}$ / $(K_{NO3DOC}+[NO_3]).$ $K_{MnO2DOC}$ / $(K_{MnO2DOC}+[MnO_2]).$ $[Fe(OH)_3]$ / $(K_{Fe(OH)3DOC}+[Fe(OH)_3])$	$K_{Fe(OH)3DOC}$	mM
16		$Mn^{2+} + 0.5O_2 \rightarrow MnO_2$	$k_{Mn2O2}.[O_2].[Mn^{2+}]$	k_{Mn2O2}	$mM^{-1}.yr^{-1}$
17		$Fe^{2+} + 0.25O_2 \rightarrow FeOOH$	$k_{Fe2O2}.[O_2].[Fe^{2+}]$	k_{Fe2O2}	$mM^{-1}.yr^{-1}$
18		$Fe^{2+} + 0.2NO^{3-} \rightarrow FeOOH + 0.1N_2$	$k_{Fe2NO3}.[NO_3].[Fe^{2+}]$	k_{Fe2NO3}	$mM^{-1}.yr^{-1}$
19		$FeS + 2O_2 \rightarrow Fe^{2+} + SO4^{2-}$	$k_{FeSO2} \cdot [FeS] \cdot [O_2].$ $\rho_s.\varepsilon.(\varphi.AW_{FeS})^{-1}.10^6$	k_{FeSO2}	$mM^{-1}.yr^{-1}$
20		$FeS_2 + 3.5O_2 \rightarrow Fe^{2+} + 2SO_4^{2-}$	$k_{FeS2O2} \cdot [FeS_2] \cdot [O_2]$. $\rho_s.\varepsilon.(\varphi.AW_{FeS2})^{-1}.10^6$	k_{FeS2O2}	$mM^{-1}.yr^{-1}$
21		$Fe^{2+} + H_2S \rightarrow FeS$	$k_{Fe2H2S} \cdot [Fe^{2+}] \cdot [H_2S]$	k_{Fe2H2S}	$mM^{-1}.yr^{-1}$

Reaction number	Reaction name	Stoichiometry	Rate expression	Parameter(s)	Parameter unit(s)
22		$FeS + H_2S \rightarrow FeS_2 + H_2$	$k_{FeSH2S} \cdot [FeS] \cdot [H_2S]. \rho_s.\varepsilon.(\varphi.AW_{FeS})^{-1}.10^6$	k_{FeSH2S}	$mM^{-1}.yr^{-1}$
23		$FeS + S^0 \rightarrow FeS_2$	$k_{FeSS0} \cdot [FeS] \cdot [S^0]. \rho_s.\varepsilon.(\varphi.AW_{FeS})^{-1}.10^6 . \rho_s.\varepsilon.(\varphi.AW_{S0})^{-1}.10^6$	k_{FeSS0}	$mM^{-1}.yr^{-1}$
24		$MnO_2 + 2Fe^{2+} \rightarrow 2FeHR + Mn^{2+}$	$k_{MnO2Fe2} \cdot [MnO_2] \cdot [Fe^{2+}]. \rho_s.\varepsilon.(\varphi.AW_{Mn})^{-1}.10^6$	$k_{MnO2Fe2}$	$mM^{-1}.yr^{-1}$
25		$MnO_2 + H_2S \rightarrow S^0 + Mn^{2+}$	$k_{MnO2H2S} \cdot [MnO_2] \cdot [H_2S]. \rho_s.\varepsilon.(\varphi.AW_{Mn})^{-1}.10^6$	$k_{MnO2H2S}$	$mM^{-1}.yr^{-1}$
26		$FeOOH + 0.5H_2S \rightarrow S^0 + Fe^{2+}$	$k_{Fe(OH)3H2S} \cdot [Fe(OH)_3] \cdot [H_2S]. \rho_s.\varepsilon.(\varphi.AW_{Fe})^{-1}.10^6$	$k_{Fe(OH)3H2S}$**	$mM^{-1}.yr^{-1}$
27		$Fe^{HR} \rightarrow Fe^{MR}$	$k_{Fe(OH)3HRMR}. [Fe(OH)_3{}^{HR}]$	$k_{Fe(OH)3HRMR}$	yr^{-1}
28		$S^0 \rightarrow 0.25SO_4^{2-} + 0.75H_2S$	$k_{S0}.[S^0]$	k_{S0}	yr^{-1}

* Throughout the table, the term “DOC” applies to both DOC and GPS pools.

**Applied to both highly reactive and moderately reactive iron species of $Fe(OH)_3$.

L_2=0.59 and AW is the atomic weight. Squared parentheses stand for the concentration of the compound.

References

1 Babakhani, P., Bridge, J., Doong, R. a. & Phenrat, T. Parameterization and prediction of nanoparticle transport in porous media: A reanalysis using artificial neural network. *Water Resources Research* **53**, 4564-4585 (2017).

2 Jørgensen, B. B. A comparison of methods for the quantification of bacterial sulfate reduction in coastal marine sediments: I. Measurement with radiotracer techniques. *Geomicrobiology Journal* **1**, 11-27 (1978).

3 Burdige, D. J. & Gardner, K. G. Molecular weight distribution of dissolved organic carbon in marine sediment pore waters. *Marine Chemistry* **62**, 45-64 (1998).

4 Burdige, D. J., Komada, T., Magen, C. & Chanton, J. P. Modeling studies of dissolved organic matter cycling in Santa Barbara Basin (CA, USA) sediments. *Geochimica et Cosmochimica Acta* **195**, 100-119 (2016).

5 Dittmar, T. *et al.* Enigmatic persistence of dissolved organic matter in the ocean. *Nature Reviews Earth & Environment*, 1-14 (2021).

6 Amon, R. M. W. & Benner, R. Bacterial utilization of different size classes of dissolved organic matter. *Limnology and Oceanography* **41**, 41-51 (1996).

7 Benner, R. & Amon, R. M. W. The size-reactivity continuum of major bioelements in the ocean. *Annual review of marine science* **7**, 185-205 (2015).

8 Verdugo, P. *et al.* The oceanic gel phase: a bridge in the DOM–POM continuum. *Marine Chemistry* **92**, 67-85 (2004).

9 Arrieta, J. M. *et al.* Dilution limits dissolved organic carbon utilization in the deep ocean. *Science* **348**, 331-333 (2015).

10 Middelburg, J. J. Escape by dilution. *Science* **348**, 290-290 (2015).

11 Hemingway, J. D. *et al.* Mineral protection regulates long-term global preservation of natural organic carbon. *Nature* **570**, 228-231, doi:10.1038/s41586-019-1280-6 (2019).

12 Keil, R. G., Montluçon, D. B., Prahl, F. G. & Hedges, J. I. Sorptive preservation of labile organic matter in marine sediments. *Nature* **370**, 549-552 (1994).

13 Hedges, J. I. & Keil, R. G. Sedimentary organic matter preservation: an assessment and speculative synthesis. *Marine chemistry* **49**, 81-115 (1995).

14 Alperin, M. J., Albert, D. B. & Martens, C. S. Seasonal variations in production and consumption rates of dissolved organic carbon in an organic-rich coastal sediment. *Geochimica et Cosmochimica Acta* **58**, 4909-4930 (1994).

15 Ren, W. *et al.* Global sensitivity study of non-reactive and sorptive solute dispersivity in multiscale heterogeneous sediments. *Journal of Hydrology* **619**, 129274 (2023).

16 Massoudieh, A., Bombardelli, F. A. & Ginn, T. R. A biogeochemical model of contaminant fate and transport in river waters and sediments. *Journal of contaminant hydrology* **112**, 103-117 (2010).

17 Burdige, D. J. Preservation of organic matter in marine sediments: controls, mechanisms, and an imbalance in sediment organic carbon budgets? *Chemical reviews* **107**, 467-485 (2007).

18 Dale, A. W. *et al.* A revised global estimate of dissolved iron fluxes from marine sediments. *Global Biogeochemical Cycles* **29**, 691-707 (2015).

19 Burdige, D. J., Komada, T., Magen, C. & Chanton, J. P. Methane dynamics in Santa Barbara Basin (USA) sediments as examined with a reaction-transport model. *Journal of Marine Research* **74**, 277-313 (2016).

20 Leong, L. P. & Wedzicha, B. L. A critical appraisal of the kinetic model for the Maillard browning of glucose with glycine. *Food Chemistry* **68**, 21-28 (2000).

21 Babakhani, P. *et al.* Comparison of a new mass-concentration, chain-reaction model with the population-balance model for early-and late-stage aggregation of shattered graphene oxide nanoparticles. *Colloids and Surfaces A: Physicochemical and Engineering Aspects* **582**, 123862 (2019).

22 Rogalinski, T., Liu, K., Albrecht, T. & Brunner, G. Hydrolysis kinetics of biopolymers in subcritical water. *The Journal of Supercritical Fluids* **46**, 335-341 (2008).

23 Hansell, D. A. Recalcitrant dissolved organic carbon fractions. *Annual review of marine science* **5**, 421-445 (2013).

24 Babakhani, P., Bridge, J., Doong, R.-a. & Phenrat, T. Continuum-based models and concepts for the transport of nanoparticles in saturated porous media: A state-of-the-science review. *Advances in Colloid and Interface Science* **246**, 75-104, doi:https://doi.org/10.1016/j.cis.2017.06.002 (2017).

25 Šimunek, J., Šejna, M., Saito, H., Sakai, M. & Van Genuchten, M. T. The HYDRUS-1D software package for simulating the one-dimensional movement of water, heat, and multiple solutes in variably-saturated media, version 4.08. *University of California, Riverside, Dept. of Environmental Sciences HYDRUS Software Series* **3**, 330 (2009).

26 Berner, R. A. Inclusion of adsorption in the modelling of early diagenesis. *Earth and Planetary Science Letters* **29**, 333-340 (1976).

27 Corapcioglu, M. Y. & Jiang, S. Colloid-facilitated groundwater contaminant transport. *Water Resources Research* **29**, 2215-2226 (1993).

28 Sohma, A., Sekiguchi, Y. & Nakata, K. Modeling and evaluating the ecosystem of sea-grass beds, shallow waters without sea-grass, and an oxygen-depleted offshore area. *Journal of marine systems* **45**, 105-142 (2004).

29 Babakhani, P. The impact of nanoparticle aggregation on their size exclusion during transport in porous media: One-and three-dimensional modelling investigations. *Scientific reports* **9**, 1-12 (2019).

30 Zheng, C. & Wang, P. P. A modular three-dimensional multi-species transport model for simulation of advection, dispersion and chemical reactions of contaminants in groundwater systems; documentation and user's guide. *US Army Engineer Research and Development Center Contract Report SERDP-99-1, Vicksburg, Mississippi, USA* (1999).

31 Robador, A., Brüchert, V., Steen, A. D. & Arnosti, C. Temperature induced decoupling of enzymatic hydrolysis and carbon remineralization in long-term incubations of Arctic and temperate sediments. *Geochimica et Cosmochimica Acta* **74**, 2316-2326 (2010).

32 Bradley, J. A. *et al.* Widespread energy limitation to life in global subseafloor sediments. *Science advances* **6**, eaba0697 (2020).

33 Arndt, S. *et al.* Quantifying the degradation of organic matter in marine sediments: A review and synthesis. *Earth-Science Reviews* **123**, 53-86, doi:10.1016/j.earscirev.2013.02.008 (2013).

34 Boudreau, B. P. & Ruddick, B. R. On a reactive continuum representation of organic matter diagenesis. *American Journal of Science* **291**, 507-538 (1991).

35 Killops, S. D. & Killops, V. J. *Introduction to organic geochemistry*. (John Wiley & Sons, 2013).

36 Cole, J. J., McDowell, W. H. & Likens, G. E. Sources and molecular weight of" dissolved" organic carbon in an oligotrophic lake. *Oikos*, 1-9 (1984).

37 Kukulka, D. J., Gebhart, B. & Mollendorf, J. C. in *Advances in Heat Transfer* Vol. 18 (eds James P. Hartnett & Thomas F. Irvine) 325-363 (Elsevier, 1987).

38 Boudreau, B. P. *Diagenetic models and their implementation*. Vol. 410 (Springer, Berlin, 1997).

39 Weast, R. C., Astle, M. J. & Beyer, W. H. CRC Handbook of Chemistry and Physics. 70th eds. *Boca Raton, Frorida* (1989).

40 Razavi, S. & Gupta, H. V. A new framework for comprehensive, robust, and efficient global sensitivity analysis: 1. Theory. *Water Resources Research* **52**, 423-439 (2016).

41 Morris, M. D. Factorial sampling plans for preliminary computational experiments. *Technometrics* **33**, 161-174 (1991).

42 Sobol, I. y. M. On sensitivity estimation for nonlinear mathematical models. *Matematicheskoe modelirovanie* **2**, 112-118 (1990).

43 Jaxa-Rozen, M. & Kwakkel, J. Tree-based ensemble methods for sensitivity analysis of environmental models: A performance comparison with Sobol and Morris techniques. *Environmental Modelling & Software* **107**, 245-266 (2018).

44 Tunkiel, A. T., Sui, D. & Wiktorski, T. Data-driven sensitivity analysis of complex machine learning models: A case study of directional drilling. *Journal of Petroleum Science and Engineering* **195**, 107630 (2020).

45 Razavi, S. & Gupta, H. V. What do we mean by sensitivity analysis? The need for comprehensive characterization of "global" sensitivity in E arth and E nvironmental systems models. *Water Resources Research* **51**, 3070-3092 (2015).

46 Wang, A. & Solomatine, D. P. Practical experience of sensitivity analysis: Comparing six methods, on three hydrological models, with three performance criteria. *Water* **11**, 1062 (2019).

47 Zhang, P. A novel feature selection method based on global sensitivity analysis with application in machine learning-based prediction model. *Applied Soft Computing* **85**, 105859 (2019).

48 Berg, P., Rysgaard, S. & Thamdrup, B. Dynamic modeling of early diagenesis and nutrient cycling. A case study in an artic marine sediment. *American journal of science* **303**, 905-955 (2003).

49 Fossing, H. *et al.* A model set-up for an oxygen and nutrient flux model for Aarhus Bay (Denmark). *NERI technical report* **483** (2004).

50 Kraal, P., Slomp, C. P., Reed, D. C., Reichart, G. J. & Poulton, S. W. Sedimentary phosphorus and iron cycling in and below the oxygen minimum zone of the northern Arabian Sea. *Biogeosciences* **9**, 2603-2624 (2012).

51 Wijsman, J. W. M., Herman, P. M. J., Middelburg, J. J. & Soetaert, K. A model for early diagenetic processes in sediments of the continental shelf of the Black Sea. *Estuarine, Coastal and Shelf Science* **54**, 403-421 (2002).

52 Reed, D. C., Slomp, C. P. & de Lange, G. J. A quantitative reconstruction of organic matter and nutrient diagenesis in Mediterranean Sea sediments over the Holocene. *Geochimica et Cosmochimica Acta* **75**, 5540-5558 (2011).

53 Reed, D. C., Slomp, C. P. & Gustafsson, B. G. Sedimentary phosphorus dynamics and the evolution of bottom-water hypoxia: A coupled benthic–pelagic model of a coastal system. *Limnology and Oceanography* **56**, 1075-1092 (2011).

54 Meysman, F. J. R., Middelburg, J. J., Herman, P. M. J. & Heip, C. H. R. Reactive transport in surface sediments. II. Media: an object-oriented problem-solving environment for early diagenesis. *Computers & Geosciences* **29**, 301-318 (2003).

55 Kasih, G. A. A., Chiba, S., Yamagata, Y., Shimizu, Y. & Haraguchi, K. Numerical model on the material circulation for coastal sediment in Ago Bay, Japan. *Journal of Marine Systems* **77**, 45-60 (2009).

56 Burwicz, E. B., Rüpke, L. H. & Wallmann, K. Estimation of the global amount of submarine gas hydrates formed via microbial methane formation based on numerical reaction-transport modeling and a novel parameterization of Holocene sedimentation. *Geochimica et Cosmochimica Acta* **75**, 4562-4576 (2011).

57 Restreppo, G. A., Wood, W. T. & Phrampus, B. J. Oceanic sediment accumulation rates predicted via machine learning algorithm: towards sediment characterization on a global scale. *Geo-Marine Letters* **40**, 755-763 (2020).

58 Lee, T. R., Wood, W. T. & Phrampus, B. J. A machine learning (kNN) approach to predicting global seafloor total organic carbon. *Global Biogeochemical Cycles* **33**, 37-46 (2019).

59 NOAA, N. O. a. A. A. National Centers for Environmental Information, World Ocean Atlas (WOA). *https://www.nodc.noaa.gov/OC5/woa18/woa18data.html* (2018).

60 Bohlen, L., Dale, A. W. & Wallmann, K. Simple transfer functions for calculating benthic fixed nitrogen losses and C: N: P regeneration ratios in global biogeochemical models. *Global biogeochemical cycles* **26** (2012).

61 Couto, P. R. G., Damasceno, J. C. & de Oliveira, S. P. in *Theory and Applications of Monte Carlo Simulations* (ed Victor (Wai Kin) Chan) (INTECH Open Access Publisher, 2013).

62 McCulloch, W. S. & Pitts, W. A logical calculus of the ideas immanent in nervous activity. *The bulletin of mathematical biophysics* **5**, 115-133 (1943).

63 Yu, H. *et al.* Prediction of the Particle Size Distribution Parameters in a High Shear Granulation Process Using a Key Parameter Definition Combined Artificial Neural Network Model. *Industrial & Engineering Chemistry Research* **54**, 10825-10834, doi:10.1021/acs.iecr.5b02679 (2015).

64 Nourani, V. & Sayyah-Fard, M. Sensitivity analysis of the artificial neural network outputs in simulation of the evaporation process at different climatologic regimes. *Advances in Engineering Software* **47**, 127-146 (2012).

65 Gevrey, M., Dimopoulos, I. & Lek, S. Two-way interaction of input variables in the sensitivity analysis of neural network models. *Ecological modelling* **195**, 43-50 (2006).

66 Yerramareddy, S., Lu, S. C. Y. & Arnold, K. F. Developing empirical models from observational data using artificial neural networks. *Journal of Intelligent Manufacturing* **4**, 33-41 (1993).

67 Gevrey, M., Dimopoulos, I. & Lek, S. Review and comparison of methods to study the contribution of variables in artificial neural network models. *Ecological modelling* **160**, 249-264 (2003).

68 Doherty, J. *Pest, Model-Independent Parameter Estimation User Manual: 5th Edition.*, (Watermark Numerical Computing, 2004).

69 Wallmann, K. *et al.* The global inventory of methane hydrate in marine sediments: A theoretical approach. *Energies* **5**, 2449-2498 (2012).

70 Babakhani, P., Bridge, J., Phenrat, T., Doong, R.-a. & Whittle, K. R. Aggregation and sedimentation of shattered graphene oxide nanoparticles in dynamic environments: a solid-body rotational approach. *Environmental Science: Nano* **5**, 1859-1872 (2018).

71 Nash, J. E. & Sutcliffe, J. V. River flow forecasting through conceptual models part I — A discussion of principles. *Journal of Hydrology* **10**, 282-290, doi:10.1016/0022-1694(70)90255-6 (1970).

72 Philibert, J. One and a half century of diffusion: Fick, Einstein before and beyond. (2006).

73 Bahr, J. M. & Rubin, J. Direct comparison of kinetic and local equilibrium formulations for solute transport affected by surface reactions. *Water Resources Research* **23**, 438-452 (1987).

74 Akaike, H. A new look at the statistical model identification. *Automatic Control, IEEE Transactions on* **19**, 716-723 (1974).

75 Bradford, S. A., Torkzaban, S., Leij, F. & Simunek, J. Equilibrium and kinetic models for colloid release under transient solution chemistry conditions. *Journal of contaminant hydrology* **181**, 141-152 (2015).

76 Saiers, J. E. & Hornberger, G. M. Migration of 137Cs through quartz sand: experimental results and modeling approaches. *Journal of contaminant hydrology* **22**, 255-270 (1996).

77 Koeppenkastrop, D. & De Carlo, E. H. Uptake of rare earth elements from solution by metal oxides. *Environmental science & technology* **27**, 1796-1802 (1993).

78 Langmuir, D. Aqueous environmental. *Geochemistry Prentice Hall: Upper Saddle River, NJ* **600** (1997).

79 Limousin, G. *et al.* Sorption isotherms: A review on physical bases, modeling and measurement. *Applied Geochemistry* **22**, 249-275, doi:http://dx.doi.org/10.1016/j.apgeochem.2006.09.010 (2007).

80 Moore, O. W. *et al.* Long-term organic carbon preservation enhanced by iron and manganese. *Nature* **621**, 312-317, doi:10.1038/s41586-023-06325-9 (2023).

81 Canfield, D. E. Factors influencing organic carbon preservation in marine sediments. *Chemical geology* **114**, 315-329 (1994).

82 Rothman, D. H. & Forney, D. C. Physical model for the decay and preservation of marine organic carbon. *Science* **316**, 1325-1328, doi:10.1126/science.1138211 (2007).

83 Lalonde, K., Mucci, A., Ouellet, A. & Gélinas, Y. Preservation of organic matter in sediments promoted by iron. *Nature* **483**, 198-200 (2012).

84 Henrichs, S. M. Sedimentary organic matter preservation: an assessment and speculative synthesis—a comment. *Marine Chemistry* **49**, 127-136 (1995).

85 Lee, C. Kitty litter for carbon control. *Nature* **370**, 503-504 (1994).

86 Hedges, J. I. The formation and clay mineral reactions of melanoidins. *Geochimica et Cosmochimica Acta* **42**, 69-76 (1978).

87 Kennedy, M. J., Pevear, D. R. & Hill, R. J. Mineral surface control of organic carbon in black shale. *Science* **295**, 657-660 (2002).

88 LaRowe, D. E. *et al.* The fate of organic carbon in marine sediments-New insights from recent data and analysis. *Earth-Science Reviews* **204**, 103146 (2020).

89 Kleber, M. *et al.* Dynamic interactions at the mineral–organic matter interface. *Nature Reviews Earth & Environment* **2**, 402-421 (2021).

90 Keil, R. G. & Mayer, L. M. in *Treatise on Geochemistry (Second Edition)* (eds Heinrich D. Holland & Karl K. Turekian) 337-359 (Elsevier, 2014).

91 Honjo, S., Manganini, S. J., Krishfield, R. A. & Francois, R. Particulate organic carbon fluxes to the ocean interior and factors controlling the biological pump: A synthesis of global sediment trap programs since 1983. *Progress in Oceanography* **76**, 217-285 (2008).

92 Henson, S. A. *et al.* A reduced estimate of the strength of the ocean's biological carbon pump. *Geophysical Research Letters* **38** (2011).

93 DeVries, T. The oceanic anthropogenic CO2 sink: Storage, air-sea fluxes, and transports over the industrial era. *Global Biogeochemical Cycles* **28**, 631-647 (2014).

94 Mills, B. J. W., Donnadieu, Y. & Godderis, Y. Spatial continuous integration of Phanerozoic global biogeochemistry and climate. *Gondwana Research* **100**, 73-86 (2021).

95 Aumont, O., Éthé, C., Tagliabue, A., Bopp, L. & Gehlen, M. PISCES-v2: an ocean biogeochemical model for carbon and ecosystem studies. *Geoscientific Model Development Discussions* **8** (2015).

96 Kasim, M. F. *et al.* Building high accuracy emulators for scientific simulations with deep neural architecture search. *Machine Learning: Science and Technology* **3**, 015013 (2021).

97 Lower, S. K., Hochella Jr, M. F. & Beveridge, T. J. Bacterial recognition of mineral surfaces: nanoscale interactions between Shewanella and α-FeOOH. *Science* **292**, 1360-1363 (2001).

98 Xiao, K.-Q., Moore, O. W., Babakhani, P., Curti, L. & Peacock, C. L. Mineralogical control on methylotrophic methanogenesis and implications for cryptic methane cycling in marine surface sediment. *Nature Communications* **13**, 2722 (2022).

99 Thullner, M., Dale, A. W. & Regnier, P. Global-scale quantification of mineralization pathways in marine sediments: A reaction-transport modeling approach. *Geochemistry, Geophysics, Geosystems* **10**, n/a-n/a, doi:10.1029/2009gc002484 (2009).

100 LaRowe, D. E. *et al.* Organic carbon and microbial activity in marine sediments on a global scale throughout the Quaternary. *Geochimica et Cosmochimica Acta* **286**, 227-247 (2020).
